# Bivariate network meta‐analysis for surrogate endpoint evaluation

**DOI:** 10.1002/sim.8187

**Published:** 2019-05-26

**Authors:** Sylwia Bujkiewicz, Dan Jackson, John R. Thompson, Rebecca M. Turner, Nicolas Städler, Keith R. Abrams, Ian R. White

**Affiliations:** ^1^ Biostatistics Research Group, Department of Health Sciences University of Leicester Leicester UK; ^2^ Statistical Innovation Group Astrazeneca Cambridge UK; ^3^ Genetic Epidemiology Group, Department of Health Sciences University of Leicester Leicester UK; ^4^ MRC Clinical Trials Unit University College London London UK; ^5^ Roche Innovation Center F. Hoffmann‐La Roche Ltd Basel Switzerland

**Keywords:** Bayesian analysis, multivariate meta‐analysis, network meta‐analysis, surrogate endpoints

## Abstract

Surrogate endpoints are very important in regulatory decision making in healthcare, in particular if they can be measured early compared to the long‐term final clinical outcome and act as good predictors of clinical benefit. Bivariate meta‐analysis methods can be used to evaluate surrogate endpoints and to predict the treatment effect on the final outcome from the treatment effect measured on a surrogate endpoint. However, candidate surrogate endpoints are often imperfect, and the level of association between the treatment effects on the surrogate and final outcomes may vary between treatments. This imposes a limitation on methods which do not differentiate between the treatments. We develop bivariate network meta‐analysis (bvNMA) methods, which combine data on treatment effects on the surrogate and final outcomes, from trials investigating multiple treatment contrasts. The bvNMA methods estimate the effects on both outcomes for all treatment contrasts individually in a single analysis. At the same time, they allow us to model the trial‐level surrogacy patterns within each treatment contrast and treatment‐level surrogacy, thus enabling predictions of the treatment effect on the final outcome either for a new study in a new population or for a new treatment. Modelling assumptions about the between‐studies heterogeneity and the network consistency, and their impact on predictions, are investigated using an illustrative example in advanced colorectal cancer and in a simulation study. When the strength of the surrogate relationships varies across treatment contrasts, bvNMA has the advantage of identifying treatment comparisons for which surrogacy holds, thus leading to better predictions.

## 1 INTRODUCTION

Surrogate endpoints are very important in the drug development process, at both the trial design and the evaluation stage. They are particularly useful when they can provide early measurement of the treatment effect, in settings where a long follow‐up time is required before measurement of the final clinical outcome.^1^ This is often the case in cancer where overall survival is of primary interest whilst other outcomes such as progression‐free survival (PFS) potentially can be used to measure the effect of a treatment earlier. Alternatively, PFS may be of primary interest and tumour response (TR) is then investigated as a short term surrogate endpoint to PFS. Before they can be used in evaluation of new health technologies, candidate surrogate endpoints have to be assessed for their predictive value of the treatment effect on the final clinical outcome. Surrogate outcomes are validated by estimating the pattern of association between the treatment effects on surrogate and final endpoints across trials, in different populations and/or investigating different treatments, using meta‐analytic techniques.^2^, ^3^, ^4^, ^5^, ^6^, ^7^

Multivariate meta‐analysis methods are used to obtain average treatment effects on multiple endpoints while taking account of the correlations between them^8^, ^9^, ^10^, ^11^ and, as such, are suitable tools for modelling surrogate endpoints.^6^, ^7^ Bivariate meta‐analysis of treatment effects on a surrogate and a final outcome allows for both the validation of a surrogate endpoint and for making predictions of an unobserved treatment effect on the final clinical outcome from observed treatment effects on a surrogate endpoint.

Candidate surrogate endpoints often are not perfect, and the association patterns between the treatment effects on the surrogate and final outcomes may vary between treatments. Whilst bivariate meta‐analysis, described in detail in Section 3, can be used to model surrogate endpoints, it does not differentiate between the treatment options. Network meta‐analysis (NMA) combines data from trials investigating heterogeneous treatment contrasts and has the advantage of estimating effects for all treatment contrasts individually. At the same time, the consistency assumption allows for combining evidence (and borrowing strength) across the treatment contrasts. In this paper, we will exploit this framework to model surrogacy relationships by building on the previously reported methods of multivariate NMA (mvNMA).^12^, ^13^, ^14^, ^15^, ^16^

In the mvNMA, true treatment effects on multiple correlated outcomes are assumed to follow separate multivariate distributions for each treatment contrast. The previously reported mvNMA methods typically simplified the between‐studies variance‐covariance structures by assuming homogeneity of the correlations and the heterogeneity parameters across treatment contrasts. We relax this assumption of homogeneity to model in detail different association patterns between the effects on surrogate and final endpoints separately for different treatments or treatment classes. By allowing such patterns to differ across treatments, the methodology can help to identify treatments for which the surrogacy holds and to improve predictions. The network consistency assumption provides a framework for combining evidence across a range of treatment contrasts and hence modelling and distinguishing surrogacy patterns at the study level (within each treatment contrast) and at the treatment contrast level. These two levels of surrogacy enable predictions of the treatment effect on the final outcome in a new study investigating either an existing treatment in a new population (study‐level surrogacy within a treatment contrast) or a new treatment (treatment‐level surrogacy). We extend the second‐order consistency condition, described by Lu and Ades^17^ in a univariate NMA, to the bivariate case, in order to gain additional precision in modelling surrogate relationships.

We illustrate the use of the methods in an example in advanced colorectal cancer (aCRC) introduced in Section 2, describe the methods in Sections 3 and 4, and present the results of fitting the methods to data from the illustrative example in Section 5. To illustrate the motivation and the application of the methodology in a more detailed and controlled manner as well as to compare the performance of the methods, we carried out a simulation study presented in Section 6. We conclude this paper with a discussion in Section 7. We fit all models in a Bayesian framework using the OpenBUGS software.

## 2 ILLUSTRATIVE EXAMPLE

**Figure 1:**
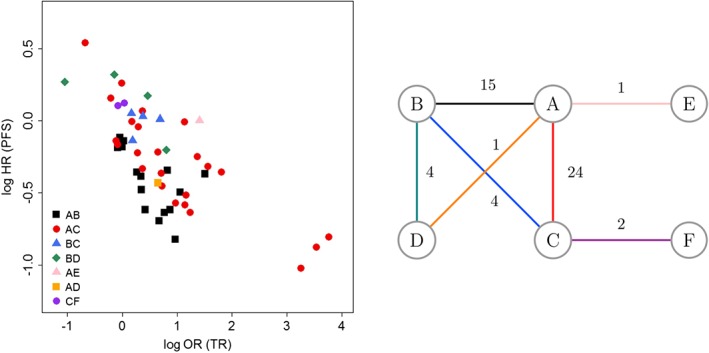
Scatter plot and network diagram for the advanced colorectal cancer example. A, Chemotherapy alone; B, Antiangiogenic treatments targeting vascular endothelial growth factor (anti‐VEGF) therapies + chemotherapy; C, Epidermal growth factor receptor inhibitor (EGFRi) + chemotherapy; D, EGFRi + anti‐VEGF therapies + chemotherapy; E, antibody targeting the type 1 insulin‐like growth factor receptor; F, antibody targeting integrin receptor + chemotherapy. HR, hazard ratio; OR, odds ratio; PFS, progression‐free survival; TR, tumour response [Colour figure can be viewed at wileyonlinelibrary.com]

We use data from randomised controlled trials (RCTs) in aCRC, investigating a range of different treatment options, to illustrate how the hierarchical bivariate NMA (bvNMA) models, proposed in this paper, can differentiate the association patterns between the treatment contrasts or classes whilst borrowing strength across treatment contrasts. Data were obtained from four published systematic reviews of RCTs investigating pharmacological treatments in aCRC, categorised into classes with respect to their mechanism of action. These were targeted therapies including antiangiogenic treatments targeting vascular endothelial growth factor (anti‐VEGF),^18^ anti epidermal growth factor receptor inhibitors (EGFRis),^19^ humanised monoclonal antibody targeting integrin receptors, and monoclonal antibody targeting the type 1 insulin‐like growth factor receptor^20^ or chemotherapies compared to the targeted therapies^20^, ^21^ and combinations of these therapies. Fifteen RCTs investigated use of anti‐VEGF with chemotherapy vs. chemotherapy alone, 24 RCTs of EGFRi with chemotherapy vs. chemotherapy alone, 4 RCTs of EGFRi with chemotherapy vs. anti‐VEGF with chemotherapy, 4 RCTs of EGFRi with anti‐VEGF and chemotherapy vs. anti‐VEGF with chemotherapy, one study (in two subgroups of population) of antibody targeting integrin receptor with EGFRi and chemotherapy vs. EGFRi with chemotherapy, one study of antibody targeting the type 1 insulin‐like growth factor receptor with chemotherapy vs. chemotherapy alone, and one of EGFRi with anti‐VEGF and chemotherapy vs. chemotherapy alone. The treatments are summarised in the network diagram of Figure 1 and the data from the individual studies are included in the Supplementary Materials. Tumour response is used as an example of a potential surrogate endpoint to PFS as a final clinical outcome. The models are applied to data representing treatment effect on the two outcomes on log odds ratio scale for TR and log hazard ratio scale for PFS. The scatter plot in Figure 1 illustrates the association patterns between the treatment effects on the two outcomes for each treatment contrast. The within‐study correlation between the treatment effects on TR and PFS, required to populate the following bivariate meta‐analytic models, was obtained from individual participant data of a RCT comparing Bevacizumab (anti‐VEGF) with chemotherapy vs. chemotherapy alone, as reported by Elia et al^22^ with further details included in the Supplementary Materials. This correlation was assumed to apply to all studies.

## 3 BIVARIATE RANDOM EFFECTS META‐ANALYSIS

The bivariate random effects meta‐analysis (BRMA) model for correlated and normally distributed treatment effects on two outcomes *Y*_1*i*_ and *Y*_2*i*_ is usually presented in the form described by Van Houwelingen et al^8^ and Riley et al^9^

(1) $$\left(\begin{array}{c} Y_{1i}\\ Y_{2i} \end{array}\right)∼N\left\{\left(\begin{array}{c} \mu_{1i}\\ \mu_{2i} \end{array}\right),\Sigma_{i}\right\},\;\Sigma_{i}=\left(\begin{array}{cc} \sigma_{1i}^{2} & \;\sigma_{1i}\sigma_{2i}\rho_{wi}\\ \sigma_{1i}\sigma_{2i}\rho_{wi} & \;\sigma_{2i}^{2} \end{array}\right)$$

(2) $$\;\left(\begin{array}{c} \mu_{1i}\\ \mu_{2i} \end{array}\right)∼N\left\{\left(\begin{array}{c} \beta_{1}\\ \beta_{2} \end{array}\right),T\right\},\;T=\left(\begin{array}{cc} \tau_{1}^{2} & \tau_{1}\tau_{2}\rho \\ \tau_{1}\tau_{2}\rho & \tau_{2}^{2} \end{array}\right).$$

In this model, the treatment effects on the surrogate endpoint *Y*_1*i*_ and on the final outcome *Y*_2*i*_ are assumed to estimate the correlated true effects *μ*_1*i*_ and *μ*_2*i*_ with corresponding within‐study variances $\sigma_{1i}^{2}$ and $\sigma_{2i}^{2}$ of the estimates and the within‐study correlation *ρ*_*wi*_ between them. In this hierarchical framework, these true study‐level effects follow a bivariate normal distribution with means $\left(\beta_{1},\beta_{2}\right)$ corresponding to the two outcomes, between‐studies variances $\tau_{1}^{2}$ and $\tau_{2}^{2}$ and a between‐studies correlation *ρ*. Equation (1) represents the within‐study model and (2) is the between‐studies model.

The elements of the within‐study covariance matrix Σ_*i*_ are assumed to be known. Whilst the estimates of the variances are easily obtained by taking the square of the standard error for each outcome, the estimates of the within‐study correlations between the treatment effects on the two outcomes are more difficult to obtain as they would not be reported in the original articles. When individual participant data (IPD) are available, the correlation can be obtained by bootstrapping^2^ or alternatively by fitting a regression model for the two outcomes with correlated errors.^23^ When IPD are available only for one study, the correlations (but not the variances or covariances) can be assumed the same for all other studies. In the absence of IPD, a range of values (between −1 and 1) can be investigated in a sensitivity analysis. Moreover, an alternative approach was proposed by Wei and Higgins who derived formulae expressing the within‐study correlations between the treatment effects (such as log odds ratios) on the two outcomes in terms of more likely reported correlations between other measures (such as probabilities of events on the two outcomes for binomial data).^24^ In the Bayesian framework, prior distributions can be placed on the within‐study correlations. Informative prior distributions for the correlations can be constructed, eg, by a double bootstrap approach, which results in the correlations being obtained with uncertainty.^11^

To implement the model in the Bayesian framework, prior distributions are placed on the mean effects, eg, vague prior distributions *β*_1_∼*N*(0,10^4^), *β*_2_∼*N*(0,10^4^) and on the between‐studies variances and correlation. In the general case, for any number of outcomes, a prior distribution has to be placed on the whole variance‐covariance matrix or the correlation matrix in such a way to ensure that the variance‐covariance matrix is positive semi‐definite. This can be achieved by placing an inverse Wishart prior distribution on the variance‐covariance matrix^10^ or by using a separation strategy, with spherical^10^, ^17^ or Cholesky decomposition^10^ of the correlation matrix. Alternatively, a product normal formulation of the between‐studies model can be used where the model is parameterised in the form of a series of univariate conditional distributions, ensuring that the relationships between the parameters of the model (regression coefficients and conditional variances) and the elements of the between‐studies variance‐covariance matrix result in the positive semidefinite between‐studies variance‐covariance matrix.^6^, ^7^, ^11^

In the bivariate case, such as considered here, positive semidefiniteness can be achieved by placing prior distributions on the variances, which are restricted to plausible positive values, eg, by placing uniform prior distributions on the corresponding standard deviations *τ*_*j*_∼*U*(0,2), and by choosing a prior distribution for the correlation which restricts it to values between −1 and +1. Here, we use a beta distribution to construct such a prior $\frac{\rho +1}{2}∼\mathrm{Beta}(1.5,1.5)$, as in the work of Burke et al,^25^ with details also in the Supplementary Materials.

## 4 BIVARIATE NMA

The BRMA model is typically used to obtain average effects on two correlated outcomes, for studies of the same treatment or treatment class. In the context of surrogate endpoints, data on a range of treatments are typically used. The BRMA assumes exchangeability of the treatment effects from all studies regardless of the treatment contrast, by assuming that the true effects follow a common (here bivariate normal) distribution (as in Equation (2)). This model works well for strong surrogate relationships across all treatments. However, the association pattern between treatment effects measured on the surrogate and final outcomes may depend on the treatment contrast. Network meta‐analysis can differentiate between treatment contrasts and in bivariate form can be applied to modelling such surrogate relationships within and across treatment contrasts. We first discuss general bvNMA models in Section 4.1, which describe the study‐level (within‐treatment) surrogate relationships in the network. In Section 4.2, we extend these models to allow for an additional level of surrogacy at the treatment level.

### 4.1 General bvNMA models

#### 4.1.1 Model 1a: bvNMA

To model correlated treatment effects on a surrogate endpoint and a final outcome for which the correlation varies according to the treatment contrasts, the assumption made by BRMA, that the true effects follow a common distribution, can be replaced by an assumption that the true effects corresponding to different treatment contrasts follow separate distributions. This naturally leads to relaxing the assumption of homogeneity of the between‐studies covariance matrix, allowing its elements (the between‐studies correlations *ρ*_1*kl*,2*kl*_ between the treatment effects *l* vs. *k* on the surrogate (1) and final (2) outcomes and the heterogeneity parameters for the treatment effects on the two outcomes $\tau_{1kl}^{2}$ and $\tau_{2kl}^{2}$) to vary across the treatment contrasts *kl*.

To take into account the network structure of the data, we model the treatment effect differences *Y*_*jkli*_ between treatments *k* and *l* in study *i* for outcome *j* = 1,2, as follows:

(3) $$\left(\begin{array}{c} Y_{1kli}\\ Y_{2kli} \end{array}\right)∼N\left\{\left(\begin{array}{c} \mu_{1kli}\\ \mu_{2kli} \end{array}\right),\Sigma_{i}\right\},\;\Sigma_{i}=\left(\begin{array}{cc} \sigma_{1kli}^{2} & \;\sigma_{1kli}\sigma_{2kli}\rho_{wkli}\\ \sigma_{1kli}\sigma_{2kli}\rho_{wkli} & \;\sigma_{2kli}^{2} \end{array}\right)$$

(4) $$\left(\begin{array}{c} \mu_{1kli}\\ \mu_{2kli} \end{array}\right)∼N\left\{\left(\begin{array}{c} d_{1kl}\\ d_{2kl} \end{array}\right),\;\left(\begin{array}{cc} \tau_{1kl}^{2} & \tau_{1kl}\tau_{2kl}\rho_{1kl,2kl}\\ \tau_{1kl}\tau_{2kl}\rho_{1kl,2kl} & \tau_{2kl}^{2} \end{array}\right)\right\},$$

where *k* and *l* denote baseline (control) and experimental treatments, respectively, in a study *i*, *μ*_*jkli*_ denote the random true treatment effects (differences between the effects of treatments *k* and *l*) on outcome *j* in study *i*, and the *d*_*jkl*_ are mean treatment effect differences between treatments *k* and *l* for each outcome *j*. We use the first‐order consistency assumption, as described by Lu and Ades,^17^ extended here to the bivariate case. For any three treatments (*b*,*k*,*l*), the treatment differences **(** *μ*_*jkli*_) satisfy the following transitivity relations:

(5) $$\left(\begin{array}{c} \mu_{1kli}\\ \mu_{2kli} \end{array}\right)=\left(\begin{array}{c} \mu_{1bli}-\mu_{1bki}\\ \mu_{2bli}-\mu_{2bki} \end{array}\right).$$

Taking the expectation of the transitivity equations gives the consistency equations for the first‐order moments

(6) $$\left(\begin{array}{c} d_{1kl}\\ d_{2kl} \end{array}\right)=\left(\begin{array}{c} d_{1bl}-d_{1bk}\\ d_{2bl}-d_{2bk}, \end{array}\right)$$

which represent the relationships between the treatment contrasts in the population. When *b* = 1 is a common reference treatment in the network, the treatment effects of each treatment *k* relative to this common reference treatment 1; the *d*_*j*1*k*_ are referred to as basic parameters for each outcome *j*, with *d*_*j*11_ = 0 and the others are given prior distributions

(7) $$d_{j1k}∼N(0,10^{3}).$$

Prior distributions are also placed on the elements of the between‐studies variance‐covariance matrices. As in BRMA, prior distributions for the heterogeneity parameters are selected to ensure that they are restricted to plausible positive values, such as *τ*_*jkl*_∼*unif*(0,2) and for the correlations to ensure restriction to the values between −1 and 1, eg, $\frac{\rho_{kl}+1}{2}∼\text{Beta}(1.5,1.5)$, thus guaranteeing that the variance‐covariance matrix for each treatment contrast is positive semidefinite.

The aforementioned model is a simplified bivariate and two‐arm study version of the mvNMA multi‐arm model by Achana et al,^12^ with the exception of retaining the heterogeneous between‐studies correlations and variances across contrasts (Achana et al introduced a general mvNMA model but then simplify it by assuming homogeneous between‐studies covariances, whilst in our model, those heterogeneous parameters are of interest). It is also a simpler version of the mvNMA model by Hong et al,^15^ however differently implemented, with random effects here being the relative effects for actual contrasts in each study rather than the effects of each treatments relative to the common baseline.

#### 4.1.2 Model 1b: bvNMA with second‐order consistency

The aforementioned meta‐analytic model 1a assumes consistency of treatment effects on both outcomes. The idea of consistency also implies some constraints on the between‐studies variance‐covariance matrices, which can be explicitly introduced to the bvNMA model (3) and (4) by assuming the consistency of the second‐order moments.

To allow for the second‐order consistency, we develop model 1a further. This new model differs from previously developed mvNMA models in a number of ways. For example, Hong et al showed that, in their contrast‐based mvNMA, the second‐order consistency assumption was satisfied for each outcome individually.^15^ In contrast to this, we investigate bivariate second‐order consistency by deriving the relationships between the covariances for any three treatments (*b*,*k*,*l*) in the network. In addition, unlike in the models on multi‐arm trials such as by Achana et al^12^ or Hong et al,^15^ when modeling data from two‐arm studies, the correlation between treatment arms does not enter the likelihood and hence is not included in the model. The relationships between the covariances are therefore not guaranteed, since a prior distribution is not placed on the covariance matrix directly, and instead need to be modelled explicitly.

To model the bivariate second‐order consistency, we extend the approach proposed by Lu and Ades^17^ to the bivariate case by taking variance of the transitivity equation (5), which gives

(8) $$\left(\begin{array}{cc} \tau_{1kl}^{2} & \;\tau_{1kl}\tau_{2kl}\rho_{1kl,2kl}\\ \tau_{1kl}\tau_{2kl}\rho_{1kl,2kl} & \;\tau_{2kl}^{2} \end{array}\right)=\left(\begin{array}{cc} \text{var}(\mu_{1bli}-\mu_{1bki}) & \;\text{cov}(\mu_{1bli}-\mu_{1bki},\mu_{2bli}-\mu_{2bki})\\ \text{cov}(\mu_{1bli}-\mu_{1bki},\mu_{2bli}-\mu_{2bki}) & \;\text{var}(\mu_{2bli}-\mu_{2bki}) \end{array}\right)$$

leading to the following relationship between the variances for any three treatments (*b*,*k*,*l*) and for both outcomes *j* = 1,2:

(9) $$\tau_{jkl}^{2}=\tau_{jbk}^{2}+\tau_{jbl}^{2}-2\rho_{jbk,jbl}\tau_{jbk}\tau_{jbl}\leq {\left(\tau_{jbk}+\tau_{jbl}\right)}^{2},$$

which gives the second‐order consistency conditions (triangle inequalities)

(10) $$|\tau_{jbl}-\tau_{jbk}|\leq \tau_{jkl}\leq \tau_{jbl}+\tau_{jbk}.$$

In addition, the following condition (derived in more detail in the Supplementary Materials) applies to the covariances:

(11) $$\tau_{1kl}\tau_{2kl}\rho_{1kl,2kl}=\tau_{1bl}\tau_{2bl}\rho_{1bl,2bl}+\tau_{1bk}\tau_{2bk}\rho_{1bk,2bk}-\tau_{1bl}\tau_{2bk}\rho_{1bl,2bk}-\tau_{1bk}\tau_{2bl}\rho_{1bk,2bl},$$

which implies further constraints that are more complex than those in Equation (10).

To ensure that prior distributions for heterogeneous variance‐covariance matrices are appropriate, ie, to maintain the second‐order consistency condition for any three treatments in the network, bivariate ancillary parameters are used, extending the univariate model by Lu and Ades,^17^ allowing the between‐studies variance‐covariance matrices to be represented as

(12) $$\left(\begin{array}{cc} \tau_{1kl}^{2} & \;\tau_{1kl}\tau_{2kl}\rho_{1kl,2kl}\\ \tau_{1kl}\tau_{2kl}\rho_{1kl,2kl} & \tau_{2kl}^{2} \end{array}\right)=\left(\begin{array}{cc} \gamma_{1k}^{2}+\gamma_{1l}^{2}-2\xi_{1k,1l}\gamma_{1k}\gamma_{1l} & \begin{array}{c} \gamma_{1k}\gamma_{2k}\xi_{1k,2k}-\gamma_{1k}\gamma_{2l}\xi_{1k,2l}\\ -\gamma_{1l}\gamma_{2k}\xi_{1l,2k}+\gamma_{1l}\gamma_{2l}\xi_{1l,2l} \end{array}\\ \begin{array}{c} \gamma_{1k}\gamma_{2k}\xi_{1k,2k}-\gamma_{1k}\gamma_{2l}\xi_{1k,2l}\\ -\gamma_{1l}\gamma_{2k}\xi_{1l,2k}+\gamma_{1l}\gamma_{2l}\xi_{1l,2l} \end{array} & \gamma_{2k}^{2}+\gamma_{2l}^{2}-2\xi_{2k,2l}\gamma_{2k}\gamma_{2l} \end{array}\right),$$

where the ancillary parameters $\gamma_{jk}^{2}$ and $\gamma_{jl}^{2}$ are variances of two random effects *ζ*_*jki*_ and *ζ*_*jli*_ corresponding to treatment arms *k* and *l* (for each outcome *j* = 1,2), and $\xi_{jk,j^{\prime }l}$ is their correlation coefficient. Prior distributions for the set of between‐studies standard deviations *τ*_*jkl*_ for each outcome *j* and each pair of treatments *k* and *l* can be given by constructing a prior distribution for a covariance matrix Γ composed of the standard deviations *γ*_*jk*_ and the correlations $\xi_{jk,j^{\prime }l}$ between the effects *ζ*_*jki*_ and $\zeta_{j^{\prime }li}$, for *j*,*j* *′* = 1,2 and *k*,*l* = 1,…,*n*_*t*_, where *n*_*t*_ is the number of treatments in the network. For the set of values of the elements of matrix Γ to give a resulting set of standard deviations *τ*_*jkl*_ that satisfy the second‐order consistency rules (10) and (11), the matrix Γ has to be positive semidefinite. This is achieved using a separation strategy with a Cholesky decomposition Γ = *V* ^1/2^ *RV* ^1/2^, where *V* ^1/2^ is a 2*n*_*t*_ × 2*n*_*t*_ diagonal matrix of the standard deviations $\gamma_{11},\gamma_{21},\text{…},\gamma_{1n_{t}},\gamma_{2n_{t}}$ and *R* is a positive semidefinite 2*n*_*t*_ × 2*n*_*t*_ matrix of correlations $\xi_{jk,j^{\prime }l}$ (block matrix consisting of *n*_*t*_ × *n*_*t*_ blocks that are of 2 × 2 dimension). Matrix *R* is represented as *R* = *L* ^*T*^ *L* with *L* being a 2*n*_*t*_ × 2*n*_*t*_ upper triangular matrix. Further details on the derivation of this model and the construction of the prior distributions are included in the Supplementary Materials.

#### 4.1.3 Model 1c: bvNMA with second‐order consistency and similarity of the ancillary parameters

To ensure second‐order consistency of the treatment effects in the network, model 1b requires estimation of relatively many parameters. Where data for each treatment *k* is only available from a limited number of studies, it may be difficult to estimate the individual variances $\gamma_{1k}^{2}$. To overcome this issue, exchangeability of the ancillary variances may be assumed by replacing individual prior distributions for these parameters with a common distribution

(13) $$\gamma_{jk}^{2}∼N(0,v_{j})I(0,)\;\;\;\text{and}\;\;\;v_{j}∼\Gamma (1.0,0.01).$$

#### 4.1.4 Model 1d: bvNMA assuming homogeneity of the between‐studies variance‐covariance matrix

When networks are sparse, instead of assuming similarity between the heterogeneity parameters, as in Equation (13), homogeneity of the between‐studies variance‐covariance matrix can be assumed, which is a common assumption in the mvNMA or the univariate NMA of multi‐arm trials.^26^ The between‐studies equation (4) then becomes

(14) $$\left(\begin{array}{c} \mu_{1kli}\\ \mu_{2kli} \end{array}\right)∼N\left\{\left(\begin{array}{c} d_{11l}-d_{11k}\\ d_{21l}-d_{21k} \end{array}\right),\;T=\left(\begin{array}{cc} \tau_{1}^{2} & \tau_{1}\tau_{2}\rho \\ \tau_{1}\tau_{2}\rho & \tau_{2}^{2}, \end{array}\right)\right\}$$

where *μ*_*jkli*_ is the random treatment effect difference between treatments *k* and *l* on outcome *j* in study *i* and *d*_*j*1*k*_ is the mean treatment effect difference between treatment *k* and the reference treatment 1 for outcome *j*, with *d*_*j*11_ = 0. A prior distribution is placed on each basic parameter, as in Equation (7) of model 1a. A prior distribution is also placed on the elements of the common between‐studies variance‐covariance matrix, ie, the between‐studies standard deviations, *τ*_*j*_∼*Unif*(0,2) and the correlation, $\frac{\rho +1}{2}∼\text{Beta}(1.5,1.5)$.

#### 4.1.5 Study‐level surrogacy criteria

When using the bvNMA models 1a to 1c to evaluate surrogate endpoints at the study level, within treatment contrast *kl*, perfect surrogacy means that

(15) $$\rho_{1kl,2kl}=\pm 1\;\;\text{and}\;\;\mu_{1kli}=0\;\Leftrightarrow \;\mu_{2kli}=0.$$

These are criteria that may or may not be true and can be tested. They are equivalent to the surrogacy criteria suggested by Daniels and Hughes,^2^ who stated that, in the linear surrogate relationship, the slope measures the association between the treatment effects on the surrogate and clinical outcomes. In a perfect surrogate relationship, the slope is nonzero and the conditional variance, of the treatment effect on the final outcome conditional on the effect on the surrogate endpoint, is zero. This is equivalent to the left‐hand side of the criterion (15). In addition, Daniels and Hughes discussed the importance of zero intercept in the surrogacy criteria, explaining that if the surrogate relationship is perfect; then, for a study with zero treatment effect on the surrogate endpoint, we would also expect zero treatment effect on the final outcome. Otherwise, a candidate surrogate endpoint would not be a good surrogate as there would be, on average, a treatment effect on the clinical outcome unexplained by a treatment effect on the surrogate endpoint. In our models, the intercept is not explicitly present, but can be estimated from other parameters, *λ*_0_ = *d*_2*kl*_ − *d*_1*kl*_ *ρ*_1*kl*,2*kl*_ *τ*_2*kl*_/*τ*_1*kl*_. The right‐hand‐side of criterion (15) can be tested by evaluating the intercept (to determine whether it is likely to be zero by, eg, investigating the corresponding credible interval (CrI)). The details of the relationships between the surrogacy criteria of our model and those set out by Daniels and Hughes are described in the Supplementary Materials.

This criterion describes the study‐level surrogate relationships within the treatment contrasts. The network structure, with the unique surrogate relationship for each treatment contrast, can help us to disentangle information about a surrogate relationship for a particular treatment and to make better predictions, in particular when these relationships are clearly distinct, as illustrated in the simulation study in Section 6. These individual (for each treatment contrast) study‐level surrogate relationships enable predicting the treatment effect on the final outcome from the treatment effect measured on a surrogate endpoint in a new study investigating an existing treatment in a new population.

When evaluating surrogate endpoints with model 1d, as for models 1a to 1c, the random effects are assumed to follow separate distributions for studies evaluating different treatment contrasts *kl*, but the between‐studies correlations and heterogeneity parameters are assumed to be the same across the treatment contrasts. In this case, perfect surrogacy means that

(16) $$\rho =\pm 1\;\;\text{and}\;\;\mu_{1kli}=0\;\Leftrightarrow \;\mu_{2kli}=0,$$

which differs from the criteria for the models 1a to 1c with respect to the common correlation across the treatment contrasts. The strength of the association between the treatment effects on the surrogate endpoint and the final clinical outcome will be the same across the treatment contrasts when using model 1d, as when using a pairwise meta‐analysis such as BRMA. However, assuming that the random effects for different treatment contrasts follow separate distributions in the bvNMA, we model these association patterns in more detail than when using BRMA. In addition, taking into account the network structure of the data results in borrowing of strength across the treatment contrasts when evaluating surrogate relationships.

As described above, the between‐studies correlation equal to ±1 indicates perfect surrogacy. In practice, it is difficult to quantify how large the correlation should be in order to consider the surrogate endpoint suitable to make the prediction. Some authors claimed that a high level of association is required to demonstrate surrogacy. For example, Lassere et al in their biomarker‐surrogacy evaluation schema defined such high association by the square of the between‐studies correlation (or so‐called adjusted R squared) above 0.6,^27^ and the German Institute of Quality and Efficiency in Health Care requires high correlation with the lower limit of the 95% confidence interval (CI) above 0.85.^28^ Other authors emphasised that the decision of whether the surrogate endpoint may be used to make the prediction of the clinical benefit should be based on the balance between the strength of the surrogate relationship and the need for the decision to be made about the effectiveness of the new treatment, eg, for regulatory purposes.^29^ Moreover, the strength (or weakness) of the surrogate relationship will manifest itself in the width of the predicted interval of the treatment effect on the final outcome. A smaller value of the correlation will result in a larger interval and hence increased uncertainty about the regulatory or clinical decision made based on such prediction. The implication of this is that perhaps we do not need criteria about the correlation and instead we need only look at the predictions. The evaluation of the quality of predictions can be achieved through a cross‐validation procedure, which we discuss in Section 4.3.1.

### 4.2 Bivariate NMA models with borrowing of strength across treatment contrasts

**Figure 2:**
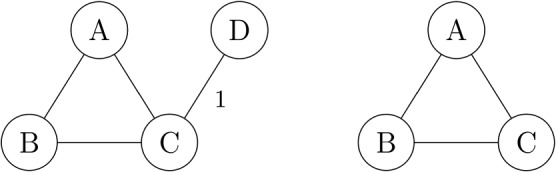
Example network diagram: data on effect of new treatment D only available in one new study and only measured on surrogate endpoint (left) but not on the final outcome (right)

Models 1a to 1d describe study‐level surrogate relationships within treatment contrasts and can be used to make predictions of a treatment effect on the final outcome (from the treatment effect measured on surrogate endpoint) in a new trial, which may investigate the treatment effects of an existing treatment but in different populations. It is assumed that there will be at least one study in the network reporting the treatment effects on both outcomes allowing to make such prediction. In the situation where we want to predict the treatment effect on the final outcome from the effect on the surrogate endpoint in a new study evaluating a new treatment, there will be only this one study reporting the effect for the new treatment and only on the surrogate endpoint. In this case, the models 1a to 1d will estimate the average effect on the final outcome for the new treatment based solely on the prior distribution, because no data are available for the effect of this treatment on the final outcome. For example, in the network depicted in Figure 2, if we select treatment A as the reference treatment 1, then the average treatment effect for the new treatment *D* on the final outcome is equal to *d*_2*CD*_ = *d*_2*AD*_ − *d*_2*AC*_ and the estimate of the basic parameter *d*_2*AD*_ would be based on the prior distribution only. As a result, the estimate of the between‐studies correlation *ρ*_1*CD*,2*CD*_ will be based solely on the prior distribution. Therefore, the predicted treatment effect on the final outcome from the treatment effect on the surrogate endpoint for the new treatment will not be meaningful.

#### 4.2.1 Models 2a to 2d: assuming exchangeability of treatments

To overcome the above issue, an exchangeability assumption can be made to allow for the relationships between the average effects on the two outcomes to be similar across treatment contrasts. To achieve this for models 1a to 1d, instead of placing a prior distribution on each basic parameter (Equation (7)), we need to add another level of hierarchy to the models. The basic parameters d_j1k_, which are the effects relative to a common reference treatment, are not independent and therefore cannot be assumed exchangeable. It is convenient to write, instead, the model with a treatment‐level effects assuming that the pooled effects for each treatment arm k, θ_jk_ on the two outcomes j = 1,2, are exchangeable and correlated

(17) $$d_{j1k}=\theta_{jk}-\theta_{j1},$$

(18) $$\left(\begin{array}{c} \theta_{1k}\\ \theta_{2k} \end{array}\right)∼N\left(\left(\begin{array}{c} \eta_{1}\\ \eta_{2} \end{array}\right),\frac{1}{2}\left(\begin{array}{cc} \omega_{1}^{2} & \omega_{1}\omega_{2}\rho_{t}\\ \omega_{1}\omega_{2}\rho_{t} & \omega_{2}^{2} \end{array}\right)\right)$$

for k = 1,…,n_t_.

The advantage of this approach is that the prior distributions for each pair (θ_1k_,θ_2k_) are independent. The modelled data Y_jkli_ are the treatment effect differences; thus, the ancillary parameters θ_jk_ are not identifiable. However, we are not interested in estimating these parameters, but in estimating the association between the average treatment effect differences on the two outcomes, ie, d_jkl_, j = 1,2. The aforementioned formulae imply the association between the average effects

(19) $$\left(\begin{array}{c} d_{1kl}\\ d_{2kl} \end{array}\right)∼N\left\{\left(\begin{array}{c} 0\\ 0 \end{array}\right),\left(\begin{array}{cc} \omega_{1}^{2} & \omega_{1}\omega_{2}\rho_{t}\\ \omega_{1}\omega_{2}\rho_{t} & \omega_{2}^{2} \end{array}\right)\right\},$$

k,l = 1,…,n_t_ (k ≠ l), cancelling out the mean values η_j_. Therefore, the mean values can be set arbitrarily to a constant, eg, η_j_ = 0. Prior distributions are placed on the elements of the covariance matrix, ie, ω_j_∼Unif(0,2) and $\frac{\rho_{t}+1}{2}∼\text{Beta}(1.5,1.5)$. Borrowing of strength across treatments by relating these parameters enables the model to predict the effect of a new treatment on the final outcome, as in the network scenario in Figure 2.

The exchangeability model (17) to (18) for the effects across treatments is combined with models 1a to 1d, by replacing the independent prior distributions on basic parameters (7), to form models 2a to 2d.

#### 4.2.2 Study‐ and treatment‐level surrogacy criteria

As for models 1a to 1d, the perfect surrogate relationship between the treatment effects on the surrogate endpoint and the final clinical outcome at the study level is described by criteria (15) and (16) for models 2a to 2c and 2d, respectively. This study‐level surrogate relationship between trials (or populations) is unique for each treatment contrast kl and it may differ across these treatment contrasts. In addition to this, another level of surrogate relationship is described by models 2a to 2d, the treatment‐level surrogacy. Such a surrogate relationship would be perfect if

(20) $$\rho_{t}=\pm 1,\;\text{and}\;d_{1kl}=0\;\Leftrightarrow \;d_{2kl}=0,$$

where the first part describes the strength of this surrogate relationship and the second ensures that, in a perfect surrogate relationship, zero average treatment effect difference between treatments k and l on the surrogate endpoint will imply zero average treatment effect also on the final clinical endpoint for the same treatment contrast. This second criterion is in fact equivalent to the exchangeability assumption in models 2a to 2d (the models assume zero intercept). This surrogate relationship allows prediction of the treatment effect on the final outcome from the treatment effect measured on a surrogate endpoint in a new study investigating a new treatment.

### 4.3 Summary and discussion of models

Bivariate models for NMA described in this section differ in their specific assumptions about the heterogeneity between studies and treatments. All components of the between‐studies models for all models, 1a to 1d and 2a to 2d, are summarised in Table 1. The models allow the correlations between the treatment effects on the surrogate and final endpoints to vary to a different degree, and which model is applied in practice can be determined based on the available evidence as well as the purpose of the model.

**Table 1:** Components of each model in terms of the assumptions made about the between‐studies variance‐covariance matrix

	Assumptions for the between‐studies variance‐covariance matrix					
	varying	second‐		homogeneity	prior	
	variance‐	order	exchangeable	of variance‐	distributions	exchangeable
NMA	‐covariance	consistency	ancillary	‐covariance	on basic	treatments
model	matrix	Equations	parameters	matrix	parameters	Equations
	Equation (4)	(10) and (11)	Equation (13)	Equation (14)	Equation (7)	(17) and (18)
1a	✓				✓	
1b	✓	✓			✓	
1c	✓	✓	✓		✓	
1d		NA		✓	✓	
2a	✓					✓
2b	✓	✓				✓
2c	✓	✓	✓			✓
2d		NA		✓		✓

Abbreviations: NMA, network meta‐analysis.

Models 1a to 1d are designed to evaluate study‐level surrogacy patterns across different trials (which may investigate the treatment effects in different populations) within each treatment contrast and make predictions of a treatment effect on the final outcome in a new study investigating an existing treatment only, but perhaps in a new population. Models 2a to 2d can be used to investigate treatment‐level surrogacy and to make predictions of an effect of a new treatment on the final clinical outcome from the effect measured on a surrogate endpoint in a new study investigating a new treatment. In addition, models 2a to 2d can be used to estimate average effects of the new treatment on the final clinical outcome relative to any of the treatments in the network, which is a unique feature of these models and cannot be achieved using BRMA or models 1a to 1d. The choice between models 1a to 1d (and similarly between 2a to 2d) can be made based on data availability and plausibility of their individual assumptions supported by investigating the model fit, eg, by the use of deviance information criteria (DIC). The plausibility of the assumptions may be verified based on the inclusion criteria for studies in the meta‐analysis. Studies in similar populations are more likely to result in treatment effects satisfying the consistency assumption and the second‐order consistency assumption could be considered as a more strict assumption about the trial populations being “especially” similar. The models reduce to the standard meta‐analysis model for surrogate endpoints, such as the BRMA model (1) to (2), in the special data structure when there are only two treatments in the network, as in the Supplementary Materials.

#### 4.3.1 Strategies for model comparison

To make comparisons between the models, in the first instance, the between‐studies correlations (as well as the mean effects and the heterogeneity parameters) are obtained and compared across the models. Following this, predicted values (and corresponding CrIs) of the treatment effects on the final outcome are compared to the observed estimates (and corresponding CIs) in take‐one‐out cross‐validation procedure. In one study at a time, the estimate of the treatment effect on the final outcome Y_2i_ is removed (and treated as missing at random), and then this treatment effect is predicted from the treatment effect on the surrogate endpoint, conditional of the data on both outcomes from all the remaining studies in the meta‐analysis. The standard deviation of the predicted effect ${Ŷ}_{2i}$ is equal to $\sqrt{\sigma_{2i}^{2}+\mathrm{var}({\hat{\mu }}_{2kli}|Y_{1i},\sigma_{1i},Y_{1(-i)},Y_{2(-i)})}$, where Y_1( − i)_ and Y_2( − i)_ denote the data from the remaining studies without the validation study i (the treatment contrast subscripts, present only in the NMA, have been dropped here).

The models were compared with respect to the predicted effects by investigating a number of statistics obtained from each model, ie, (a) proportion of the CI of the observed estimate of the effect on the final outcome overlapping with the CrI of the predicted effect, p_overlap_; (b) mean absolute difference between observed estimate of mean effect and predicted mean effect on the final outcome across studies, |m_obs_ − m_pred_|; (c) ratio of the width of intervals; the width of CrI of the predicted effect to the width of the CI of the observed effect (the estimate), w_pred_/w_obs_; (d) percentage reduction in uncertainty measured by the width of the CrI of the predicted effect when using a mvNMA model, w_NMA_, compared to the width of the CrI obtained from BRMA w_BRMA_, $\%\mathrm{red}=100\frac{w_{\mathrm{BRMA}}-w_{\mathrm{NMA}}}{w_{\mathrm{BRMA}}}$; and (e) a new statistic measuring overall performance of a model giving a higher score for models resulting in large p_overlap_ with penalty for overly inflated predicted interval $\pi =\frac{p_{\mathrm{overlap}}}{w_{\mathrm{pred}}/w_{\mathrm{obs}}}$, which is always positive and less than one.

We apply the aforementioned procedures to the individual data sets (for the illustrative example in aCRC and for some simulated toy data sets). In addition, we perform a simulation study, which is described in detail in Section 6.

## 5 RESULTS FOR aCRC DATA

In this section, we present results of applying all models (BRMA and bvNMA models 1a to 1d and 2a to 2d) to the aCRC data, introduced in Section 2. Table 2 shows the between‐studies correlations obtained from all the models applied to the aCRC data. The correlations are the parameters of the primary interest as they tell us about the strength of the surrogate relationships (see Sections 4.1.5 and 4.2.2). Additional results, the average effects, the heterogeneity parameters, and the implied intercepts are included in the Supplementary Materials. When applying BRMA to data from all studies (regardless of the treatment contrast), the between‐studies correlation is negative, −0.67 (% CrI:−0.85, −0.41), as would be expected when the surrogate outcome is a favourable event and the final outcome is an unfavourable event (treatment increasing the odds of TR leads to reduced progression rate). As shown in the scatter plot in Figure 1, the distributions of the treatment effects on the two outcomes, ie, log odds ratio of TR (surrogate endpoint) and log hazard ratio of PFS (final outcome), representing their association patterns, vary across the treatment contrasts. When applying bvNMA models 1a to 1c and 2a to 2c to the data, the between‐studies correlations differ across treatment contrasts, with the highest correlation obtained for treatment contrast AC (the results are presented only for those contrasts for which at least four studies were available, the remaining results are included in the Supplementary Materials). When applying model 1b, assuming second‐order consistency and hence imposing additional constraints on the between‐studies variance‐covariance matrices, the correlations were estimated with higher precision compared to those obtained from model 1a. Applying model 1c slightly inflated the CrIs of the correlations for some of the treatment contrasts (AC and BC) and minimally reduced them for others (AB and BD) (compared to model 1b). The DIC value, shown in the right‐hand‐side column of the table, is relatively high for model 1c indicating poor model fit. A similar pattern is observed across models 2a to 2c. When assuming homogeneous variance‐covariance matrices in models 1d and 2d, the between‐studies correlations are high and obtained with higher precision compared to the correlation obtained from BRMA. This is likely due to the borrowing of strength across treatment contrasts, by adding information from the indirect comparisons. Based on the DIC criteria, models 1d, 2a, 2b, and 2d appear to fit the data best, although models 1a and 1b appear to have a comparable fit. Prediction of the treatment effect on PFS (the final outcome) from the treatment effect on TR (the surrogate endpoint) for the treatment contrast AE, for which only one study was available (new treatment scenario), was not possible for models 1a to 1d, due to lack of data to estimate the surrogate relationship. Models 2a to 1d, assuming exchangeability of the average effects in each treatment arm across treatment contrasts, allowed us to make such a prediction. A forest plot of the observed and predicted (from BRMA and model 2d) effects on PFS is included in Figure 3 of the Supplementary Materials. The advantage of using bvNMA methods varied across the treatment contrasts, depending on the surrogate relationships for each contrast. Table 2 also shows the across‐treatment correlation ρ_t_ indicating, consistently across models 2a to 2d, a weak treatment‐level surrogate relationship.

**Table 2:** Between‐studies correlations corresponding to the study‐level surrogate relationship within each treatment contrast for each model, ρ_t_ is across‐treatment correlations describing the treatment‐level surrogacy obtained from the models allowing for exchangeability, and deviance information criteria (DIC) values corresponding to each model fitted to aCRC data. Where only one value is given for the between‐studies correlation within a treatment contrast (models BRMA, 1d and 2d), the parameters are common across the treatment contrasts

	study‐level surrogacy					
model	AB	AC	BC	BD	ρ_t_	DIC
between‐studies correlations						
BRMA	‐0.67 (‐0.85, ‐0.41)				NA	43.9
bvNMA 1a	‐0.43 (‐0.84, 0.16)	‐0.79 (‐0.95, ‐0.46)	‐0.02 (‐0.88, 0.88)	‐0.28 (‐0.94, 0.65)	NA	40.5
bvNMA 1b	‐0.61 (‐0.9, ‐0.07)	‐0.79 (‐0.95, ‐0.5)	‐0.3 (‐0.91, 0.65)	‐0.25 (‐0.89, 0.61)	NA	41.8
bvNMA 1c	‐0.6 (‐0.88, ‐0.11)	‐0.75 (‐0.93, ‐0.44)	‐0.27 (‐0.89, 0.66)	‐0.25 (‐0.87, 0.6)	NA	52.3
bvNMA 1d	‐0.75 (‐0.9, ‐0.51)				NA	39.8
bvNMA 2a	‐0.44 (‐0.85, 0.13)	‐0.78 (‐0.95, ‐0.47)	‐0.03 (‐0.89, 0.88)	‐0.28 (‐0.94, 0.67)	‐0.33(‐0.92, 0.56)	39.5
bvNMA 2b	‐0.62 (‐0.9, ‐0.09)	‐0.78 (‐0.94, ‐0.48)	‐0.3 (‐0.91, 0.66)	‐0.28 (‐0.89, 0.59)	‐0.33 (‐0.92, 0.57)	40.0
bvNMA 2c	‐0.6 (‐0.89, ‐0.13)	‐0.73 (‐0.93, ‐0.42)	‐0.25 (‐0.89, 0.68)	‐0.25 (‐0.87, 0.59)	‐0.33 (‐0.91, 0.56)	56.6
bvNMA 2d	‐0.75 (‐0.9, ‐0.5)				‐0.36 (‐0.92, 0.52)	38.4
intercepts						
BRMA	‐0.05 (‐0.16, 0.06)					
bvNMA 1a	‐0.28 (‐0.42, ‐0.13)	‐0.03 (‐0.18, 0.12)	0.05 (‐1.26, 0.96)	0.12 (‐0.32, 0.53)		
bvNMA 1b	‐0.25 (‐0.39, ‐0.11)	‐0.03 (‐0.17, 0.11)	0.13 (‐0.15, 0.42)	0.11 (‐0.25, 0.48)		
bvNMA 1c	‐0.25 (‐0.38, ‐0.12)	‐0.04 (‐0.18, 0.1)	0.12 (‐0.15, 0.42)	0.11 (‐0.13, 0.37)		
bvNMA 1d	‐0.22 (‐0.32, ‐0.11)	‐0.04 (‐0.16, 0.08)	0.18 (0.07, 0.29)	0.16 (‐0.03, 0.35)		
bvNMA 2a	‐0.26 (‐0.4, ‐0.11)	‐0.03 (‐0.18, 0.11)	0.07 (‐0.8, 0.95)	0.13 (‐0.16, 0.44)		
bvNMA 2b	‐0.23 (‐0.37, ‐0.09)	‐0.03 (‐0.17, 0.11)	0.12 (‐0.16, 0.42)	0.12 (‐0.14, 0.39)		
bvNMA 2c	‐0.23 (‐0.36, ‐0.1)	‐0.04 (‐0.18, 0.09)	0.11 (‐0.17, 0.41)	0.12 (‐0.09, 0.35)		
bvNMA 2d	‐0.2 (‐0.31, ‐0.1)	‐0.04 (‐0.16, 0.08)	0.16 (0.06, 0.27)	0.16 (‐0.01, 0.33)		

Note: A, chemotherapy alone; B, antiangiogenic treatments targeting vascular endothelial growth factor (anti‐VEGF) therapies + chemotherapy; C, epidermal growth factor receptor inhibitor (EGFRi) + chemotherapy; D, EGFRi + anti‐VEGF therapies + chemotherapy

Abbreviations: BRMA, bivariate random effects meta‐analysis; bvNMA, bivariate network meta‐analysis.

Table 3 shows statistics for model comparison, introduced in Section 4.3.1, for each treatment contrast where at least four studies were available and across all the studies. For example, for the treatment contrast AB, the use of bvNMA improved the precision of the predictions in terms of the point estimate, reducing the bias from 0.23 (obtained from BRMA) to between 0.16 and 0.19. All bvNMA methods gave reduced predicted intervals for the treatment effect on PFS (from the effect on TR) compared to those obtained from BRMA, between 12.2% and 17.1%. For contrasts AC and BC, model 1a did not contribute to reduced uncertainty of predictions, but additional assumptions of second‐order consistency (models 1b and 2b) and additional borrowing of strength (models 1c and 2c) or assumption of homogeneity of the correlations and heterogeneity parameters (models 1d and 2d) led to improved precision. The three observations in contrast AC seen in the bottom right corner of the scatter plot of Figure 1 could be considered as potentially influential observations. The impact of these observations on the correlations was assessed by sensitivity analysis. Results from the sensitivity analysis of the data with these observations removed are presented in the Supplementary Materials. For the treatment contrast BD, both sets of models 1a to 1b and 2a to 2b resulted in inflated predicted intervals. The final part of Table 3 lists the average statistics across all treatments.

**Table 3:** Comparison of models based on advanced colorectal cancer data presented by treatment contrast for contrasts AB, AC, BC, and BD, and overall for all data (for models 1a to 1d, the statistics were obtained for all the studies excluding those in contrasts containing a single study)

	**p**_**overlap**_	|**m**_**obs**_ − **m**_**pred**_|	**w**_**pred**_/**w**_**obs**_	**π**	**%red.**
AB					
BRMA	0.9	0.23	2.25	0.44	0
bvNMA 1a	0.9	0.19	1.87	0.53	16.61
bvNMA 1b	0.91	0.17	1.96	0.52	12.28
bvNMA 1c	0.9	0.18	1.84	0.54	17.09
bvNMA 1d	0.91	0.16	1.88	0.53	14.55
bvNMA 2a	0.9	0.19	1.89	0.52	15.71
bvNMA 2b	0.91	0.18	1.97	0.51	12.17
bvNMA 2c	0.9	0.18	1.85	0.53	16.88
bvNMA 2d	0.9	0.16	1.87	0.53	15.16
AC					
BRMA	0.92	0.24	1.93	0.49	0
bvNMA 1a	0.94	0.24	1.92	0.51	0.24
bvNMA 1b	0.91	0.24	1.8	0.52	6.09
bvNMA 1c	0.88	0.25	1.7	0.53	11.06
bvNMA 1d	0.86	0.25	1.65	0.53	13.71
bvNMA 2a	0.94	0.24	1.92	0.51	0.1
bvNMA 2b	0.91	0.24	1.79	0.52	6.51
bvNMA 2c	0.87	0.25	1.69	0.53	11.59
bvNMA 2d	0.85	0.25	1.64	0.53	14.42
BC					
BRMA	0.97	0.17	2.3	0.46	0
bvNMA 1a	0.99	0.09	3.1	0.4	‐28.54
bvNMA 1b	0.96	0.09	2.13	0.5	8.07
bvNMA 1c	0.95	0.09	1.84	0.55	19.53
bvNMA 1d	0.95	0.1	1.94	0.52	14.91
bvNMA 2a	0.99	0.08	2.98	0.42	‐23.21
bvNMA 2b	0.96	0.08	2.08	0.51	10.21
bvNMA 2c	0.95	0.08	1.75	0.58	23.11
bvNMA 2d	0.96	0.09	1.93	0.53	15.16
BD					
BRMA	0.82	0.3	1.89	0.45	0
bvNMA 1a	1	0.23	4.34	0.25	‐128.76
bvNMA 1b	1	0.23	3.7	0.29	‐94.61
bvNMA 1c	0.84	0.25	1.74	0.51	7.59
bvNMA 1d	1	0.14	1.78	0.58	5.29
bvNMA 2a	1	0.21	3.46	0.31	‐83.09
bvNMA 2c	0.83	0.23	1.64	0.52	12.2
bvNMA 2d	1	0.13	1.73	0.6	8.07
All					
BRMA	0.91	0.24	2.02	0.48	0
bvNMA 1a	0.9	0.19	2.36	0.45	‐23.74
bvNMA 1b	0.89	0.19	2.2	0.46	‐16.79
bvNMA 1c	0.86	0.2	1.7	0.51	11.27
bvNMA 1d	0.86	0.18	1.69	0.51	11.67
bvNMA 2a	0.94	0.2	2.34	0.47	‐20.36
bvNMA 2b	0.93	0.2	2.14	0.49	‐10.02
bvNMA 2c	0.89	0.2	1.73	0.54	12.75
bvNMA 2d	0.9	0.19	1.73	0.54	12.76

Abbreviations: BRMA, bivariate random effects meta‐analysis; bvNMA, bivariate network meta‐analysis.

## 6 SIMULATION STUDY

### 6.1 Methods

**Figure 3:**
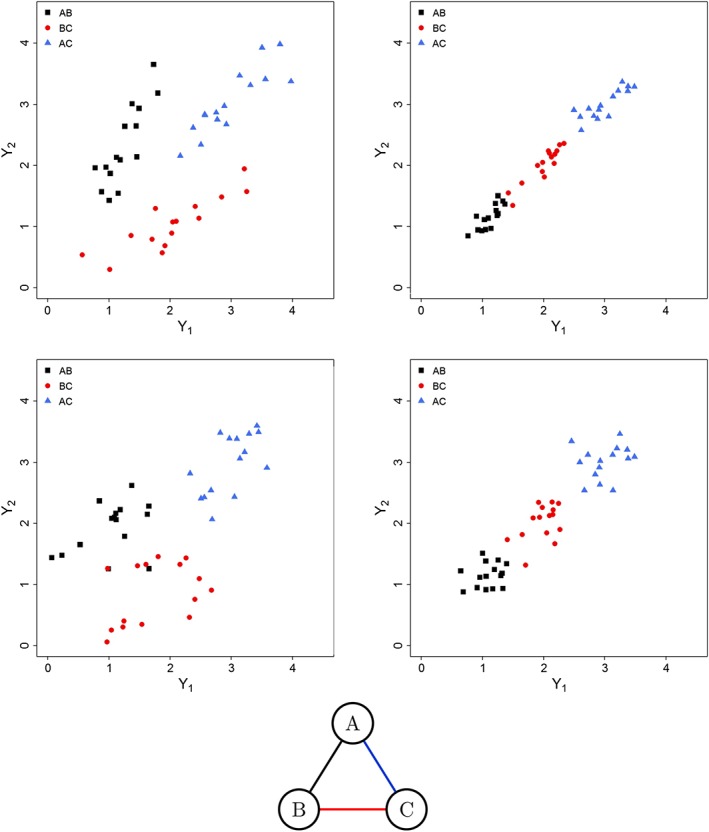
Scatter plots of the artificial data simulated under scenario 1 (top left), scenario 2 (top right), scenario 3 (bottom left), and scenario 4 (bottom right) and network diagram corresponding to the structure of data for both scenarios [Colour figure can be viewed at wileyonlinelibrary.com]

To compare the performance of the methods, data were simulated under four different assumptions. Specifically, we simulated data where the surrogate pattern across all studies and treatments differed from the patterns within treatment contrasts. The treatment effects on two outcomes were simulated from the bvNMA model 1a. Four sets of network data were generated, each consisting of 45 studies, three treatments (A, B, and C), and three treatment contrasts (AB, BC, and AC) with 15 studies per contrast, under different scenarios. A representative data set from each scenario is illustrated in Figure 3.

**Scenario 1** shows weak study‐level surrogacy when ignoring treatment contrasts but strong study‐level surrogacy within each treatment contrast. It uses the following parameters: **d**_*AB*_ = (1,2), **d**_*BC*_ = (2,1), **d**_*AC*_ = (3,3); *σ*_*jAB*(*AC*,*BC*)*i*_∼*Unif*(0.15,0.25), j=1,2; *ρ*_*wABi*_ = *ρ*_*wBCi*_ = *ρ*_*wACi*_ = 0.6; *τ*_1*AB*_ = 0.3, *τ*_1*BC*_ = *τ*_1*AC*_ = 0.6, *τ*_2*BC*_ = 0.3, and *τ*_2*AB*_ = *τ*_2*AC*_ = 0.6; *ρ*_*AB*_ = *ρ*_*AC*_ = *ρ*_*BC*_ = 0.9.

**Scenario 2** shows strong study‐level surrogacy both when ignoring treatment contrasts and within each treatment contrast, with the following parameters: **d**_*AB*_ = (1,1), **d**_*BC*_ = (2,2), **d**_*AC*_ = (3,3); *σ*_*jAB*(*AC*,*BC*)*i*_∼*Unif*(0.05,0.15), j = 1,2; *ρ*_*wABi*_ = *ρ*_*wBCi*_ = *ρ*_*wACi*_ = 0.98; *τ*_1*AB*_ = 0.2, *τ*_1*BC*_ = 0.25, *τ*_1*AC*_ = 0.3, *τ*_2*AB*_ = 0.3, *τ*_2*BC*_ = 0.25, and *τ*_2*AC*_ = 0.2; *ρ*_*AB*_ = *ρ*_*AC*_ = *ρ*_*BC*_ = 0.9.

**Scenario 3** shows no study‐level surrogacy both when ignoring treatment contrasts and within each treatment contrast, with the following parameters: **d**_*AB*_ = (1,2), **d**_*BC*_ = (2,1), and **d**_*AC*_ = (3,3); *σ*_*jAB*(*AC*,*BC*)*i*_∼*Unif*(0.15,0.25), j = 1,2; *ρ*_*wABi*_ = *ρ*_*wBCi*_ = *ρ*_*wACi*_ = 0.6; *τ*_1*AB*_ = *τ*_1*BC*_ = *τ*_1*AC*_ = *τ*_2*BC*_ = *τ*_2*AB*_ = *τ*_2*AC*_ = 0.4; *ρ*_*AB*_ = *ρ*_*AC*_ = *ρ*_*BC*_ = 0.25.

**Scenario 4** shows strong study‐level surrogacy when ignoring treatment contrasts but no study‐level surrogate relationship within each treatment contrast. It uses the following parameters: **d**_*AB*_ = (1,1), **d**_*BC*_ = (2,2), and **d**_*AC*_ = (3,3); *σ*_*jAB*(*AC*,*BC*)*i*_∼*Unif*(0.05,0.15), j = 1,2; *ρ*_*wABi*_ = *ρ*_*wBCi*_ = *ρ*_*wACi*_ = 0.6; *τ*_1*AB*_ = 0.2, *τ*_1*BC*_ = 0.25, *τ*_1*AC*_ = 0.3, *τ*_2*AB*_ = 0.3, *τ*_2*BC*_ = 0.25, and *τ*_2*AC*_ = 0.2; *ρ*_*AB*_ = *ρ*_*AC*_ = *ρ*_*BC*_ = 0.25.

The data were simulated from model 1a for simplicity, to allow for generating very clear and simple scenarios. Although by simulating from model 1a we do not explicitly assume second‐order consistency, this does not mean that the assumption is not satisfied for the simulated data and, in fact, the parameters we choose are such that the second‐order consistency is satisfied, which is shown in Section 10 of the Supplementary Materials.

For each scenario, 1000 data sets were simulated and all models were fitted to these data. Between‐studies correlations for each treatment contrast were monitored for their estimated values, coverage, root mean squared error (RMSE), and Monte Carlo error. Subsequently, another simulation study was conducted aiming to evaluate the methods when used for predicting the treatment effect on the final outcome from the effect measured on the surrogate endpoint. The same four scenarios were used and the predictions were made assuming that the first simulated study, for each contrast, in turn, was a new study (in a similar manner as in the cross‐validation procedure, as described in Section 4.3.1, but only for three studies in each simulated data set).

### 6.2 Results

Table 4 shows mean values of estimated between‐studies correlations, corresponding mean width of the CrI, coverage, and RMSE for each scenario and all NMA models. Only mean of the correlation and mean width of CrI are shown for BRMA and NMA models 1d and 2d as these methods do not estimate individual correlations for each contrast, and hence, the coverage and the RMSE were not applicable. Table 5 shows results from the simulation study evaluating the predictions of the treatment effect on the final outcome from the treatment effect on the surrogate endpoint. The values presented are the coverage and the RMSE for the true effects on the final outcome *μ*_2*kli*_ for study *i*, which is the first study out of 15 studies simulated per treatment contrast (studies 1, 16, and 31). The last column for each contrast corresponds to the ratio between the width of the predicted interval from each model to the width of the interval obtained from BRMA.

**Table 4:** Results of the simulation study showing the between‐studies correlations ρ_kl_ for each treatment contrast (for bivariate random effects meta‐analysis (BRMA) and models 1d and 2d only a single overall correlation available), the corresponding widths of the credible intervals (wCrIs), coverage, and root mean squared error (RMSE). 99% of simulations resulted in Monte Carlo error<0.02 in scenario 1, 99.7% in scenario 2, more than 99.75% in scenario 3 and 100% in scenario 4

	AB			BC			AC		
	mean			mean			mean		
model	***ρ***_***kl***_ / wCrI	coverage	RMSE	***ρ***_***kl***_ / wCrI	coverage	RMSE	***ρ***_***kl***_ / wCrI	coverage	RMSE
*scenario 1: true correlations ρ_kl_ = 0.9*									
BRMA				0.55 / 0.42					
bvNMA 1a	0.73 / 0.70	0.93	0.23	0.74 / 0.68	0.94	0.22	0.82 / 0.43	0.96	0.13
bvNMA 1b	0.77 / 0.58	0.95	0.19	0.72 / 0.62	0.88	0.22	0.84 / 0.40	0.98	0.11
bvNMA 1c	0.74 / 0.61	0.92	0.21	0.69 / 0.65	0.80	0.25	0.82 / 0.42	0.94	0.13
bvNMA 1d				0.77 / 0.30					
bvNMA 2a	0.73 / 0.70	0.93	0.23	0.74 / 0.68	0.94	0.22	0.82 / 0.44	0.96	0.13
bvNMA 2b	0.77 / 0.57	0.95	0.19	0.72 / 0.61	0.88	0.22	0.84 / 0.38	0.97	0.11
bvNMA 2c	0.74 / 0.61	0.92	0.21	0.69 / 0.65	0.80	0.25	0.82 / 0.42	0.94	0.13
bvNMA 1d				0.77 / 0.30					
*scenario 2: true correlations ρ_kl_ = 0.9*									
BRMA				0.99 / 0.02					
bvNMA 1a	0.80 / 0.47	0.94	0.15	0.80 / 0.46	0.92	0.16	0.80 / 0.47	0.93	0.16
bvNMA 1b	0.82 / 0.40	0.95	0.13	0.80 / 0.47	0.94	0.16	0.81 / 0.41	0.93	0.14
bvNMA 1c	0.79 / 0.44	0.88	0.15	0.76 / 0.51	0.84	0.19	0.79 / 0.44	0.88	0.16
bvNMA 1d				0.82 / 0.22					
bvNMA 2a	0.80 / 0.47	0.94	0.15	0.80 / 0.46	0.92	0.16	0.80 / 0.47	0.93	0.16
bvNMA 2b	0.82 / 0.40	0.95	0.13	0.80 / 0.47	0.94	0.16	0.81 / 0.41	0.93	0.14
bvNMA 2c	0.79 / 0.44	0.89	0.15	0.76 / 0.51	0.85	0.19	0.79 / 0.43	0.88	0.16
bvNMA 2d				0.82 / 0.22					
*scenario 3: true correlations ρ_kl_ = 0.25*									
BRMA				0.42 / 0.49					
bvNMA 1a	0.16 / 1.08	0.98	0.27	0.16 / 1.07	0.97	0.27	0.16 / 1.07	0.97	0.27
bvNMA 1b	0.19 / 1.04	0.98	0.26	0.16 / 1.01	0.98	0.24	0.19 / 1.03	0.98	0.25
bvNMA 1c	0.15 / 1.01	0.97	0.27	0.14 / 0.97	0.96	0.25	0.15 / 1.00	0.96	0.27
bvNMA 1d				0.22 / 0.66					
bvNMA 2a	0.16 / 1.08	0.98	0.27	0.16 / 1.07	0.97	0.27	0.16 / 1.07	0.97	0.27
bvNMA 2b	0.19 / 1.04	0.98	0.26	0.18 / 1.01	0.98	0.24	0.19 / 1.03	0.98	0.25
bvNMA 2c	0.15 / 1.01	0.97	0.27	0.14 / 0.97	0.96	0.25	0.15 / 1.01	0.96	0.27
bvNMA 2d				0.22 / 0.66					
*scenario 4: true correlations ρ_kl_ = 0.25*									
BRMA				0.92 / 0.09					
bvNMA 1a	0.17 / 1.02	0.98	0.25	0.17 / 1.00	0.97	0.25	0.17 / 1.02	0.96	0.26
bvNMA 1b	0.19 / 0.98	0.98	0.24	0.18 / 0.98	0.98	0.23	0.19 / 0.98	0.97	0.25
bvNMA 1c	0.17 / 0.95	0.96	0.25	0.15 / 0.92	0.96	0.23	0.16 / 0.95	0.95	0.25
bvNMA 1d				0.21 / 0.62					
bvNMA 2a	0.17 / 1.02	0.98	0.25	0.17 / 1.00	0.97	0.25	0.17 / 1.02	0.96	0.26
bvNMA 2b	0.19 / 0.98	0.98	0.24	0.18 / 0.98	0.98	0.23	0.19 / 0.98	0.97	0.25
bvNMA 2c	0.17 / 0.95	0.96	0.25	0.15 / 0.92	0.96	0.23	0.16 / 0.95	0.95	0.25
bvNMA 2d				0.21 / 0.62					

Abbreviations: bvNMA, bivariate network meta‐analysis.

**Table 5:** Results of the simulation study showing the performance of the models in terms of predicting the treatment effect on the final outcomes from the effect measured on surrogate endpoint in a new study. Presented values are coverage and root mean squared error (RMSE) for the true effects on the final outcome and the CrI width ratio (wCrIr); the ratio between the width of the predicted interval from each model to the width of the interval obtained from bivariate random effects meta‐analysis (BRMA)

	AB			BC			AC		
model	coverage	RMSE	wCrIr	coverage	RMSE	wCrIr	coverage	RMSE	wCrIr
*scenario 1*									
BRMA	0.99	0.72		0.99	0.72		1.00	1.10	
bvNMA 1a	0.99	0.41	0.57	0.98	0.42	0.57	0.98	0.19	0.27
bvNMA 1b	0.98	0.41	0.51	0.97	0.42	0.51	1.0	0.18	0.29
bvNMA 1c	0.97	0.42	0.48	0.96	0.43	0.48	0.99	0.19	0.27
bvNMA 1d	0.95	0.44	0.43	0.95	0.45	0.43	0.98	0.26	0.45
*scenario 2*									
BRMA	0.93	0.19		0.95	0.18		0.98	0.15	
bvNMA 1a	0.98	0.19	1.15	0.97	0.18	1.16	0.98	0.15	0.92
bvNMA 1b	0.96	0.19	1.04	0.96	0.18	1.05	0.99	0.15	0.96
bvNMA 1c	0.96	0.20	1.01	0.95	0.19	1.01	0.98	0.15	0.92
bvNMA 1d	0.93	0.20	0.90	0.94	0.19	0.90	0.99	0.15	0.92
*scenario 3*									
BRMA	1.0	0.57		1.0	0.59		0.95	1.07	
bvNMA 1a	0.97	0.40	0.51	0.97	0.42	0.51	0.98	0.40	0.52
bvNMA 1b	0.97	0.40	0.50	0.98	0.42	0.50	0.98	0.39	0.52
bvNMA 1c	0.96	0.40	0.45	0.96	0.42	0.45	0.97	0.39	0.47
bvNMA 1d	0.98	0.39	0.47	0.97	0.41	0.47	0.99	0.39	0.48
*scenario 4*									
BRMA	0.95	0.35		0.95	0.35		0.97	0.30	
bvNMA 1a	0.97	0.31	0.96	0.98	0.31	0.96	0.98	0.25	0.83
bvNMA 1b	0.96	0.30	0.88	0.97	0.31	0.89	0.98	0.25	0.83
bvNMA 1c	0.95	0.30	0.82	0.96	0.31	0.83	0.97	0.25	0.76
bvNMA 1d	0.95	0.30	0.75	0.95	0.31	0.75	0.97	0.25	0.76

Abbreviations: bvNMA, bivariate network meta‐analysis.

#### 6.2.1 Scenario 1

As shown in the top part of Table 4, the between‐studies correlation obtained from BRMA (across all studies) was not very high, on average 0.55 (mean width of 95% CrI (wCrI): 0.42). Bivariate NMA with the covariance matrix varying across treatment contrasts modelled the data in more detail and revealed stronger correlation between the treatment effects on the two outcomes within the treatment contrasts, on average 0.73 (0.70) for treatment contrast AB, 0.74 (0.68) for BC, and 0.82 (0.43) for AC when using model 1a. Placing additional constraints on the covariance matrix by assuming second‐order consistency in models 1b and 1c reduced uncertainty around the correlations. Model 1d resulted in common correlations obtained with the highest precision, resulting from the assumption of homogeneity of the correlations and hence much larger data contributing to these estimates. However, model 1d did not take into account the differences in the between‐studies variances across the treatment contrasts, in contrast to models 1a to 1c. Similar patterns can be observed for models 2a to 2d.

The coverage was high except for models 1b, 1c, 2b, and 2c for the correlation corresponding to the contrast BC. This was likely due to the correlation being underestimated (more so than by models 1a and 2a) but obtained with higher precision, compared to models 1a and 2a where the coverage was high, due to the second‐order consistency assumption enforcing additional constraints on the covariances.

There may be a number of potential mechanisms for the reduced coverage of the correlation (seen in models 1b, 1c, 2b, and 2c and in particular for contrast BC). The between‐studies correlations are usually underestimated because of a relatively small sample of studies in meta‐analysis. Assuming second‐order consistency, in addition to random effects and the first‐order consistency assumption, is likely to result in further borrowing of information. This may lead to increased precision of the estimated correlations and/or more shrinkage of the true effects (towards the mean effects) on both outcomes, which may reduce the mean value of the correlation. Change in coverage will be a result of a balance between bias and precision. For example, for contrasts AB and AC, model 1b gave more precise correlations, which could result in reduced coverage, but the mean correlations were closer to the true values resulting in increased coverage compared to model 1a. However, for contrast BC, the correlation was obtained with higher precision and the point estimate moved away from the true value increasing the bias and thus led to reduced coverage. It is difficult to compare the results from models 1a and 1b directly because they use different sets of prior distributions for the correlations. Moreover, the choice of the ordering of the treatment contrasts may impact on the prior distributions in model 1b, which used a Cholesky decomposition.

The top part of Table 5 shows results from the simulation study evaluating the predictions of the treatment effect on the final outcome from the treatment effect on the surrogate endpoint in scenario 1. Large coverage for each contrast indicated perhaps large uncertainty. However, the bias expressed as RMSE was reduced when using NMA models compared to BRMA. The uncertainty was also largely reduced when using NMA models compared to BRMA, between 43% and 73%.

#### 6.2.2 Scenario 2

The second section of Table 4 shows the between‐studies correlations for the data simulated under scenario 2. The overall mean correlation obtained from BRMA is high, ie, 0.99 (wCrI: 0.02). Bivariate NMA with the variance‐covariance matrix varying across treatment contrasts resulted in high correlations within each treatment contrast, but obtained with much higher uncertainty compared to BRMA, due to fewer data points within each treatment contrast. The coverage was low for all correlations obtained from models 1c and 2c, likely also due to the correlation being underestimated to a higher degree compared to models 1a, 1b, 2a, and 2b.

The second section of Table 5 shows results from the simulation study evaluating the predictions. There was not much improvement in predictions when using NMA models, as BRMA gave precise predictions already, due to the very strong surrogacy patterns across all data. The width of the predicted interval varied across models and treatment contrasts ranging between being inflated by up to 15% or reduced by up to 10%.

#### 6.2.3 Scenario 3

The third section of Table 4 shows the average between‐studies correlations for the data simulated under scenario 3, which indicate the lack of surrogate relationship at the study level overall (BRMA) where the mean correlation was 0.42 and within each treatment contrast (NMA models) where correlations were much smaller and obtained with large uncertainty. Coverage was high due to the large uncertainty around the estimates of the correlations. The results were comparable across models, perhaps with reduced uncertainty around the correlations when using models 1d and 2d.

The third section of Table 5 shows results of the simulation investigating predictions in scenario 3. A large improvement was noted in terms of reduced bias and uncertainty of predictions when using NMA models compared to BRMA where the predicted intervals were reduced by approximately 50%.

#### 6.2.4 Scenario 4

The bottom section of Table 4 shows the average between‐studies correlations for the data simulated under scenario 4. The mean correlation obtained from BRMA is very high, ie, 0.92 (wCrI: 0.09). However, as expected, the correlations within each contrast indicated no surrogate relationship at the study‐level within these contrasts. Similarly, as in scenario 3, the results were comparable across models, but with reduced uncertainty around the correlations when using models 1d and 2d.

As shown in the bottom part of Table 5, the improvement in predictions was less pronounced in scenario 4, but still noticeable, with the width of the CrI being reduced between 4% and 25%.

### 6.3 Discussion of the simulations

To help the reader understand better the heterogeneity patterns in each data scenario and their implications on the measures of surrogate relationships, we simulated and analysed single data sets under very similar scenarios and presented detailed results of these analyses in the Supplementary Materials. We presented there also an additional scenario, with a mixed surrogacy pattern (strong and weak) across treatment contrasts to illustrate better (compared to the illustrative example in aCRC) how the proposed methods differentiate between the surrogate relationships across treatment contrasts and hence allow for identifying treatment contrasts with high surrogacy patterns.

The aforementioned simulation study investigated data scenarios where a number of trials exist for each treatment contrast. This illustrates well the use of the bvNMA models to investigate the study‐level surrogacy within treatment contrasts and for making predictions of the treatment effect on the final outcome from the treatment effect on the surrogate endpoint in a new study investigating an existing treatment in the network but in a new population (eg, a different country). As discussed in Section 4.2, bvNMA models 2a to 2d can be used to make such predictions for a new treatment when no data are available yet for the effect of this treatment on the final outcome. We investigated this through an initial simulation study and, although such predictions can be made, eg, for treatment D as illustrated in Figure 2, these predictions are obtained with large uncertainty. Further investigation in larger networks led to not only the same conclusions but indicated that the predictions are likely to be comparable or more uncertain compared to those obtained from BRMA. Nevertheless, bvNMA models 2a to 2d are still valuable as they can be used to estimate the average effects of the new treatment on both outcomes relative to any treatment in the network, which is not achievable by BRMA and bvNMA models 1a to 1d can only estimate these effects on the surrogate endpoint (when no measurement on the final clinical outcome is available). This quality may be useful in a regulatory or health technology assessment decision‐making context, in particular at the early stages of the drug development process. Further research is required to investigate the properties, assumptions, and application of models 2a to 2d.

## 7 DISCUSSION

We have developed bivariate network meta‐analytic models for surrogate endpoint evaluation to allow for more detailed modelling of surrogate relationships at the study level (within each treatment contrast) and at the treatment level. This methodology can help to disentangle information about surrogate relationships in data scenarios where such relationships vary across treatment contrasts and are distinct in comparison with an association pattern across treatments. Two types of surrogacy have been described by the models, ie, study‐level surrogacy for a given treatment contrast and treatment‐level surrogacy. The models allow analysts to make predictions of the treatment effect on the final clinical outcome from the observed effect on a surrogate endpoint in a new study investigating the effectiveness of an existing treatment in a new setting or a new treatment.

There are some limitations to the models presented here. For simplicity, we focused on the models for data from two‐arm studies. The methods can be extended to model multi‐arm trial data in a similar manner as in Achana et al.^12^ Another limitation is related to the prior distribution for the ancillary variance‐covariance matrix in model 1b, introduced to allow for the second‐order consistency constraints. It was based on the Cholesky decomposition, which results in the prior distributions for the between‐studies correlations being dependent on the ordering of the treatments in the network. We carried out a sensitivity analysis by changing the ordering of treatments in the illustrative example in aCRC; the results remained very similar to those obtained from the main analysis. Alternative prior distributions could also be constructed for the covariance matrix, eg, using the separation strategy by spherical decomposition^10^, ^12^, ^17^ or an inverse Wishart prior distribution; however, a number of authors found that Wishart priors had an influential impact on the posterior distribution.^6^, ^10^, ^30^

The choice of the model parameterisation and a prior distribution can be difficult to make. The decision about the final model can be made based on DIC. In addition, plausibility of the assumptions of individual models should be considered. For example, when choosing between models 1a and 1b, plausibility of the second‐order consistency assumption needs to be verified. For the second‐order consistency assumption, we are requiring not only first order but also second‐order moments to “add up around loops” and so this stronger assumption could also be thought more plausible in situations where the trial populations are “especially” similar. In addition, to aid decisions about the model choice, the second‐order consistency assumption can also be checked by following a procedure similar to the one used in Section 10 of the Supplementary Materials.

Another sensitivity analysis was carried out to investigate the impact of potentially influential observations, which were detected by visually assessing the scatter plots. More formal approaches to detecting outlying observations are available, developed by Zhang et al^31^ and Viechtbauer el al,^32^ but were beyond the scope of this paper.

Scarcity of data may also present a problem in fitting the models. For estimation of the surrogate relationships within the treatment contrasts, a relatively large number of trials per treatment contrast is needed, whereas for the treatment‐level surrogacy, a relatively large number of treatments needs to be included in the network.

The models presented in this paper were developed to describe the surrogate relationships between the average effects at the study level and at the treatment level, but not at the individual level. Further work will be carried out to extend the methods to bivariate IPD NMA to incorporate the individual‐level surrogacy as in the works of Buyse et al^3^ and Burzykowski et al.^4^

We believe that the models have great potential for making improvements in the research area of surrogate endpoint evaluation. In our example in aCRC, the models allowed us to disentangle information on a relatively strong study‐level surrogate relationship between treatment effects on TR and PFS for the treatment contrast of EGFRi with chemotherapy vs. chemotherapy alone from a set of treatments with suboptimal overall surrogacy relationship. Moreover, in medical decision making, where multiple comparisons of new health technologies against different comparators play an important role, NMA is a valuable tool in obtaining average effects across all treatment contrasts in the network of treatments. In a similar way, our proposed methodology can be used to predict the effect of a new treatment on the final clinical outcome against any comparator in the network. In conclusion, we developed a new meta‐analytic method for surrogate endpoint evaluation that allows modelling of surrogate relationships in greater detail.

## Supporting information

SIM_8187‐Supp‐0001‐supplement 1 May 2019.pdf

## References

[1] Burzykowski T , Molenberghs G , Buyse M . The Evaluation of Surrogate Endpoints. New York, NY: Springer; 2006.

[2] Daniels MJ , Hughes MD . Meta‐analysis for the evaluation of potential surrogate markers. Statist Med. 1997;16(17):1965‐1982. 10.1002/(sici)1097-0258(19970915)16:17<1965::aid-sim630>3.0.co;2-m 9304767

[3] Buyse M , Molenberghs G , Burzykowski T , Renard D , Geys H . The validation of surrogate endpoints in meta‐analyses of randomized experiments. Biostatistics. 2000;1(1):49‐67. 12933525 10.1093/biostatistics/1.1.49

[4] Burzykowski T , Molenberghs G , Buyse M , Geys H , Renard D . Validation of surrogate end points in multiple randomized clinical trials with failure time end points. J Royal Stat Soc Ser C (Appl Stat). 2001;50(4):405‐422.

[5] Renfro LA , Shi Q , Sargent DJ , Carlin BP . Bayesian adjusted R2 for the meta‐analytic evaluation of surrogate time‐to‐event endpoints in clinical trials. Statist Med. 2012;31(8):743‐761. 10.1002/sim.4416 22161275

[6] Bujkiewicz S , Thompson JR , Spata E , Abrams KR . Uncertainty in the Bayesian meta‐analysis of normally distributed surrogate endpoints. Stat Methods Med Res. 2017;26(5):2287‐2318. 26271918 10.1177/0962280215597260 PMC5642004

[7] Bujkiewicz S , Thompson JR , Riley RD , Abrams KR . Bayesian meta‐analytical methods to incorporate multiple surrogate endpoints in drug development process. Statist Med. 2016;35(7):1063‐1089. 10.1002/sim.6776 PMC4950070 26530518

[8] Van Houwelingen HC , Arends LR , Stijnen T . Advanced methods in meta‐analysis: multivariate approach and meta‐regression. Statist Med. 2002;21(4):589‐624. 10.1002/sim.1040 11836738

[9] Riley RD , Abrams KR , Lambert PC , Sutton AJ , Thompson JR . An evaluation of bivariate random‐effects meta‐analysis for the joint synthesis of two correlated outcomes. Statist Med. 2007;26(1):78‐97. 10.1002/sim.2524 16526010

[10] Wei Y , Higgins J . Bayesian multivariate meta‐analysis with multiple outcomes. Statist Med. 2013;32(17):2911‐2934. 10.1002/sim.5745 23386217

[11] Bujkiewicz S , Thompson JR , Sutton AJ , et al. Multivariate meta‐analysis of mixed outcomes: a Bayesian approach. Statist Med. 2013;32(22):3926‐3943. 10.1002/sim.5831 PMC4015389 23630081

[12] Achana FA , Cooper NJ , Bujkiewicz S , et al. Network meta‐analysis of multiple outcome measures accounting for borrowing of information across outcomes. BMC Med Res Methodol. 2014;14(1):92. 25047164 10.1186/1471-2288-14-92 PMC4142066

[13] Efthimiou O , Mavridis D , Cipriani A , Leucht S , Bagos P , Salanti G . An approach for modelling multiple correlated outcomes in a network of interventions using odds ratios. Statist Med. 2014;33(13):2275‐2287. 10.1002/sim.6117 24918246

[14] Hong H , Fu H , Price KL , Carlin BP . Incorporation of individual‐patient data in network meta‐analysis for multiple continuous endpoints, with application to diabetes treatment. Statist Med. 2015;34(20):2794‐2819. 10.1002/sim.6519 25924975

[15] Hong H , Chu H , Zhang J , Carlin BP . A Bayesian missing data framework for generalized multiple outcome mixed treatment comparisons. Res Synth Methods. 2016;7(1):6‐22. 26536149 10.1002/jrsm.1153 PMC4779385

[16] Jackson D , Bujkiewicz S , Law M , Riley RD , White IR . A matrix‐based method of moments for fitting multivariate network meta‐analysis models with multiple outcomes and random inconsistency effects. Biometrics. 2017;74(2):548‐556 28806485 10.1111/biom.12762 PMC6038911

[17] Lu GB , Ades A . Modeling between‐trial variance structure in mixed treatment comparisons. Biostatistics. 2009;10(4):792‐805. 19687150 10.1093/biostatistics/kxp032

[18] Wagner ADADW , Arnold D , Grothey AAG , Haerting J , Unverzagt S . Anti‐angiogenic therapies for metastatic colorectal cancer. Cochrane Database Syst Rev. 2009;3:1‐75. 10.1002/14651858.CD005392.pub3 PMC12227179 19588372

[19] Chan DLH , Segelov E , Wong RSH , et al. Epidermal growth factor receptor (EGFR) inhibitors for metastatic colorectal cancer. Cochrane Database Syst Rev. 2017;6:1‐175. 10.1002/14651858.CD007047.pub2 PMC6481896 28654140

[20] Mocellin S , Baretta Z , Figuls MR , et al. Second‐line systemic therapy for metastatic colorectal cancer. Cochrane Database Syst Rev. 2017;1:1‐121. 10.1002/14651858.CD006875.pub3 PMC6464923 28128439

[21] Kumachev A , Yan M , Berry S , et al. A systematic review and network meta‐analysis of biologic agents in the first line setting for advanced colorectal cancer. PloS one. 2015;10(10):e0140187. 26474403 10.1371/journal.pone.0140187 PMC4608731

[22] Elia EG , Städler N , Ciani O , Taylor RS , Bujkiewicz S . Combining tumour response and progression free survival as surrogate endpoints for overall survival in advanced colorectal cancer. 2018. arXiv e‐prints. arXiv:1809.02935. Accessed December 7, 2018. 10.1016/j.canep.2019.101665 31911395

[23] Riley RD , Price MJ , Jackson D , et al. Multivariate meta‐analysis using individual participant data. Res Synth Methods. 2015;6(2):157‐174. 26099484 10.1002/jrsm.1129 PMC4847645

[24] Wei Y , Higgins JPT . Estimating within‐study covariances in multivariate meta‐analysis with multiple outcomes. Statist Med. 2013;32(7):1191‐1205. 10.1002/sim.5679 PMC3618374 23208849

[25] Burke DL , Bujkiewicz S , Riley RD . Bayesian bivariate meta‐analysis of correlated effects: impact of the prior distributions on the between‐study correlation, borrowing of strength, and joint inferences. Stat Methods Med Res. 2018;27(2):428‐450. 26988929 10.1177/0962280216631361 PMC5810917

[26] Lu G , Ades AE . Combination of direct and indirect evidence in mixed treatment comparisons. Statist Med. 2004;23(20):3105‐3124. 10.1002/sim.1875 15449338

[27] Lassere MN , Johnson KR , Schiff M , Rees D . Is blood pressure reduction a valid surrogate endpoint for stroke prevention? an analysis incorporating a systematic review of randomised controlled trials, a by‐trial weighted errors‐in‐variables regression, the surrogate threshold effect (STE) and the biomarker‐surrogacy (BioSurrogate) evaluation schema (BSES). BMC Med Res Methodol. 2012;12(1):27. 22409774 10.1186/1471-2288-12-27 PMC3388460

[28] Institute for Quality and Efficiency in Health Care . Validity of surrogate endpoints in oncology. Executive summary of rapid report a10‐05, version 1.1. 2011 https://www.ncbi.nlm.nih.gov/books/nbk198799/. Accessed December 7, 2018. 24783303

[29] Alonso A , Bigirumurame T , Burzykowski T , et al. Applied Surrogate Endpoint Evaluation Methods with SAS and R. Boca Raton, FL: CRC Press; 2016.

[30] Daniels MJ , Pourahmadi M . Bayesian analysis of covariance matrices and dynamic models for longitudinal data. Biometrika. 2002;89(3):553‐566.

[31] Zhang J , Fu H , Carlin BP . Detecting outlying trials in network meta‐analysis. Statist Med. 2015;34(19):2695‐2707. 10.1002/sim.6509 PMC4496319 25851533

[32] Viechtbauer W , Cheung MWL . Outlier and influence diagnostics for meta‐analysis. Res Synth Methods. 2010;1(2):112‐125. 26061377 10.1002/jrsm.11

