# Supplementary material for: Bivariate network meta‐analysis for surrogate endpoint evaluation
Source: Stat Med. 2019 May 26;38(18):3322–41. doi: 10.1002/sim.8187 (PMC6618064; doi:10.1002/sim.8187)
Supplement: Supplementary file 1 — SIM_8187‐Supp‐0001‐supplement 1 May 2019.pdf [file SIM-38-3322-s001.pdf]

Received: 00 Month 0000 | Revised: 00 Month 0000 | Accepted: 00 Month 0000

DOI: xxx/xxxx

RESEARCH ARTICLE

Supplementary materials to: Bivariate network meta-analysis for surrogate endpoint evaluation

Sylwia Bujkiewicz*[1] | Dan Jackson[2] | John R Thompson[3] | Rebecca M Turner[4] | Nicolas Städler[5] | Keith R Abrams[1] | Ian R White[4]

[1]Biostatistics Research Group, Department of Health Sciences, University of Leicester U.K.
[2]Statistical Innovation Group, Astrazeneca U.K.
[3]Genetic Epidemiology Group, Department of Health Sciences, University of Leicester U.K.
[4]MRC Clinical Trials Unit, University College London U.K.
[5]Roche Innovation Center, F. Hoffmann-La Roche Ltd, Basel Switzerland

Correspondence
*Sylwia Bujkiewicz, Email: sylwia.bujkiewicz@le.ac.uk

Content of the supplement

Additional information about the data, models and results are presented in the supplement in the following order. Section 1 lists the data for the illustrative example in advanced colorectal cancer (aCRC). Section 2 describes the beta distribution based prior for the between-studies correlations. Section 3 lists all details of model 1b with the derivation of the second order consistency conditions, construction of prior distributions and WinBUGS code. The relationship between NMA models and the standard surrogate model is discussed in Section 4. Additional discussion of surrogacy criteria can be found in Section 5.
Additional results for the illustrative example in aCRC, such as correlations for the treatment contrasts where only a small number of studies were available as well as all heterogeneity parameters and the average effects, are presented in Section 6. Results of a sensitivity analysis removing potentially influential observations are reported in Section 7. Additional analysis of the simulated data sets can be found in Sections 8 for four scenarios similar to those described in the main manuscript and in Section 9 which introduces additional scenario with mixed surrogacy patterns across the treatment contrasts. In Section 10 we discuss the second order consistency assumption for data generated for the simulation study.

1 | DATA FOR ILLUSTRATIVE EXAMPLE IN ACRC

Tables 1 and 2 list the data for the illustrative example in advanced colorectal cancer (ACRC), introduced in Section 2 of the main manuscript. Three studies, which gave very large odds ratios (ORs) for the tumour response (TR) (marked by * in Table 1) were removed in the sensitivity analysis. Results of the sensitivity analysis are presented below in Section 7.

The data were modelled on the log HR scale for progression-free survival and on the log OR scale for tumour response. The within-study correlation between log OR for TR and log HR for PFS was obtained by Elia et al (2018) by bootstrapping method, from individual participant data of a RCT (reported by Hurwitz et al [1]) comparing Bevacizumab (anti-VEGF) with chemotherapy vs. chemotherapy alone. The within-study correlation, equal -0.433, was assumed the same across treatments.

study	log HR PFS	SE(log HR PFS)	R_e	N_e	R_c	N_c
anti-VEGF + chemo vs. chemo						
Guan (ARTIST)	-0.821	0.25	49	139	11	64
Hurwitz2004	-0.6162	0.0977	180	402	143	411
Passardi 2015 (ITACA)	-0.139	0.111	89	176	97	194
Saltz2008 (NO16966)	-0.1863	0.0618	328	699	344	701
Saunders 2013 (AVEX)	-0.635	0.193	27	140	14	140
Tebbutt 2010 (MAX)	-0.478	0.119	56	147	43	142
Borner 2008	-0.1819	0.2608	28	37	28	37
Sobrero 2008	-0.3682	0.0565	106	648	27	650
Bennuona 2013	-0.3857	0.0812	22	404	16	406
Cao 2015	-0.3425	0.1589	31	65	22	77
Giantonio2007	-0.4943	0.093	65	237	25	215
Masi 2015	-0.3567	0.1517	21	92	17	92
Hecht 2007	-0.1165	0.0709	269	562	274	560
Kabbinavar2003	-0.6162	0.2822	22	68	6	36
Kabbinavar2005 (AVF2192)	-0.6931	0.1949	27	104	16	105
EGFRi+chemo vs chemo						
Adams 2011 (COIN)	-0.0426	0.0795	232	363	209	367
Bokemeyer 2009 (OPUS)	-0.5682	0.2106	37	61	27	73
Douillard 2010 (PRIME)	-0.223	0.0982	177	322	157	327
Tveit 2012 (NORDIC-VII)	0.0679	0.1549	35	72	23	58
Van Cutsem 2009 (CRYSTAL)	-0.3631	0.1124	181	316	139	350
Ye 2013	-0.5154	0.1919	40	70	20	68
Ciardello 2016 CAPRI-GOIM	-0.2157	0.1679	16	74	10	79
Passardi 2015 ITACA	-0.4507	0.3057	7	24	4	24
Peeters 2010	-0.3165	0.1077	104	297	29	285
Seymour 2013 PICCOLO	-0.2488	0.1008	79	230	27	230
Amado 2008	-0.8032	0.1406	21	124	0	103
Karapetis 2008 CO17	-0.8743	0.1682	15	117	0	113
Bokemeyer 2009 (OPUS)	0.5422	0.2262	17	52	23	47
Douillard 2010 (PRIME)	0.2608	0.1131	88	221	88	219
Tveit 2012 (NORDIC-VII)	-0.3318	0.1844	35	72	23	58
Van Cutsem 2009 (CRYSTAL)	0.1572	0.1414	67	214	66	183
Peeters 2010	-0.1637	0.1132	30	232	33	237
Amado 2008*	-0.0036	0.1587	0	84	0	100
Karapetis 2008 CO17*	-0.0073	0.1568	1	81	0	83
Bokemeyer 2009 (OPUS)	-0.6351	0.344	22	38	14	49
Van Cutsem 2009 (CRYSTAL)	-0.583	0.1574	118	178	73	189
Peeters 2010	-0.3552	0.1331	83	204	21	207
Amado 2008*	-1.0201	0.1868	12	73	0	63
Santoro 2008	-0.1379	0.119	23	51	23	48

* studies removed for sensitivity analysis

TABLE 1 Data for the illustrative example in advanced colorectal cancer. R_e and N_e denote the number of responders and the total number of patients in the experimental arm and R_c and N_c denote these numbers in the control arm.

study	log HR PFS	SE(log HR PFS)	R_e	N_e	R_c	N_c
EGFRi+chemo vs anti-VEGF+chemo						
Hecht (SPIRITT)	0.0099	0.2018	28	87	16	83
Heinemann (FIRE-3)	0.0516	0.0916	184	297	171	295
Shwartzberg (PEAK)	-0.1369	0.1499	82	142	76	143
Venook (CALGB 80405)	0.0313	0.0641	381	578	319	559
EGFRi+anti-VEGF+chemo vs anti-VEGF+chemo						
Hecht (PACCE)	0.3211	0.1246	131	258	142	261
Passardi 2015 (ITACA)	0.2705	0.278	4	28	9	28
Tol (CAIRO2)	0.1731	0.1253	97	158	78	156
Tournigand (DREAM)	-0.2028	0.1085	48	213	24	208
anti-IGF1R+chemo vs chemo						
Cohn 2013	0.001	0.2554	4	51	1	49
EGRFi+anti-VEGF+chemo vs chemo						
Liu 2015	-0.4308	0.221	12	27	10	34
anti-IgG2+EGFRi+chemo vs EGFRi+chemo						
Elez	0.1222	0.1896	27	73	26	72
Elez	0.1044	0.1882	25	73	26	72

TABLE 2 Data for the illustrative example in advanced colorectal cancer. R_e and N_e denote the number of responders and the total number of patients in the experimental arm and R_c and N_c denote these numbers in the control arm.

2 | BETA DISTRIBUTION BASED PRIOR FOR THE CORRELATION

A beta distribution was used to construct prior distributions for the between-studies correlations (all models) and for the correlation between effects on treatment arms (models 2a–c). A random variable drawn from a beta distribution $r \sim Beta(1.5, 1.5)$ is limited to values between 0 and 1 with probability density zero on the edges and mean value of 0.5, as seen in Figure 1 (left). Transforming this variable, such as $\rho = 2r - 1$, gives a distribution bounded by -1 and 1 with mean at zero, as shown in Figure 1 (right). This can be used as a prior distribution for the between-studies correlation, as in Burke et al (2016). The resulting prior distribution for the correlation, such as $\frac{\rho+1}{2} \sim Beta(1.5, 1.5)$, allows for positive and negative values of the between-studies correlation and it is relatively flat across the range of values, with the exception that values at the extreme ends of the distribution are considered extremely unlikely.

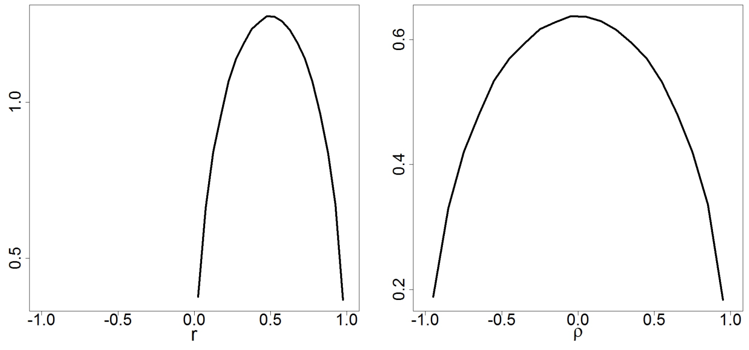

FIGURE 1 Beta distribution

3 | DETAILS OF MODEL 1B

We assume that the treatment effect differences Y_{jkli} between treatments k and l in study i for the two outcomes $j = 1, 2$ (the surrogate endpoints and the final clinical outcome) are correlated and normally distributed:

$$\begin{pmatrix} Y_{1kli} \\ Y_{2kli} \end{pmatrix} \sim \text{MVN}\left(\begin{pmatrix} \mu_{1kli} \\ \mu_{2kli} \end{pmatrix}, \mathbf{\Sigma_i}\right), \ \mathbf{\Sigma_i} = \begin{pmatrix} \sigma^2_{1kli} & \sigma_{1kli}\sigma_{2kli}\rho_{wkli} \\ \sigma_{1kli}\sigma_{2kli}\rho_{wkli} & \sigma^2_{2kli} \end{pmatrix} \tag{1}$$

To take into account the network structure of the data, we assume that the correlated true treatment effects μ_{1kli} and μ_{2kli} within each treatment contrast kl follow a common distribution:

$$\begin{pmatrix} \mu_{1kli} \\ \mu_{2kli} \end{pmatrix} \sim \text{MVN}\left(\begin{pmatrix} d_{1kl} \\ d_{2kl} \end{pmatrix}, \begin{pmatrix} \tau^2_{1kl} & \tau_{1kl}\tau_{2kl}\rho_{1kl,2kl} \\ \tau_{1kl}\tau_{2kl}\rho_{1kl,2kl} & \tau^2_{2kl} \end{pmatrix}\right) \tag{2}$$

where k and l denote baseline (control) and experimental treatment respectively in a study i, μ_{jkli} denotes the random true treatment effect (difference between the effects of treatments k and l) on outcome j in study i and, and the d_{jkl} are mean treatment effect differences between treatments k and l for each outcome j.

We use the first-order consistency assumptions, as described by Lu and Ades (2009), extended here to the bivariate case. For any three treatments (b, k, l), the treatment differences (μ_{jkli}) satisfy the following transitivity relations

$$\begin{pmatrix} \mu_{1kli} \\ \mu_{2kli} \end{pmatrix} = \begin{pmatrix} \mu_{1bli} - \mu_{1bki} \\ \mu_{2bli} - \mu_{2bki} \end{pmatrix}. \tag{3}$$

Taking the expectation of the transitivity equations gives the consistency equations for the first-order moments

$$\begin{pmatrix} d_{1kl} \\ d_{2kl} \end{pmatrix} = \begin{pmatrix} d_{1bl} - d_{1bk} \\ d_{2bl} - d_{2bk} \end{pmatrix} \tag{4}$$

which represent the relationships between the treatment contrasts in the population. When $b = 1$ is a common reference treatment in the network, the treatment effects of each treatment k in the network relative to this common reference treatment 1; the $d_{j,1k}$ are referred to as basic parameters for each outcome j, with $d_{j,11} = 0$ and the others are given prior distributions:

$$d_{j,1k} \sim N(0, 10^3). \tag{5}$$

To assume consistency of the second-order moments, we extend the approach proposed by Lu and Ades (2009) to the bivariate case by taking variance of the transitivity equation (3), which gives

$$\begin{aligned} &\begin{pmatrix} \tau^2_{1kl} & \tau_{1kl}\tau_{2kl}\rho_{1kl,2kl} \\ \tau_{1kl}\tau_{2kl}\rho_{1kl,2kl} & \tau^2_{2kl} \end{pmatrix} \\ &= \begin{pmatrix} var(\mu_{1bli} - \mu_{1bki}) & cov(\mu_{1bli} - \mu_{1bki}, \mu_{2bli} - \mu_{2bki}) \\ cov(\mu_{1bli} - \mu_{1bki}, \mu_{2bli} - \mu_{2bki}) & var(\mu_{2bli} - \mu_{2bki}) \end{pmatrix} \\ &= \begin{pmatrix} \tau^2_{1bk} + \tau^2_{1bl} - 2\tau_{1bk}\tau_{1bl}\rho_{1bk,1bl} & \begin{matrix} \tau_{1bl}\tau_{2bl}\rho_{1bl,2bl} + \tau_{1bk}\tau_{2bk}\rho_{1bk,2bk} \\ -\tau_{1bl}\tau_{2bk}\rho_{1bl,2bk} - \tau_{1bk}\tau_{2bl}\rho_{1bk,2bl} \end{matrix} \\ \begin{matrix} \tau_{1bl}\tau_{2bl}\rho_{1bl,2bl} + \tau_{1bk}\tau_{2bk}\rho_{1bk,2bk} \\ -\tau_{1bl}\tau_{2bk}\rho_{1bl,2bk} - \tau_{1bk}\tau_{2bl}\rho_{1bk,2bl} \end{matrix} & \tau^2_{2bk} + \tau^2_{2bl} - 2\tau_{2bk}\tau_{2bl}\rho_{2bk,2bl} \end{pmatrix} \end{aligned} \tag{6}$$

leading to the following relationship between the variances for any three treatments (b, k, l) and for both outcomes $j = 1, 2$;

$$\tau^2_{jkl} = \tau^2_{jbk} + \tau^2_{jbl} - 2\rho_{jbk,jbl}\tau_{jbk}\tau_{jbl} \leq (\tau_{jbk} + \tau_{jbl})^2, \tag{7}$$

which gives the second-order consistency conditions (triangle inequalities):

$$|\tau_{jbl} - \tau_{jbk}| \leq \tau_{jkl} \leq \tau_{jbl} + \tau_{jbk}. \tag{8}$$

In addition, the following condition applies to the covariances:

$$\begin{aligned} \tau_{1kl}\tau_{2kl}\rho_{1kl,2kl} = {} & \tau_{1bl}\tau_{2bl}\rho_{1bl,2bl} + \tau_{1bk}\tau_{2bk}\rho_{1bk,2bk} \\ & -\tau_{1bl}\tau_{2bk}\rho_{1bl,2bk} - \tau_{2bl}\tau_{1bk}\rho_{2bl,1bk}, \end{aligned} \tag{9}$$

which implies further constraints that are more complex than those in Eq. (8).

To ensure that prior distributions for heterogeneous variance-covariance matrices are appropriate, i.e. to maintain the second-order consistency condition for any three treatments in the network, ancillary parameters are used, allowing the between-studies variance-covariance matrices to be represented as

$$\begin{pmatrix} \tau_{1kl}^2 & \tau_{1kl}\tau_{2kl}\rho_{1kl,2kl} \\ \tau_{1kl}\tau_{2kl}\rho_{1kl,2kl} & \tau_{2kl}^2 \end{pmatrix} = \begin{pmatrix} \gamma_{1k}^2 + \gamma_{1l}^2 - 2\xi_{1k,1l}\gamma_{1k}\gamma_{1l} & \begin{matrix}\gamma_{1k}\gamma_{2k}\xi_{1k,2k} - \gamma_{1k}\gamma_{2l}\xi_{1k,2l} \\ -\gamma_{1l}\gamma_{2k}\xi_{1l,2k} + \gamma_{1l}\gamma_{2l}\xi_{1l,2l}\end{matrix} \\ \begin{matrix}\gamma_{1k}\gamma_{2k}\xi_{1k,2k} - \gamma_{1k}\gamma_{2l}\xi_{1k,2l} \\ -\gamma_{1l}\gamma_{2k}\xi_{1l,2k} + \gamma_{1l}\gamma_{2l}\xi_{1l,2l}\end{matrix} & \gamma_{2k}^2 + \gamma_{2l}^2 - 2\xi_{2k,2l}\gamma_{2k}\gamma_{2l} \end{pmatrix} \quad (10)$$

where γ_{jk}^2 and γ_{jl}^2 are the ancillary parameters: variances of two random effects ζ_{jki} and ζ_{jli} corresponding to treatment arms k and l (for each outcome $j = 1, 2$); and $\xi_{jk,j'l}$ is their correlation coefficient. Prior distributions for the set of between-studies standard deviations τ_{jkl} for each outcome j and each pair of treatments k and l can be given by constructing a prior distribution for a variance-covariance matrix Γ composed of the standard deviations γ_{jk} and correlations $\xi_{jk,j'l}$, for $j, j' = 1, 2$ and $k, l = 1, \ldots, n_t$, where n_t is the number of treatments in the network. For the set of values of the elements of matrix Γ, together with the relationship (10), to give a resulting set of standard deviations τ_{jkl} and correlations $\rho_{1kl,2kl}$ that satisfy the second-order consistency rules (8) and (9), the matrix Γ has to be positive semi-definite. This can be achieved in a number of ways, for example by spherical decomposition as proposed by Lu and Ades (2009). Such a spherical decomposition was later applied to construct a prior distribution for a variance-covariance matrix in multivariate meta-analysis by Wei and Higgins (2013), who also investigated use of the inverse Wishart prior distribution and a separation strategy with a Cholesky decomposition. Here we use the latter approach where $\Gamma = V^{1/2}RV^{1/2}$, where $V^{1/2}$ is a $2n_t \times 2n_t$ diagonal matrix of the standard deviations $\gamma_{11}, \gamma_{21}, \ldots, \gamma_{1n_t}, \gamma_{2n_t}$ and R is a positive semi-definite $2n_t \times 2n_t$ matrix of correlations $\xi_{jk,j'l}$ (block matrix consisting of $n_t \times n_t$ blocks that are of 2×2 dimension). Matrix R can be represented, using the Cholesky separation strategy, as $R = L^T L$ with L being a $2n_t \times 2n_t$ upper triangular matrix. To obtain the elements of the matrix L, we follow the method by Wei and Higgins (2013). The elements of the top row of the correlation matrix are

$$R_{1j} = L_{11}L_{1j},$$

the diagonal elements are

$$R_{jj} = \sum_{k=1}^{j} L_{kj}^2$$

and the remaining elements are

$$R_{ij} = \sum_{k=1}^{i} L_{ki}L_{kj}$$

where$j > i,\ i = 1, \ldots, 2n_t - 1,\ j = 1, \ldots, 2n_t$. Prior distributions are placed on the elements of matrix L in such a way to ensure the correlations are constrained to the range of values between -1 and 1. This is achieved, following Wei and Higgins (2013), by selecting plausible intervals for these elements. For the top row of matrix L we set uniform prior distributions on the following intervals:

$$L_{1j} \in [-1, 1]$$

and the intervals for the remaining off-diagonal elements are

$$L_{ij} \in \left[-\sqrt{1 - \sum_{k=1}^{i-1} L_{kl}^2}, \sqrt{1 - \sum_{k=1}^{i-1} L_{kl}^2} \right]$$

which gives implied prior distributions for the diagonal elements:

$$L_{jj} = \sqrt{1 - \sum_{k=1}^{j-1} L_{kj}^2}.$$

Prior distributions are placed on the standard deviations, which need to be restricted to positive values, for example $\gamma_{j,k} \sim unif(0, 2)$. The prior distributions placed on the ancillary variances and correlations give implied prior distributions on the between-studies correlations and standard deviations through the formulae (10).

3.1 | WinBUGS code for model 1b

```
model{
for(i in 1:ns) {
prec_w[i,1:2,1:2] <- inverse(Sigma[i,1:2,1:2])
#covariance matrix for the j-th study
Sigma[i,1,1]<-pow(se[i,1],2)
Sigma[i,2,2]<-pow(se[i,2],2)
Sigma[i,1,2]<-sqrt(Sigma[i,1,1])*sqrt(Sigma[i,2,2])*rho_w[i]
Sigma[i,2,1]<-sqrt(Sigma[i,1,1])*sqrt(Sigma[i,2,2])*rho_w[i]
y[i,1:2] ~ dmnorm(delta[i,1:2],prec_w[i,1:2,1:2])
delta[i,1:2] ~ dmnorm(md[i,1:2],prec_b[i,1:2,1:2])  # trial-specific treat effects distributions
for(j in 1:2) {
md[i,j] <- d[t[i,2],j] - d[t[i,1],j]   # mean of treat effects distributions
}
prec_b[i,1:2,1:2]<-inverse(Cov_b[tc[i],,])
}

for (ic in 1:nc){
for (j in 1:2) {
Cov_b[ic,j,j]<-tau.sq[ic,j]
tau.sq[ic,j]<-psi.sq[(td[ic,1]-1)*2+j]+psi.sq[(td[ic,2]-1)*2+j]
-2*rho_psi[(td[ic,1]-1)*2+j,(td[ic,2]-1)*2+j]*psi[(td[ic,1]-1)*2+j]*psi[(td[ic,2]-1)*2+j]
}
Cov_b[ic,1,2]<- psi[(td[ic,1]-1)*2+1]*psi[(td[ic,1]-1)*2+2]*rho_psi[(td[ic,1]-1)*2+1,(td[ic,1]-1)*2+2]
   -psi[(td[ic,1]-1)*2+1]*psi[(td[ic,2]-1)*2+2]*rho_psi[(td[ic,1]-1)*2+1,(td[ic,2]-1)*2+2]
   -psi[(td[ic,2]-1)*2+1]*psi[(td[ic,1]-1)*2+2]*rho_psi[(td[ic,2]-1)*2+1,(td[ic,1]-1)*2+2]
   +psi[(td[ic,2]-1)*2+1]*psi[(td[ic,2]-1)*2+2]*rho_psi[(td[ic,2]-1)*2+1,(td[ic,2]-1)*2+2]
Cov_b[ic,2,1]<-Cov_b[ic,1,2]
rho_b[ic]<-Cov_b[ic,1,2]/sd[ic,1]/sd[ic,2]
sd[ic,1]<-sqrt(tau.sq[ic,1])
sd[ic,2]<-sqrt(tau.sq[ic,2])
lambda0[ic]<- (d[td[ic,2],2]-d[td[ic,1],2])
- (d[td[ic,2],1]-d[td[ic,1],1]) *rho_b[ic]* sd[ic,2]/sd[ic,1]
}

for(j in 1:2) {d[1,j] <- 0}

for (k in 1:nt2){
psi[k]~dunif(0,2)
psi.sq[k]<-pow(psi[k],2)
rho_psi[k,k]<-1
}
for (k in 2:nt){
d[k,1] ~ dnorm(0,0.001)
d[k,2] ~ dnorm(0,0.001)
}

# assigning priors to the upper triangular matrix in Cholesky decomposition
L[1,1]<-1.0
for (k in 2:nt2){
L.u[1,k]~dunif(-0.999,0.999)
```

```
L[1,k] <- L.u[1,k]
}
for (x in 1:nt2-1){
for (k in x+1:nt2){
  p[x,k]<-pow(L[x,k],2)
}
}
for (x in 3:nt2){
 for (k in x:nt2){
s[x-1,k]<-sum(p[1:x-2,k])
lim[x-1,k]<- sqrt(1-s[x-1,k])
L.u[x-1,k]~dunif(-0.999,0.999)
L[x-1,k]<- lim[x-1,k] * L.u[x-1,k]
 }
}
L.u[2,2]<-sqrt(1-pow(L[1,2],2))
L[2,2]<-L.u[2,2]
for (k in 3:nt2){
s2[k]<-sum(p[1:k-1,k])
 L.u[k,k]<-sqrt(1-s2[k])
 L[k,k]<-L.u[k,k]
}

#assigning values for the correlations:
for (k in 2:nt2){
rho_psi[1,k]<-L[1,k]
rho_psi[k,1]<-L[1,k]
}
for (x in 2:nt2-1){
  for (k in x+1:nt2){
    for (j in 1:x){
LL[j,x,k]<-L[j,x]*L[j,k]
}
}
}
for (x in 2:nt2-2){
  for (k in x+1:nt2){
rho_psi[x,k]<-sum(LL[1:x,x,k])
rho_psi[k,x]<-rho_psi[x,k]
}
}
rho_psi[nt2-1,nt2]<- sum(LL[1:nt2-1,nt2-1,nt2])
rho_psi[nt2,nt2-1]<- rho_psi[nt2-1,nt2]
}
```

```
##### Example of data structure #######

list(ns=30, nt=3, nt2=6, nc=3, td= structure(.Data= c(1, 2, 2, 3, 1, 3), .Dim=c(3, 2)))
# ns - number of studies,  nt - number of treatments, nt2=nt*2 (size of the Gamma matrix)
# nc - number of contrasts,  td - list of contrasts (designs)

t[,1] t[,2] tc[] y[,1] y[,2] se[,1] se[,2] rho_w[]
1 2 1 1.46 2.17 0.24 0.24 0.6
1 2 1 1.10 2.22 0.21 0.21 0.6
1 2 1 0.58 1.29 0.23 0.23 0.6
1 2 1 1.41 2.63 0.20 0.20 0.6
1 2 1 1.36 3.00 0.23 0.23 0.6
1 2 1 0.86 1.68 0.22 0.22 0.6
1 2 1 1.24 2.25 0.22 0.22 0.6
1 2 1 0.60 1.13 0.25 0.25 0.6
1 2 1 0.91 1.70 0.19 0.19 0.6
1 2 1 1.59 2.67 0.20 0.20 0.6
2 3 2 2.12 1.02 0.22 0.22 0.6
2 3 2 1.58 0.84 0.20 0.20 0.6
2 3 2 1.82 0.99 0.19 0.19 0.6
2 3 2 2.35 1.34 0.22 0.22 0.6
2 3 2 1.76 0.59 0.23 0.23 0.6
2 3 2 2.57 1.41 0.18 0.18 0.6
2 3 2 2.28 1.24 0.23 0.23 0.6
2 3 2 2.66 1.44 0.20 0.20 0.6
2 3 2 1.02 0.63 0.25 0.25 0.6
2 3 2 2.19 1.34 0.20 0.20 0.6
1 3 3 2.20 2.25 0.21 0.21 0.6
1 3 3 3.47 3.17 0.21 0.21 0.6
1 3 3 2.86 2.49 0.23 0.23 0.6
1 3 3 2.84 3.15 0.19 0.19 0.6
1 3 3 3.97 3.63 0.20 0.20 0.6
1 3 3 3.39 3.45 0.16 0.16 0.6
1 3 3 3.67 3.84 0.19 0.19 0.6
1 3 3 1.99 2.30 0.19 0.19 0.6
1 3 3 3.13 3.24 0.22 0.22 0.6
1 3 3 2.32 2.19 0.21 0.21 0.6
END

### initial values
list(d= structure(.Data= c(NA,NA, 0.5, 0.5, 0.5, 0.5), .Dim=c(3, 2)),
delta= structure(.Data= c(0.5, 0.5, 0.5, 0.5, 0.5, 0.5, 0.5, 0.5, 0.5,0.5,
                          0.5, 0.5, 0.5, 0.5, 0.5, 0.5, 0.5, 0.5, 0.5, 0.5,
                          0.5, 0.5, 0.5, 0.5, 0.5, 0.5, 0.5, 0.5, 0.5, 0.5,
                          0.5, 0.5, 0.5, 0.5, 0.5, 0.5, 0.5, 0.5, 0.5, 0.5,
                          0.5, 0.5, 0.5, 0.5, 0.5, 0.5, 0.5, 0.5, 0.5, 0.5,
                          0.5, 0.5, 0.5, 0.5, 0.5, 0.5, 0.5, 0.5, 0.5, 0.5), .Dim=c(30, 2)),
psi=c(0.5, 0.5, 0.5, 0.5, 0.5, 0.5),
L.u= structure(.Data= c(NA, 0.2, 0.2, 0.2, 0.2, 0.2, NA, NA, 0.2, 0.2, 0.2, 0.2,
                        NA, NA, NA, 0.2, 0.2, 0.2, NA, NA, NA, NA, 0.2, 0.2,
                        NA, NA, NA, NA, NA, 0.2, NA, NA, NA, NA, NA, NA), .Dim=c(6, 6)))
```

4 | RELATIONSHIPS BETWEEN THE MODELS

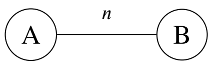

FIGURE 2 Example network diagram: all n studies include the same treatment contrast (only two treatments) (left).

The models reduce to the standard meta-analysis model for surrogate endpoints, such as the BRMA model, in a special case of data structure. When there are only two treatments in the network, as depicted in Figure 2, it can be shown that model 1a reduces to BRMA. Equations (1)–(2) become

$$\begin{pmatrix} Y_{1(12)i} \\ Y_{2(12)i} \end{pmatrix} \sim \mathrm{N}\left(\begin{pmatrix} \mu_{1(12)i} \\ \mu_{2(12)i} \end{pmatrix}, \begin{pmatrix} \sigma^2_{1(12)i} & \sigma_{1(12)i}\sigma_{2(12)i}\rho_{wi(12)} \\ \sigma_{1(12)i}\sigma_{2(12)i}\rho_{wi(12)} & \sigma^2_{2(12)i} \end{pmatrix} \right) \tag{11}$$

$$\begin{pmatrix} \mu_{1(12)i} \\ \mu_{2(12)i} \end{pmatrix} \sim \mathrm{MVN}\left(\begin{pmatrix} d_{1(12)} \\ d_{2(12)} \end{pmatrix}, \begin{pmatrix} \tau^2_{1(12)} & \tau_{1(12)}\tau_{2(12)}\rho_{(12)} \\ \tau_{1(12)}\tau_{2(12)}\rho_{(12)} & \tau^2_{2(12)} \end{pmatrix} \right) \tag{12}$$

The index (12) denoting the two treatments does not vary across studies or contrasts and hence can be dropped, resulting in equations for BRMA – equations (3.1)–(3.2) in the main manuscript, with $d_j = \beta_j$ and $j = 1, 2$.

5 | SURROGACY CRITERIA

Daniels and Hughes defined the surrogacy criteria for a Bayesian meta-analytic model where the relationship between the true treatment effects on final clinical outcome μ_{2i} and the effect on the surrogate endpoint μ_{1i} was written in the form of a linear regression:

$$\mu_{2i}|\mu_{1i} \sim N(\lambda_0 + \lambda_1\mu_{1i}, \psi^2). \tag{13}$$

The surrogate relationship between the two treatment effects, μ_{2i} and μ_{1i}, was perfect if the intercept λ_0 was zero, as then a zero effect on a surrogate would imply a zero effect on the final outcome, the slope λ_1 should not be zero for the association to be strong, with the conditional variance ψ^2 being zero. For the complete model see Daniels and Hughes (1997). A similar relationship and surrogacy criteria were described by Bujkiewicz et al (2015) in the framework of bivariate meta-analysis and extended by Bujkiewicz et al (2016) to multivariate meta-analysis. In the two papers the relationship between the regression parameters and the elements of the between-studies variance-covariance matrix was defined, similarly as in Bujkiewicz et al (2013). The derived relationships in the bivariate case are

$$\lambda_1 = \rho\frac{\tau_2}{\tau_1} \tag{14}$$

and

$$\psi^2 = \tau_2^2 - \lambda_1^2\tau_1^2. \tag{15}$$

If the surrogacy relationship is perfect, the conditional variance is zero: $\psi^2 = 0$ (Daniels and Hughes (1997), Bujkiewicz et al (2015)). Hence, from (15), $\tau_2^2 = \lambda_1^2\tau_1^2$ which gives $\lambda_1 = \pm\frac{\tau_2}{\tau_1}$, and from (14) it implies that the correlation $\rho = \pm 1$. Also $\rho^2 = 1$, which some authors refer to as the study level adjusted R-squared (Burzykowski et al (2001), Renfro et al (2012)).

If the surrogacy relationship is perfect, the intercept is also zero: $\lambda_0 = 0$. The intercept can be expressed in terms of the parameters of bvMA as follows: the slope is defined by (14) and also $\lambda_1 = (d_2 - \lambda_0)/d_1$, which leads to

$$\lambda_0 = d_2 - d_1\rho\tau_2/\tau_1. \tag{16}$$

6 | ADDITIONAL RESULTS: ACRC EXAMPLE

model	AB	AC	BC	BD
log OR (TR)				
BRMA	0.53 (0.36, 0.71)			
bvNMA 1a	0.48 (0.25, 0.71)	0.79 (0.55, 1.07)	0.32 (0.09, 0.57)	0.17 (-0.66, 0.93)
bvNMA 1b	0.47 (0.22, 0.73)	0.77 (0.51, 1.05)	0.3 (0.02, 0.6)	0.16 (-0.69, 0.93)
bvNMA 1c	0.47 (0.23, 0.7)	0.76 (0.53, 1)	0.3 (0.05, 0.55)	0.21 (-0.23, 0.62)
bvNMA 1d	0.49 (0.21, 0.77)	0.74 (0.49, 0.99)	0.25 (-0.08, 0.59)	0.2 (-0.38, 0.77)
bvNMA 2a	0.45 (0.22, 0.68)	0.73 (0.48, 0.99)	0.28 (0.05, 0.52)	0.12 (-0.45, 0.65)
bvNMA 2b	0.44 (0.18, 0.69)	0.71 (0.45, 0.98)	0.27 (0, 0.54)	0.11 (-0.44, 0.62)
bvNMA 2c	0.43 (0.21, 0.66)	0.7 (0.48, 0.94)	0.27 (0.05, 0.5)	0.17 (-0.22, 0.54)
bvNMA 2d	0.44 (0.18, 0.71)	0.68 (0.44, 0.93)	0.24 (-0.07, 0.55)	0.13 (-0.34, 0.61)
log HR (PFS)				
BRMA	-0.24 (-0.32, -0.15)			
bvNMA 1a	-0.36 (-0.46, -0.26)	-0.3 (-0.41, -0.19)	0.06 (-0.04, 0.19)	0.08 (-0.33, 0.47)
bvNMA 1b	-0.36 (-0.47, -0.26)	-0.29 (-0.4, -0.17)	0.08 (-0.05, 0.21)	0.08 (-0.27, 0.44)
bvNMA 1c	-0.36 (-0.46, -0.26)	-0.29 (-0.39, -0.19)	0.07 (-0.04, 0.19)	0.08 (-0.12, 0.28)
bvNMA 1d	-0.37 (-0.49, -0.25)	-0.27 (-0.38, -0.17)	0.1 (-0.05, 0.25)	0.1 (-0.15, 0.35)
bvNMA 2a	-0.34 (-0.44, -0.24)	-0.28 (-0.38, -0.17)	0.06 (-0.04, 0.18)	0.1 (-0.15, 0.36)
bvNMA 2b	-0.34 (-0.45, -0.23)	-0.27 (-0.38, -0.16)	0.07 (-0.05, 0.2)	0.1 (-0.14, 0.34)
bvNMA 2c	-0.34 (-0.43, -0.24)	-0.27 (-0.36, -0.17)	0.07 (-0.04, 0.18)	0.09 (-0.08, 0.26)
bvNMA 2d	-0.34 (-0.46, -0.23)	-0.26 (-0.36, -0.16)	0.09 (-0.05, 0.22)	0.12 (-0.09, 0.33)
Var(log OR) (TR)				
BRMA	0.29 (0.15, 0.49)			
bvNMA 1a	0.22 (0.07, 0.56)	0.57 (0.23, 1.22)	0.1 (0, 0.68)	0.76 (0.03, 3.18)
bvNMA 1b	0.3 (0.1, 0.66)	0.44 (0.2, 0.86)	0.15 (0, 0.67)	0.63 (0.03, 2.85)
bvNMA 1c	0.23 (0.09, 0.47)	0.33 (0.16, 0.6)	0.08 (0, 0.34)	0.12 (0, 0.49)
bvNMA 1d	0.3 (0.15, 0.52)			
bvNMA 2a	0.22 (0.07, 0.54)	0.55 (0.22, 1.2)	0.11 (0, 0.75)	0.7 (0.02, 3.11)
bvNMA 2b	0.3 (0.1, 0.65)	0.43 (0.2, 0.83)	0.14 (0, 0.6)	0.53 (0.03, 2.47)
bvNMA 2c	0.23 (0.09, 0.48)	0.3 (0.14, 0.56)	0.07 (0, 0.26)	0.12 (0, 0.45)
bvNMA 2d	0.29 (0.15, 0.5)			
Var(log HR) (PFS)				
BRMA	0.08 (0.04, 0.13)			
bvNMA 1a	0.03 (0.01, 0.09)	0.1 (0.04, 0.2)	0.03 (0, 0.23)	0.21 (0.01, 1.36)
bvNMA 1a	0.04 (0.01, 0.11)	0.08 (0.04, 0.15)	0.04 (0, 0.13)	0.14 (0.01, 0.8)
	0.03 (0.01, 0.08)	0.06 (0.03, 0.11)	0.02 (0, 0.07)	0.02 (0, 0.08)
	0.05 (0.03, 0.09)			
bvNMA 2a	0.03 (0.01, 0.09)	0.1 (0.04, 0.19)	0.02 (0, 0.17)	0.16 (0, 1)
bvNMA 2b	0.04 (0.01, 0.11)	0.08 (0.04, 0.14)	0.03 (0, 0.12)	0.11 (0.01, 0.52)
bvNMA 2c	0.03 (0.01, 0.08)	0.05 (0.02, 0.1)	0.02 (0, 0.07)	0.02 (0, 0.08)
bvNMA 2d	0.05 (0.03, 0.09)			

TABLE 3 Mean effects and the between-studies variances for each model in the aCRC example. A – chemotherapy alone, B – anti-VEGF therapies + chemotherapy, C – EGFRi + chemotherapy, D – EGFRi + anti-VEGF therapies + chemotherapy.

model	AE	AD	CF
log OR (TR)			
bvNMA 1a	1.4 (-1.8, 4.66)	0.64 (-0.19, 1.42)	-0.01 (-1.51, 1.46)
bvNMA 1b	1.42 (-2.05, 4.82)	0.64 (-0.23, 1.42)	-0.03 (-1.6, 1.53)
bvNMA 1c	1.4 (-1.04, 3.85)	0.67 (0.18, 1.14)	-0.02 (-0.72, 0.67)
bvNMA 1d	1.38 (-1.06, 3.86)	0.68 (0.07, 1.3)	-0.02 (-0.93, 0.88)
bvNMA 2a	0.58 (-0.41, 1.82)	0.57 (-0.01, 1.14)	-0.14 (-0.83, 0.51)
bvNMA 2b	0.54 (-0.47, 1.78)	0.55 (-0.02, 1.1)	-0.16 (-0.89, 0.56)
bvNMA 2c	0.59 (-0.3, 1.8)	0.6 (0.17, 1.01)	-0.11 (-0.62, 0.42)
bvNMA 2d	0.57 (-0.32, 1.79)	0.57 (0.08, 1.09)	-0.12 (-0.73, 0.54)
log HR (PFS)			
bvNMA 1a	0 (-2.55, 2.55)	-0.28 (-0.7, 0.12)	0.11 (-1.24, 1.44)
bvNMA 1b	0 (-2.59, 2.57)	-0.28 (-0.64, 0.09)	0.12 (-1.24, 1.48)
bvNMA 1c	0 (-0.68, 0.68)	-0.28 (-0.49, -0.06)	0.11 (-0.25, 0.48)
bvNMA 1d	0 (-0.67, 0.68)	-0.27 (-0.54, -0.01)	0.11 (-0.3, 0.53)
bvNMA 2a	-0.17 (-0.68, 0.38)	-0.23 (-0.5, 0.03)	0.08 (-0.27, 0.46)
bvNMA 2b	-0.18 (-0.71, 0.36)	-0.24 (-0.49, 0)	0.07 (-0.31, 0.45)
bvNMA 2c	-0.11 (-0.47, 0.3)	-0.24 (-0.43, -0.06)	0.09 (-0.16, 0.35)
bvNMA 2d	-0.1 (-0.46, 0.32)	-0.22 (-0.45, -0.01)	0.1 (-0.18, 0.4)
correlations			
bvNMA 1a	0 (-0.88, 0.88)	-0.02 (-0.89, 0.88)	-0.03 (-0.9, 0.9)
bvNMA 1b	-0.07 (-0.88, 0.83)	-0.31 (-0.94, 0.73)	-0.04 (-0.85, 0.81)
bvNMA 1c	-0.42 (-0.95, 0.65)	-0.44 (-0.95, 0.6)	-0.16 (-0.87, 0.74)
bvNMA 2a	0.01 (-0.88, 0.89)	-0.02 (-0.9, 0.88)	-0.04 (-0.91, 0.88)
bvNMA 2b	-0.07 (-0.89, 0.84)	-0.33 (-0.95, 0.71)	-0.06 (-0.85, 0.8)
bvNMA 2c	-0.43 (-0.95, 0.64)	-0.47 (-0.95, 0.57)	-0.13 (-0.86, 0.75)
intercepts			
bvNMA 1a	-0.41 (-6.44, 6.4)	0.17 (-3.31, 2.82)	1.67 (-1.56, 25.47)
bvNMA 1b	0.11 (-3.61, 3.85)	-0.18 (-0.78, 0.43)	0.12 (-1.2, 1.46)
bvNMA 1c	0.29 (-0.75, 1.68)	-0.14 (-0.54, 0.31)	0.11 (-0.29, 0.51)
bvNMA 1d	0.45 (-0.28, 1.24)	-0.05 (-0.28, 0.17)	0.11 (-0.21, 0.43)
bvNMA 2a	-0.17 (-2.82, 2.27)	-1.47 (-3.02, 2.38)	0.19 (-0.73, 1)
bvNMA 2b	-0.15 (-1.35, 1.03)	-0.15 (-0.62, 0.32)	0.07 (-0.5, 0.63)
bvNMA 2c	0.01 (-0.48, 0.65)	-0.12 (-0.46, 0.26)	0.09 (-0.22, 0.41)
bvNMA 2d	0.08 (-0.28, 0.58)	-0.04 (-0.24, 0.15)	0.06 (-0.18, 0.32)
Var(log OR) (TR)			
bvNMA 1a	1.34 (0, 3.81)	0.98 (0, 3.65)	0.79 (0, 3.56)
bvNMA 1b	1.67 (0.08, 5.17)	0.8 (0.03, 3.25)	0.95 (0.01, 3.79)
bvNMA 1c	0.3 (0.03, 1.02)	0.27 (0.02, 0.83)	0.13 (0, 0.56)
bvNMA 2a	1.21 (0, 3.76)	0.95 (0, 3.63)	0.56 (0, 3.13)
bvNMA 2b	1.5 (0.07, 4.92)	0.7 (0.03, 2.86)	0.7 (0.01, 3.39)
bvNMA 2c	0.29 (0.03, 0.97)	0.27 (0.03, 0.83)	0.11 (0, 0.49)
Var(log HR) (PFS)			
bvNMA 1a	1.33 (0, 3.81)	0.8 (0, 3.56)	0.66 (0, 3.45)
bvNMA 1b	1.37 (0.02, 4)	0.16 (0.01, 0.84)	0.71 (0, 3.5)
bvNMA 1c	0.05 (0, 0.2)	0.05 (0, 0.14)	0.03 (0, 0.13)
bvNMA 2a	0.86 (0, 3.6)	0.79 (0, 3.54)	0.33 (0, 2.69)
bvNMA 2b	0.91 (0.01, 3.67)	0.13 (0.01, 0.58)	0.39 (0, 2.74)
bvNMA 2c	0.05 (0, 0.17)	0.04 (0, 0.14)	0.03 (0, 0.11)

TABLE 4 Mean effects and the between-studies correlations and variances for each model in the aCRC example (contrasts AE, AD and CF). A – chemotherapy alone, C – EGFRi + chemotherapy, D – EGFRi + anti-VEGF therapies + chemotherapy, E – anti-IGF1R , F – anti-IgG2 + chemotherapy

Figure 3 shows the predicted effects obtained from BRMA and model 2d along with the observed estimates of the effects on PFS. The improvement in predictions was not substantial due to the weak association patterns between the treatment effects on the two outcomes.

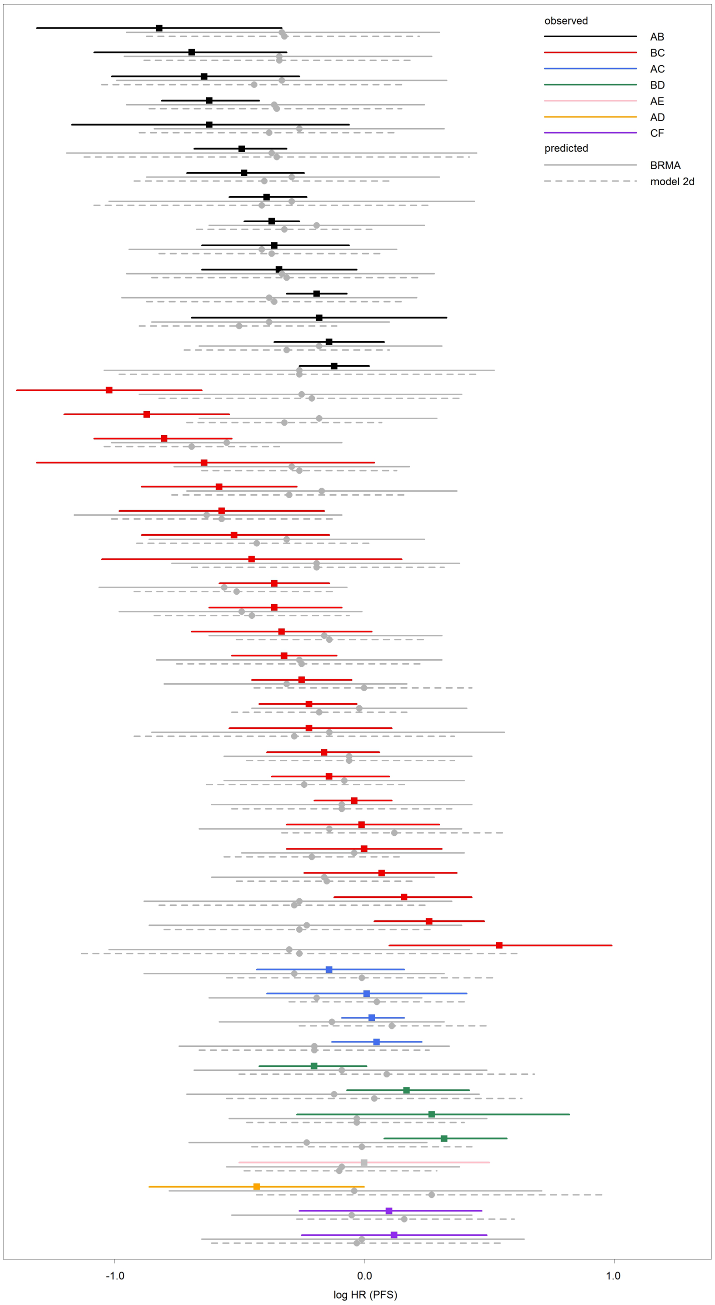

FIGURE 3 Predicted effects obtained from BRMA and model 2d along with the observed estimates of the effects on PFS for aCRC data

7 | SENSITIVITY ANALYSIS (ACRC EXAMPLE)

Sensitivity analysis was carried out investigating the effect of potentially influential observations (three studies with largest treatment effect on TR, due to no events in the control arm, were removed). Figure 4 shows the scatter plot. Tables 6 and 7 show the between studies correlations of the heterogeneity parameters.

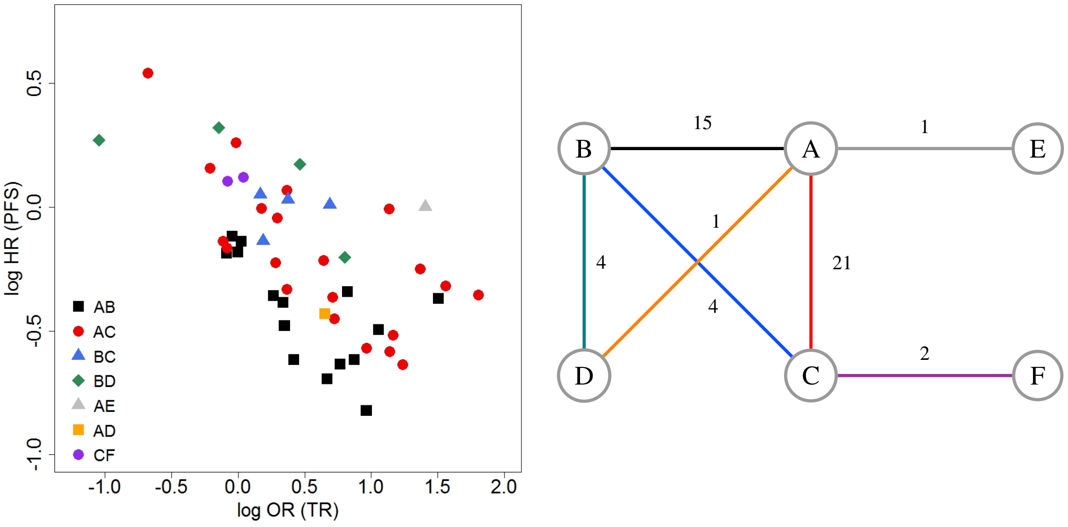

FIGURE 4 Scatter plot and network diagram for the advanced colorectal cancer example, A – chemotherapy alone, B – anti-VEGF therapies + chemotherapy, C – EGFRi + chemotherapy, D – EGFRi + anti-VEGF therapies + chemotherapy, E – anti-IGF1R , F – anti-IgG2 + chemotherapy

model	within-treatment surrogate relationship					
	AB	AC	BC	BD	ρ_t	DIC
between-studies correlations						
BRMA		-0.59 (-0.8, -0.3)			NA	28.2
bvNMA 1a	-0.44 (-0.84, 0.16)	-0.69 (-0.93, -0.27)	-0.05 (-0.9, 0.88)	-0.28 (-0.93, 0.68)	NA	29.9
bvNMA 1b	-0.57 (-0.89, -0.01)	-0.71 (-0.93, -0.33)	-0.22 (-0.89, 0.7)	-0.26 (-0.89, 0.6)	NA	29.2
bvNMA 1c	-0.56 (-0.87, -0.04)	-0.69 (-0.93, -0.3)	-0.18 (-0.86, 0.71)	-0.26 (-0.87, 0.58)	NA	45.6
bvNMA 1d		-0.68 (-0.87, -0.39)			NA	24.9
bvNMA 2a	-0.46 (-0.86, 0.13)	-0.68 (-0.93, -0.25)	-0.06 (-0.91, 0.86)	-0.29 (-0.94, 0.66)	-0.3 ((-0.9, 0.59)	28.0
bvNMA 2b	-0.58 (-0.89, -0.04)	-0.71 (-0.93, -0.33)	-0.22 (-0.89, 0.7)	-0.27 (-0.89, 0.59)	-0.29 (-0.91, 0.59	27.2
bvNMA 2c	-0.56 (-0.88, -0.06)	-0.68 (-0.93, -0.3)	-0.19 (-0.87, 0.7)	-0.26 (-0.87, 0.59)	-0.28 (-0.89, 0.6)	43.4
bvNMA 2d		-0.68 (-0.88, -0.39)			-0.31 (-0.91, 0.58)	23.1
intercepts						
BRMA	-0.06 (-0.16, 0.04)					
bvNMA 1a	-0.27 (-0.41, -0.12)	-0.05 (-0.19, 0.09)	0.44 (-0.79, 1.07)	0.12 (-0.31, 0.55)		
bvNMA 1b	-0.25 (-0.39, -0.12)	-0.04 (-0.17, 0.09)	0.14 (-0.12, 0.41)	0.11 (-0.24, 0.47)		
bvNMA 1c	-0.25 (-0.37, -0.13)	-0.06 (-0.18, 0.07)	0.14 (-0.11, 0.38)	0.11 (-0.12, 0.37)		
bvNMA 1d	-0.23 (-0.33, -0.14)	-0.05 (-0.15, 0.06)	0.19 (0.09, 0.29)	0.14 (-0.03, 0.32)		
bvNMA 2a	-0.25 (-0.38, -0.1)	-0.05 (-0.19, 0.08)	-0.47 (-2.54, 0.89)	0.19 (-0.16, 0.45)		
bvNMA 2b	-0.23 (-0.36, -0.1)	-0.05 (-0.17, 0.08)	0.13 (-0.11, 0.38)	0.12 (-0.13, 0.38)		
bvNMA 2c	-0.23 (-0.35, -0.11)	-0.06 (-0.18, 0.06)	0.13 (-0.1, 0.37)	0.12 (-0.09, 0.33)		
bvNMA 2d	-0.22 (-0.32, -0.12)	-0.05 (-0.15, 0.06)	0.17 (0.08, 0.27)	0.15 (-0.01, 0.30)		

TABLE 5 Between-studies correlations corresponding to the within-treatment surrogate relationship for each model, ρ_t – across-treatment correlations obtained from the models allowing for exchangeability, and DIC values corresponding to each model fitted to aCRC data. Where only one value is given for the between-studies correlation within a treatment contrast (models BRMA, 1d and 2d), the parameters are common across the treatment contrasts.
A – chemotherapy alone, B – anti-VEGF therapies + chemotherapy, C – EGFRi + chemotherapy, D – EGFRi + anti-VEGF therapies + chemotherapy

model	AB	BC	AC	BD
log OR (TR)				
BRMA	0.48 (0.31, 0.65)			
bvNMA 1a	0.42 (0.19, 0.65)	0.68 (0.44, 0.92)	0.26 (0, 0.5)	0.18 (-0.67, 0.94)
bvNMA 1b	0.42 (0.17, 0.67)	0.66 (0.41, 0.9)	0.24 (-0.06, 0.5)	0.17 (-0.66, 0.94)
bvNMA 1c	0.41 (0.19, 0.63)	0.66 (0.44, 0.88)	0.24 (0, 0.48)	0.22 (-0.2, 0.62)
bvNMA 1d	0.45 (0.2, 0.71)	0.62 (0.38, 0.85)	0.16 (-0.14, 0.47)	0.21 (-0.32, 0.73)
bvNMA 2a	0.39 (0.15, 0.62)	0.63 (0.37, 0.87)	0.24 (-0.02, 0.47)	0.12 (-0.43, 0.64)
bvNMA 2b	0.38 (0.13, 0.63)	0.6 (0.35, 0.85)	0.22 (-0.05, 0.47)	0.11 (-0.41, 0.61)
bvNMA 2c	0.38 (0.15, 0.6)	0.6 (0.37, 0.82)	0.23 (-0.01, 0.45)	0.17 (-0.19, 0.52)
bvNMA 2d	0.41 (0.16, 0.66)	0.57 (0.33, 0.8)	0.16 (-0.13, 0.45)	0.12 (-0.31, 0.57)
log HR (PFS)				
BRMA	-0.2 (-0.28, -0.12)			
bvNMA 1a	-0.34 (-0.44, -0.24)	-0.23 (-0.34, -0.12)	0.11 (0, 0.25)	0.08 (-0.34, 0.48)
bvNMA 1b	-0.34 (-0.45, -0.24)	-0.22 (-0.32, -0.12)	0.12 (0, 0.26)	0.08 (-0.28, 0.43)
bvNMA 1c	-0.34 (-0.43, -0.24)	-0.22 (-0.31, -0.13)	0.12 (0.01, 0.23)	0.07 (-0.12, 0.27)
bvNMA 1d	-0.35 (-0.46, -0.25)	-0.2 (-0.3, -0.11)	0.15 (0.02, 0.27)	0.09 (-0.12, 0.3)
bvNMA 2a	-0.32 (-0.42, -0.2)	-0.22 (-0.33, -0.12)	0.1 (-0.01, 0.23)	0.1 (-0.15, 0.36)
bvNMA 2b	-0.32 (-0.42, -0.2)	-0.21 (-0.31, -0.11)	0.11 (-0.01, 0.23)	0.1 (-0.13, 0.34)
bvNMA 2c	-0.31 (-0.41, -0.21)	-0.2 (-0.29, -0.12)	0.11 (0, 0.22)	0.09 (-0.07, 0.26)
bvNMA 2d	-0.32 (-0.43, -0.22)	-0.19 (-0.29, -0.1)	0.13 (0.02, 0.25)	0.12 (-0.06, 0.3)
Var(log OR) (TR)				
BRMA	0.24 (0.13, 0.41)			
bvNMA 1a	0.22 (0.07, 0.53)	0.38 (0.16, 0.81)	0.11 (0, 0.77)	0.79 (0.03, 3.25)
bvNMA 1b	0.26 (0.09, 0.56)	0.33 (0.15, 0.64)	0.13 (0, 0.57)	0.61 (0.03, 2.8)
bvNMA 1c	0.2 (0.08, 0.41)	0.25 (0.12, 0.46)	0.07 (0, 0.26)	0.11 (0, 0.42)
bvNMA 1d	0.23 (0.12, 0.41)			
bvNMA 2a	0.23 (0.07, 0.55)	0.38 (0.16, 0.8)	0.12 (0, 0.84)	0.69 (0.02, 3.06)
bvNMA 2b	0.26 (0.09, 0.56)	0.33 (0.15, 0.63)	0.12 (0, 0.54)	0.52 (0.03, 2.39)
bvNMA 2c	0.2 (0.08, 0.4)	0.25 (0.12, 0.45)	0.06 (0, 0.26)	0.1 (0.01, 0.37)
bvNMA 2d	0.23 (0.12, 0.4)			
Var(log HR) (PFS)				
BRMA	0.06 (0.03, 0.1)			
bvNMA 1a	0.03 (0.01, 0.09)	0.06 (0.02, 0.13)	0.06 (0, 0.41)	0.22 (0, 1.5)
bvNMA 1b	0.04 (0.01, 0.09)	0.05 (0.02, 0.1)	0.03 (0, 0.1)	0.14 (0.01, 0.77)
bvNMA 1c	0.03 (0.01, 0.06)	0.03 (0.01, 0.07)	0.02 (0, 0.06)	0.02 (0, 0.08)
bvNMA 1d	0.03 (0.01, 0.06)			
bvNMA 2a	0.03 (0.01, 0.1)	0.05 (0.02, 0.13)	0.05 (0, 0.3)	0.16 (0, 0.87)
bvNMA 2b	0.04 (0.01, 0.09)	0.05 (0.02, 0.1)	0.02 (0, 0.09)	0.1 (0.01, 0.53)
bvNMA 2c	0.03 (0.01, 0.07)	0.03 (0.01, 0.07)	0.02 (0, 0.06)	0.02 (0, 0.09)
bvNMA 2d	0.03 (0.01, 0.06)			

TABLE 6 Between-studies correlations for each model in the aCRC example. Where only one value is given (models BRMA, 1d and 2d), the parameters are common across the treatment contrasts.

model	AE	AD	CF
log OR (TR)			
bvNMA 1a	1.41 (-1.82, 4.68)	0.6 (-0.25, 1.38)	-0.02 (-1.44, 1.39)
bvNMA 1b	1.39 (-1.98, 4.76)	0.59 (-0.26, 1.38)	-0.02 (-1.57, 1.56)
bvNMA 1c	1.4 (-1.02, 3.86)	0.63 (0.17, 1.08)	-0.02 (-0.67, 0.65)
bvNMA 1d	1.42 (-0.95, 3.84)	0.66 (0.09, 1.23)	-0.02 (-0.84, 0.8)
bvNMA 2a	0.5 (-0.42, 1.69)	0.5 (-0.04, 1.06)	-0.12 (-0.78, 0.53)
bvNMA 2b	0.49 (-0.43, 1.7)	0.49 (-0.05, 1.04)	-0.13 (-0.8, 0.55)
bvNMA 2c	0.53 (-0.29, 1.67)	0.55 (0.14, 0.95)	-0.09 (-0.58, 0.4)
bvNMA 2d	0.51 (-0.32, 1.65)	0.53 (0.08, 1.01)	-0.09 (-0.65, 0.51)
log HR (PFS)			
bvNMA 1a	0 (-2.55, 2.56)	-0.26 (-0.69, 0.15)	0.11 (-1.19, 1.38)
bvNMA 1b	0 (-2.57, 2.59)	-0.26 (-0.62, 0.1)	0.11 (-1.24, 1.46)
bvNMA 1c	0.01 (-0.62, 0.63)	-0.26 (-0.47, -0.05)	0.11 (-0.22, 0.45)
bvNMA 1d	0 (-0.6, 0.6)	-0.26 (-0.49, -0.03)	0.11 (-0.25, 0.48)
bvNMA 2a	-0.16 (-0.64, 0.32)	-0.21 (-0.48, 0.05)	0.06 (-0.3, 0.4)
bvNMA 2b	-0.15 (-0.63, 0.34)	-0.22 (-0.46, 0.03)	0.06 (-0.31, 0.41)
bvNMA 2c	-0.09 (-0.42, 0.29)	-0.22 (-0.4, -0.04)	0.08 (-0.16, 0.33)
bvNMA 2d	-0.08 (-0.41, 0.31)	-0.21 (-0.41, -0.02)	0.08 (-0.17, 0.36)
correlations			
bvNMA 1a	0 (-0.88, 0.88)	-0.02 (-0.9, 0.88)	-0.04 (-0.92, 0.89)
bvNMA 1b	-0.05 (-0.87, 0.83)	-0.28 (-0.94, 0.73)	-0.03 (-0.84, 0.81)
bvNMA 1c	-0.37 (-0.94, 0.7)	-0.38 (-0.95, 0.68)	-0.11 (-0.84, 0.75)
bvNMA 2a	0.02 (-0.88, 0.89)	-0.03 (-0.9, 0.88)	-0.03 (-0.91, 0.89)
bvNMA 2b	-0.05 (-0.88, 0.84)	-0.3 (-0.94, 0.73)	-0.04 (-0.84, 0.8)
bvNMA 2c	-0.36 (-0.94, 0.72)	-0.39 (-0.94, 0.67)	-0.11 (-0.84, 0.75)
intercepts			
bvNMA 1a	-1.91 (-6.85, 6.32)	-0.47 (-3.09, 2.49)	-0.4 (-3.84, 1.69)
bvNMA 1b	0.06 (-3.76, 3.86)	-0.18 (-0.77, 0.41)	0.11 (-1.23, 1.44)
bvNMA 1c	0.25 (-0.76, 1.5)	-0.15 (-0.54, 0.25)	0.11 (-0.26, 0.49)
bvNMA 1d	0.36 (-0.28, 1.08)	-0.09 (-0.3, 0.12)	0.11 (-0.19, 0.41)
bvNMA 2a	-0.44 (-2.29, 1.89)	-0.31 (-2.81, 2.38)	0.22 (-0.77, 0.91)
bvNMA 2b	-0.13 (-1.25, 0.94)	-0.14 (-0.58, 0.32)	0.06 (-0.48, 0.6)
bvNMA 2c	-0.01 (-0.46, 0.56)	-0.13 (-0.46, 0.24)	0.07 (-0.22, 0.38)
bvNMA 2d	0.05 (-0.28, 0.49)	-0.07 (-0.26, 0.11)	0.06 (-0.17, 0.31)
Var(log OR) (TR)			
bvNMA 1a	1.34 (0, 3.8)	0.98 (0, 3.67)	0.74 (0, 3.47)
bvNMA 1b	1.58 (0.06, 4.92)	0.74 (0.03, 3.13)	0.93 (0.01, 3.75)
bvNMA 1c	0.24 (0.02, 0.84)	0.21 (0.02, 0.69)	0.11 (0, 0.48)
bvNMA 2a	1.23 (0, 3.76)	0.93 (0, 3.63)	0.56 (0, 3.18)
bvNMA 2b	1.47 (0.06, 4.76)	0.64 (0.03, 2.7)	0.67 (0.01, 3.34)
bvNMA 2c	0.24 (0.02, 0.8)	0.21 (0.02, 0.64)	0.09 (0, 0.4)
Var(log HR) (PFS)			
bvNMA 1a	1.34 (0, 3.8)	0.8 (0, 3.55)	0.63 (0, 3.42)
bvNMA 1b	1.36 (0.02, 3.92)	0.15 (0, 0.8)	0.7 (0, 3.48)
bvNMA 1c	0.04 (0, 0.15)	0.03 (0, 0.11)	0.02 (0, 0.1)
bvNMA 2a	0.84 (0, 3.57)	0.8 (0, 3.54)	0.32 (0, 2.48)
bvNMA 2b	0.87 (0.01, 3.62)	0.12 (0, 0.56)	0.37 (0, 2.68)
bvNMA 2c	0.03 (0, 0.13)	0.03 (0, 0.12)	0.02 (0, 0.09)

TABLE 7 Between-studies correlations and variances for each model in the aCRC example (contrasts AE, AD and CF).

8 | ILLUSTRATION USING SIMULATED DATA

8.1 | Data simulation

To demonstrate scenarios where use of bvNMA methods has an advantage over the standard surrogacy models, data were simulated under different assumptions. In particular, we simulated data where the surrogate pattern across all studies and treatments differed from the patterns within treatment contrasts, which is detectable by mvNMA but not by BRMA. The treatment effects on two outcomes were simulated from a bivariate normal distribution:

$$\begin{pmatrix} Y_{1kli} \\ Y_{2kli} \end{pmatrix} \sim \mathrm{N}\left\{ \begin{pmatrix} d_{1kl} \\ d_{2kl} \end{pmatrix}, \begin{pmatrix} \sigma^2_{1kli} + \tau^2_{1kl} & \sigma_{1kli}\sigma_{2kli}\rho_{wkli} + \tau_{1kl}\tau_{2kl}\rho_{1kl,2kl} \\ \sigma_{1kli}\sigma_{2kli}\rho_{wkli} + \tau_{1kl}\tau_{2kl}\rho_{1kl,2kl} & \sigma^2_{2kli} + \tau^2_{2kl} \end{pmatrix} \right\},$$

as in model 1a. Two sets of network data were generated, each consisting of 30 studies, three treatments and three treatment contrasts with 10 studies per contrast (AB, BC and AC), under different scenarios (illustrated in Figure 5).

Scenario 1 was simulated assuming weak study-level surrogacy when ignoring treatment contrasts but strong study-level surrogacy within each treatment contrast, with the following parameters: $\mathbf{d}_{AB} = (1,2), \mathbf{d}_{BC} = (2,1), \mathbf{d}_{AC} = (3,3); \sigma_{jAB(AC,BC)i} \sim Unif(0.15, 0.25)$, j=1,2; $\rho_{wABi} = \rho_{wBCi} = \rho_{wACi} = 0.6$; $\tau_{1AB} = 0.3$, $\tau_{1BC} = \tau_{1AC} = 0.6$, $\tau_{2BC} = 0.3$, $\tau_{2AB} = \tau_{2AC} = 0.6$; $\rho_{AB} = \rho_{AC} = \rho_{BC} = 0.98$.

Scenario 2 was simulated assuming a strong study-level surrogacy relationship when ignoring treatment contrasts as well as a strong surrogate relationships within each treatment contrast, with the following parameters: $\mathbf{d}_{AB} = (1,1)$, $\mathbf{d}_{BC} = (2,2)$, $\mathbf{d}_{AC} = (3,3)$; $\sigma_{jAB(AC,BC)i} \sim Unif(0.05, 0.15)$, j=1,2; $\rho_{wABi} = \rho_{wBCi} = \rho_{wACi} = 0.98$; $\tau_{1AB} = 0.2$, $\tau_{1BC} = 0.25$, $\tau_{1AC} = 0.3$, $\tau_{2AB} = 0.3$, $\tau_{2BC} = 0.25$, $\tau_{2AC} = 0.2$; $\rho_{AB} = \rho_{AC} = \rho_{BC} = 0.98$.

Scenario 3 was simulated assuming weak study-level surrogacy when ignoring treatment contrasts as well as within each treatment contrast, with the following parameters: $\mathbf{d}_{AB} = (1,2)$, $\mathbf{d}_{BC} = (2,1)$, $\mathbf{d}_{AC} = (3,3)$; $\sigma_{jAB(AC,BC)i} \sim Unif(0.15, 0.25)$, j=1,2; $\rho_{wABi} = \rho_{wBCi} = \rho_{wACi} = 0.6$; $\tau_{1AB} = \tau_{1BC} = \tau_{1AC} = \tau_{2BC} = \tau_{2AB} = \tau_{2AC} = 0.4$; $\rho_{AB} = \rho_{AC} = \rho_{BC} = 0.0$.

Scenario 4 was simulated assuming a strong study-level surrogacy relationship when ignoring treatment contrasts but no study-level surrogate relationship within each treatment contrast, with the following parameters: $\mathbf{d}_{AB} = (1,1)$, $\mathbf{d}_{BC} = (2,2)$, $\mathbf{d}_{AC} = (3,3)$; $\sigma_{jAB(AC,BC)i} \sim Unif(0.05, 0.15)$, j=1,2; $\rho_{wABi} = \rho_{wBCi} = \rho_{wACi} = 0.6$; $\tau_{1AB} = 0.2$, $\tau_{1BC} = 0.25$, $\tau_{1AC} = 0.3$, $\tau_{2AB} = 0.3$, $\tau_{2BC} = 0.25$, $\tau_{2AC} = 0.2$; $\rho_{AB} = \rho_{AC} = \rho_{BC} = 0.0$.

8.2 | Results of the analysis of the simulated data

Table 8 shows the between-studies correlations obtained by applying all models to the data simulated under all four scenarios. Additional parameters, that include the heterogeneity parameters and the implied intercepts, obtained from these analyses of all the simulated data scenarios are shown in Tables 10–13. Table 9 shows a range of statistics (described in Section 4.3.1 in the main manuscript) comparing the models in terms of their value in predicting the treatment effect on the final outcome from the treatment effect measured on the surrogate endpoint in a cross-validation procedure in all four scenarios. The results from each scenario are discussed in turn in the following sections.

8.2.1 | Scenario 1

As shown in the top part of Table 8, the between-studies correlation obtained from BRMA (across all studies) was not very high: 0.57 (95% CrI: 0.25, 0.77). Bivariate NMA with the covariance matrix varying across treatment contrasts models the data in more detail and reveals strong correlation between outcomes within the treatment contrasts, namely 0.79 (0.23, 0.99) for treatment contrast AB, 0.73 (0.01, 0.99) for BC and 0.84 (0.47, 0.98) for AC when using model 1a. Placing additional constraints on the covariance matrix by assuming second order consistency in models 1b and 1c reduced uncertainty around the correlations. Model 1d resulted in a common correlation obtained with the highest precision, however it did not take into account the differences in the between-studies variances across the treatment contrasts, in contrast to models 1a-c. The heterogeneity parameters are presented in Table 10 along with the implied intercepts, whose intervals include zero for all models apart from BRMA and models 1d and 2d (for some contrasts) which also ignore the differences in the heterogeneity patterns of the data. The right-hand-side column of Table 8 shows the across-treatment correlations ρ_t indicating a weak treatment-level surrogate relationship. This is due to the differences in the study-level surrogacy patterns across the treatment contrasts as well as the small number of treatment contrasts.

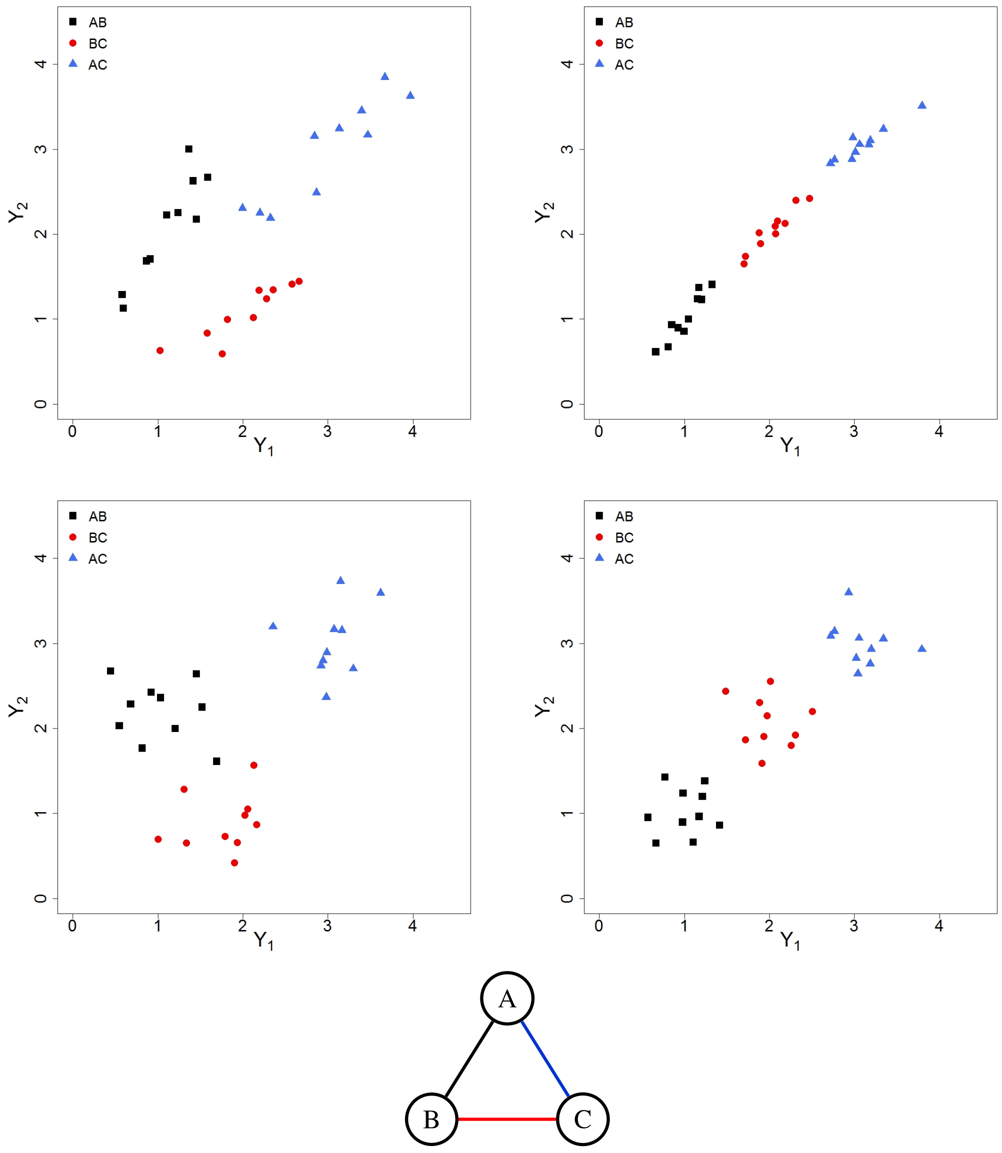

FIGURE 5 Scatter plots of the artificial data simulated under scenario 1 (top left), scenario 2 (top right), scenario 3 (bottom left), scenario 4 (bottom right)and network diagram corresponding to the structure of data for both scenarios.

The top part of Table 9 lists a range of statistics indicating the superiority of the NMA methods in terms of their predictive value when applying the methods in a cross-validation procedure to data from scenario 1. The large width of the predicted interval obtained from BRMA compared to the width of the CI of the observed treatment effect estimate is due to high uncertainty, but the ratio w_{pred}/w_{obs} is reduced when using NMA models. Predicted intervals obtained from NMA models are between 50% and 58% narrower compared to those obtained from BRMA. The distance between the point estimate of the observed effect from the predicted effect is also much reduced when using NMA models compared to BRMA. Figure 6 shows predicted effects obtained (a) using BRMA and (b) from model 1a obtained with higher precision.

8.2.2 | Scenario 2

The second section of Table 8 shows the between-studies correlations for the data simulated under scenario 2. The overall correlation obtained from BRMA is high: 0.99 (95% CrI: 0.98, 1.00). Bivariate NMA with the variance-covariance matrix varying across treatment contrasts resulted in high correlations within each treatment contrast, but obtained with much higher uncertainty compared to BRMA, due to fewer data points within each treatment contrast. The heterogeneity patterns in this scenario were similar within each treatment contrast and the whole data set. The heterogeneity parameters are shown in Table 11 along with the implied intercepts which in this scenario have intervals containing zero for BRMA and most NMA models (apart from 1d and 2d which ignore some subtle differences in the heterogeneity patterns across the treatment contrasts) The across-treatment correlations ρ_t in the right-hand-side column of Table 8 are obtained with high uncertainty due to the small number of treatment contrasts to estimate the correlation.

The second section of Table 9 shows the statistics for the model comparison in terms of their predictive value, obtained from the cross-validation procedure. In this scenario, the results obtained from the NMA based models appear similar to those from BRMA due to the strong study-level surrogacy across all studies as well as within each treatment contrast. Predicted intervals from NMA models were obtained with only a modest improvement in precision (likely resulting from additional borrowing of information through the indirect effects), with the reduction in uncertainty between 2% and 9% obtained from BRMA. The distance between the point estimate of the observed effect and the predicted effect is slightly increased when using NMA models 1a-c and 2a-c compared to BRMA, due to reduced data within treatment contrasts.

8.2.3 | Scenario 3

The third section of Table 8 shows the between-studies correlations for the data simulated under scenario 3, which indicate the lack of surrogate relationship at the study level overall (BRMA) and within each treatment contrast (NMA models) as well as at the treatment level. Heterogeneity parameters and the implied intercepts are shown for completeness in Table 12.

When the surrogate relationship is weak, in practice the cross validation procedure is not carried out. However, for completeness and the methodological considerations we present the relevant statistics in Table 9, which for scenario 3 are in the third section of the table. The absolute bias of the predicted effects was reduced by over 50% when using the NM models compared to BRMA. In addition the uncertainty around the predicted effects was also reduced by between 52% and 61%. The assumption of normality of all data across all studies made in BRMA is unlikely to be satisfied in this data scenario, whereas assuming separate bivariate normal distributions for each subset of data within each treatment contrast, as in the NMA models, is much more plausible. This more detailed modelling of the distribution of the data, along with the added borrowing of information through indirect effects, leads to better predictions when using NMA modelling approach.

8.2.4 | Scenario 4

The bottom section of Table 8 shows the between-studies correlations for the data simulated under scenario 4, whilst the heterogeneity parameters and the implied intercepts are shown in Table 13. The correlation obtained from BRMA is very high; 0.86 (0.73, 0.94). However, as expected, the correlations within each contrast indicate no surrogate relationship at the study level within these contrasts. Also the interval of the implied intercept obtained from BRMA includes zero, but not the intervals obtained from the NMA models due to the lack of correlation (lack of slope in the data results in positive intercepts for all contrasts).

The bottom part of Table 9 shows the statistics for model comparison obtained from the cross-validation procedure. Despite the lack of correlation within each treatment contrast but high correlation across all studies, the predictions obtained from the NMA models were obtained with higher precision compared to BRMA reducing the absolute bias by 30% and the uncertainty around the predicted effect by between 28% and 39%. This is largely due to the impact of the indirect effects contributing to the predicted effects as well as more detailed modelling technique as explained in scenario 3.

model	*Between-studies correlations* AB	BC	AC	ρ_t
scenario 1				
BRMA		0.54 (0.25, 0.77)		NA
bvNMA 1a	0.79 (0.23, 0.99)	0.73 (0.01, 0.99)	0.84 (0.47, 0.98)	NA
bvNMA 1b	0.87 (0.55, 0.99)	0.73 (0.14, 0.97)	0.89 (0.66, 0.99)	NA
bvNMA 1c	0.85 (0.49, 0.99)	0.68 (0.04, 0.96)	0.88 (0.63, 0.98)	NA
bvNMA 1d		0.88 (0.71, 0.97)		NA
bvNMA 2a	0.80 (0.27, 0.99)	0.73 (0.03, 0.99)	0.84 (0.44, 0.98)	0.46 (-0.30, 0.93)
bvNMA 2b	0.88 (0.56, 0.99)	0.73 (0.15, 0.97)	0.89 (0.65, 0.99)	0.44 (-0.34, 0.93)
bvNMA 2c	0.86 (0.50, 0.99)	0.67 (0.03, 0.96)	0.87 (0.63, 0.98)	0.45 (-0.34, 0.93)
bvNMA 2d		0.88 (0.72, 0.97)		0.47 (-0.29, 0.93)
scenario 2				
BRMA		0.99 (0.98, 1)		NA
bvNMA 1a	0.8 (0.29, 0.97)	0.79 (0.16, 0.97)	0.89 (0.61, 0.99)	NA
bvNMA 1b	0.82 (0.44, 0.97)	0.83 (0.36, 0.98)	0.89 (0.69, 0.98)	NA
bvNMA 1c	0.77 (0.28, 0.96)	0.76 (0.18, 0.97)	0.87 (0.65, 0.98)	NA
bvNMA 1d		0.86 (0.72, 0.94)		NA
bvNMA 2a	0.79 (0.26, 0.97)	0.81 (0.32, 0.97)	0.89 (0.6, 0.99)	0.61 (-0.19, 0.99)
bvNMA 2b	0.82 (0.44, 0.97)	0.83 (0.35, 0.98)	0.89 (0.69, 0.98)	0.60 (-0.22, 0.99)
bvNMA 2c	0.76 (0.27, 0.96)	0.74 (0.15, 0.97)	0.87 (0.66, 0.98)	0.55 (-0.31, 0.99)
bvNMA 2d		0.86 (0.71, 0.94)		0.53 (-0.26, 0.99)
scenario 3				
BRMA		0.4 (0.05, 0.68)		NA
bvNMA 1a	-0.44 (-0.94, 0.31)	-0.14 (-0.88, 0.61)	0.08 (-0.62, 0.7)	NA
bvNMA 1b	-0.42 (-0.92, 0.31)	-0.09 (-0.75, 0.58)	0.01 (-0.65, 0.62)	NA
bvNMA 1c	-0.5 (-0.94, 0.15)	-0.17 (-0.79, 0.48)	-0.07 (-0.69, 0.53)	NA
bvNMA 1d		-0.21 (-0.68, 0.28)		NA
bvNMA 2a	-0.43 (-0.94, 0.31)	-0.12 (-0.84, 0.62)	0.07 (-0.62, 0.7)	0.45 (-0.33, 0.93)
bvNMA 2b	-0.43 (-0.93, 0.29)	-0.09 (-0.75, 0.58)	0 (-0.65, 0.63)	0.46 (-0.32, 0.93)
bvNMA 2c	-0.5 (-0.94, 0.17)	-0.16 (-0.78, 0.49)	-0.06 (-0.69, 0.55)	0.45 (-0.32, 0.93)
bvNMA 2d		-0.21 (-0.68, 0.28)		0.45 (-0.31, 0.93)
scenario 4				
BRMA		0.86 (0.73, 0.94)		NA
bvNMA 1a	-0.04 (-0.66, 0.58)	-0.34 (-0.84, 0.34)	-0.27 (-0.78, 0.38)	NA
bvNMA 1b	-0.1 (-0.69, 0.52)	-0.32 (-0.8, 0.3)	-0.3 (-0.78, 0.32)	NA
bvNMA 1c	-0.12 (-0.68, 0.46)	-0.34 (-0.79, 0.23)	-0.32 (-0.77, 0.25)	NA
bvNMA 1d		-0.3 (-0.65, 0.12)		NA
bvNMA 2a	-0.05 (-0.66, 0.57)	-0.34 (-0.84, 0.32)	-0.27 (-0.79, 0.38)	0.55 (-0.27, 0.98)
bvNMA 2b	-0.11 (-0.69, 0.52)	-0.32 (-0.8, 0.3)	-0.3 (-0.78, 0.32)	0.58 (-0.23, 0.99)
bvNMA 2c	-0.12 (-0.68, 0.46)	-0.34 (-0.79, 0.23)	-0.31 (-0.77, 0.26)	0.58 (-0.23, 0.99)
bvNMA 2d		-0.29 (-0.64, 0.12)		0.57 (-0.23, 0.99)

TABLE 8 Between-studies correlations, representing study-level surrogacy, for each model under all simulation scenarios. ρ_t is the correlation corresponding to the treatment-level surrogate relationship.Where only one value is given (models BRMA, 1d and 2d), the parameters are common across the treatment contrasts.

	$p_{overlap}$	$\lvert m_{obs} - m_{pred} \rvert$	w_{pred}/w_{obs}	π	$\%red.$
scenario 1					
BRMA	0.98	0.69	4.11	0.24	
bvNMA 1a	0.97	0.23	2.05	0.50	50.24
bvNMA 1b	0.96	0.23	1.85	0.53	54.84
bvNMA 1c	0.94	0.25	1.73	0.56	57.89
bvNMA 1d	0.95	0.24	1.76	0.54	57.12
bvNMA 2a	0.97	0.23	2.04	0.50	50.35
bvNMA 2b	0.96	0.23	1.86	0.53	54.80
bvNMA 2c	0.94	0.25	1.73	0.56	57.85
bvNMA 2d	0.95	0.24	1.76	0.54	57.08
scenario 2					
BRMA	0.97	0.09	1.9	0.52	
bvNMA 1a	0.97	0.11	1.86	0.54	2.33
bvNMA 1b	0.97	0.10	1.83	0.54	4.30
bvNMA 1c	0.94	0.11	1.74	0.56	9.01
bvNMA 1d	0.95	0.11	1.76	0.55	7.73
bvNMA 2a	0.97	0.11	1.86	0.54	2.35
bvNMA 2b	0.97	0.10	1.82	0.55	4.53
bvNMA 2c	0.94	0.11	1.74	0.55	8.95
bvNMA 2d	0.95	0.11	1.76	0.55	7.69
scenario 3					
BRMA	0.99	0.79	4.8	0.21	
bvNMA 1a	0.92	0.34	2.25	0.42	53.13
bvNMA 1b	0.92	0.34	2.17	0.43	54.59
bvNMA 1c	0.87	0.34	1.87	0.47	60.84
bvNMA 1d	0.90	0.33	1.98	0.46	58.46
bvNMA 2a	0.92	0.34	2.25	0.42	52.99
bvNMA 2b	0.92	0.34	2.18	0.43	54.51
bvNMA 2c	0.88	0.34	1.88	0.47	60.70
bvNMA 2d	0.90	0.33	1.98	0.46	58.43
scenario 4					
BRMA	0.96	0.34	5.1	0.2	
bvNMA 1a	0.92	0.24	3.76	0.26	27.69
bvNMA 1b	0.92	0.24	3.58	0.27	31
bvNMA 1c	0.89	0.24	3.15	0.3	39.19
bvNMA 1d	0.91	0.24	3.12	0.31	39.21
bvNMA 2a	0.92	0.24	3.76	0.26	27.78
bvNMA 2b	0.92	0.24	3.57	0.27	31.1
bvNMA 2c	0.89	0.24	3.15	0.3	39.23
bvNMA 2d	0.91	0.24	3.12	0.31	39.19

TABLE 9 Comparison of models based on all simulation scenarios.

model	AB	BC	AC
heterogeneity parameters for surrogate endpoint Y_1			
BRMA		0.91 (0.52, 1.57)	
bvNMA 1a	0.11 (0.01, 0.33)	0.21 (0.05, 0.62)	0.46 (0.15, 1.16)
bvNMA 1b	0.12 (0.03, 0.36)	0.21 (0.07, 0.49)	0.43 (0.18, 0.87)
bvNMA 1c	0.07 (0.02, 0.2)	0.15 (0.05, 0.32)	0.29 (0.13, 0.55)
bvNMA 1d		0.24 (0.12, 0.43)	
bvNMA 2a	0.11 (0.01, 0.37)	0.22 (0.05, 0.62)	0.45 (0.15, 1.12)
bvNMA 2b	0.13 (0.03, 0.35)	0.21 (0.07, 0.5)	0.43 (0.18, 0.86)
bvNMA 2c	0.08 (0.02, 0.2)	0.14 (0.05, 0.29)	0.28 (0.13, 0.53)
bvNMA 2d		0.24 (0.12, 0.42)	
heterogeneity parameters for final outcome Y_2			
BRMA		0.93 (0.53, 1.60)	
bvNMA 1a	0.35 (0.11, 0.96)	0.07 (0, 0.25)	0.39 (0.13, 1.00)
bvNMA 1b	0.30 (0.12, 0.66)	0.08 (0.01, 0.26)	0.42 (0.17, 0.87)
bvNMA 1c	0.20 (0.08, 0.4)	0.05 (0.00, 0.16)	0.28 (0.12, 0.54)
bvNMA 1d		0.24 (0.12, 0.44)	
bvNMA 2a	0.36 (0.11, 0.98)	0.07 (0.00, 0.24)	0.38 (0.13, 0.97)
bvNMA 2b	0.30 (0.12, 0.66)	0.09 (0.01, 0.27)	0.42 (0.16, 0.86)
bvNMA 2c	0.20 (0.08, 0.42)	0.05 (0.01, 0.15)	0.27 (0.12, 0.53)
bvNMA 2d		0.24 (0.12, 0.44)	
intercepts λ_0			
BRMA		0.9 (0.16, 1.62)	
bvNMA 1a	0.32 (-1.41, 1.58)	0.2 (-0.69, 1.09)	0.65 (-0.52, 1.93)
bvNMA 1b	0.42 (-0.64, 1.23)	0.17 (-0.64, 0.98)	0.33 (-0.59, 1.31)
bvNMA 1c	0.39 (-0.83, 1.29)	0.27 (-0.52, 1.07)	0.43 (-0.48, 1.42)
bvNMA 1d	1.07 (0.76, 1.38)	-0.77 (-1.29, -0.24)	0.3 (-0.49, 1.08)
bvNMA 2a	0.25 (-1.44, 1.5)	0.22 (-0.67, 1.12)	0.66 (-0.48, 1.97)
bvNMA 2b	0.41 (-0.67, 1.22)	0.16 (-0.66, 0.97)	0.33 (-0.59, 1.32)
bvNMA 2c	0.38 (-0.78, 1.24)	0.27 (-0.54, 1.08)	0.41 (-0.49, 1.38)
bvNMA 2d	1.06 (0.76, 1.37)	-0.77 (-1.29, -0.24)	0.29 (-0.48, 1.08)

TABLE 10 Heterogeneity parameters and intercepts for each model under simulation scenario 1. Where only one value is given (models BRMA, 1d and 2d), the parameters are common across the treatment contrasts.

model	AB	BC	AC
heterogeneity parameters for surrogate endpoint Y_1			
BRMA		0.82 (0.49, 1.35)	
bvNMA 1a	0.03 (0, 0.08)	0.04 (0.01, 0.11)	0.1 (0.04, 0.25)
bvNMA 1b	0.03 (0.01, 0.1)	0.06 (0.01, 0.17)	0.08 (0.03, 0.16)
bvNMA 1c	0.02 (0, 0.06)	0.04 (0.01, 0.1)	0.06 (0.03, 0.11)
bvNMA 1d		0.06 (0.03, 0.1)	
bvNMA 2a	0.03 (0, 0.08)	0.04 (0.01, 0.12)	0.1 (0.04, 0.26)
bvNMA 2b	0.03 (0.01, 0.1)	0.06 (0.01, 0.17)	0.08 (0.03, 0.15)
bvNMA 2c	0.02 (0, 0.06)	0.04 (0.01, 0.1)	0.06 (0.03, 0.11)
bvNMA 2d		0.06 (0.03, 0.1)	
heterogeneity parameters for surrogate endpoint Y_2			
BRMA		0.78 (0.47, 1.29)	
bvNMA 1a	0.06 (0.01, 0.15)	0.04 (0, 0.11)	0.04 (0.01, 0.11)
bvNMA 1b	0.05 (0.02, 0.12)	0.06 (0.01, 0.16)	0.04 (0.01, 0.09)
bvNMA 1c	0.04 (0.01, 0.08)	0.04 (0.01, 0.09)	0.03 (0.01, 0.06)
bvNMA 1d		0.05 (0.02, 0.09)	
bvNMA 2a	0.06 (0.02, 0.16)	0.04 (0.01, 0.12)	0.04 (0.01, 0.11)
bvNMA 2b	0.05 (0.02, 0.12)	0.06 (0.01, 0.16)	0.04 (0.01, 0.09)
bvNMA 2c	0.04 (0.01, 0.08)	0.03 (0.01, 0.09)	0.03 (0.01, 0.06)
bvNMA 2d		0.05 (0.02, 0.09)	
intercepts λ_0			
BRMA		0.06 (-0.05, 0.16)	
bvNMA 1a	-0.37 (-1.59, 0.55)	0.43 (-0.41, 1.81)	1.34 (0.72, 2.04)
bvNMA 1b	-0.15 (-0.88, 0.48)	0.4 (-0.31, 1.45)	1.17 (0.46, 1.78)
bvNMA 1c	-0.1 (-0.94, 0.63)	0.56 (-0.2, 1.75)	1.24 (0.6, 1.84)
bvNMA 1d	0.21 (0, 0.43)	0.44 (0.03, 0.86)	0.65 (0.03, 1.28)
bvNMA 2a	-0.29 (-1.36, 0.62)	0.4 (-0.42, 1.54)	1.33 (0.69, 2.04)
bvNMA 2b	-0.15 (-0.89, 0.48)	0.41 (-0.3, 1.46)	1.17 (0.46, 1.79)
bvNMA 2c	-0.1 (-1.01, 0.65)	0.6 (-0.2, 1.8)	1.25 (0.62, 1.84)
bvNMA 2d	0.21 (0, 0.42)	0.43 (0.02, 0.86)	0.64 (0.03, 1.27)

TABLE 11 Heterogeneity parameters and intercepts for each model under simulation scenario 2. Where only one value is given (models BRMA, 1d and 2d), the parameters are common across the treatment contrasts.

model	AB	BC	AC
heterogeneity parameters for surrogate endpoint Y_1			
BRMA		0.92 (0.52, 1.6)	
bvNMA 1a	0.23 (0.06, 0.7)	0.18 (0.03, 0.58)	0.15 (0.03, 0.47)
bvNMA 1b	0.2 (0.06, 0.48)	0.19 (0.04, 0.48)	0.13 (0.03, 0.38)
bvNMA 1c	0.14 (0.04, 0.32)	0.12 (0.03, 0.31)	0.09 (0.02, 0.25)
bvNMA 1d		0.13 (0.06, 0.25)	
bvNMA 2a	0.23 (0.06, 0.68)	0.18 (0.03, 0.59)	0.15 (0.03, 0.48)
bvNMA 2b	0.19 (0.06, 0.47)	0.18 (0.04, 0.48)	0.14 (0.03, 0.38)
bvNMA 2c	0.14 (0.04, 0.33)	0.13 (0.03, 0.32)	0.1 (0.02, 0.26)
bvNMA 2d		0.13 (0.06, 0.25)	
heterogeneity parameters for surrogate endpoint Y_2			
BRMA		1.01 (0.57, 1.75)	
bvNMA 1a	0.14 (0.03, 0.44)	0.11 (0.01, 0.37)	0.22 (0.05, 0.65)
bvNMA 1b	0.14 (0.03, 0.35)	0.12 (0.02, 0.35)	0.17 (0.05, 0.4)
bvNMA 1c	0.1 (0.02, 0.24)	0.07 (0.01, 0.21)	0.11 (0.03, 0.26)
bvNMA 1d		0.11 (0.05, 0.22)	
bvNMA 2a	0.15 (0.03, 0.46)	0.11 (0.01, 0.36)	0.22 (0.05, 0.66)
bvNMA 2b	0.14 (0.03, 0.35)	0.12 (0.02, 0.35)	0.17 (0.05, 0.4)
bvNMA 2c	0.1 (0.03, 0.24)	0.08 (0.01, 0.22)	0.12 (0.04, 0.28)
bvNMA 2d		0.11 (0.05, 0.21)	
intercepts λ_0			
BRMA		1.21 (0.42, 2.01)	
bvNMA 1a	2.6 (1.86, 3.48)	1.09 (-0.19, 2.65)	2.88 (-0.58, 6.13)
bvNMA 1b	2.59 (1.86, 3.41)	1.04 (-0.14, 2.41)	3.03 (0.29, 5.84)
bvNMA 1c	2.66 (2.02, 3.44)	1.16 (0.1, 2.51)	3.32 (0.83, 6.01)
bvNMA 1d	2.41 (1.85, 3.02)	1.25 (0.36, 2.21)	3.66 (2.26, 5.19)
bvNMA 2a	2.59 (1.85, 3.48)	1.06 (-0.21, 2.57)	2.75 (-0.52, 6.17)
bvNMA 2b	2.59 (1.87, 3.4)	1.04 (-0.15, 2.43)	3.02 (0.32, 5.84)
bvNMA 2c	2.65 (2, 3.43)	1.15 (0.07, 2.53)	3.28 (0.75, 6.11)
bvNMA 2d	2.4 (1.84, 3.01)	1.25 (0.37, 2.22)	3.65 (2.25, 5.19)

TABLE 12 Heterogeneity parameters and intercepts for each model under simulation scenario 3. Where only one value is given (models BRMA, 1d and 2d), the parameters are common across the treatment contrasts.

model	AB	BC	AC
heterogeneity parameters for surrogate endpoint Y_1			
BRMA		0.88 (0.52, 1.47)	
bvNMA 1a	0.11 (0.03, 0.31)	0.12 (0.04, 0.33)	0.14 (0.05, 0.39)
bvNMA 1b	0.1 (0.03, 0.25)	0.12 (0.04, 0.29)	0.13 (0.05, 0.3)
bvNMA 1c	0.08 (0.02, 0.19)	0.09 (0.03, 0.22)	0.1 (0.04, 0.23)
bvNMA 1d		0.09 (0.05, 0.16)	
bvNMA 2a	0.1 (0.03, 0.3)	0.12 (0.04, 0.33)	0.15 (0.05, 0.41)
bvNMA 2b	0.1 (0.03, 0.25)	0.12 (0.04, 0.29)	0.13 (0.05, 0.3)
bvNMA 2c	0.07 (0.02, 0.18)	0.09 (0.03, 0.21)	0.11 (0.04, 0.23)
bvNMA 2d		0.09 (0.05, 0.16)	
heterogeneity parameters for surrogate endpoint Y_2			
BRMA		0.79 (0.47, 1.31)	
bvNMA 1a	0.1 (0.03, 0.29)	0.12 (0.03, 0.33)	0.09 (0.02, 0.28)
bvNMA 1b	0.09 (0.03, 0.23)	0.11 (0.04, 0.25)	0.09 (0.03, 0.22)
bvNMA 1c	0.07 (0.02, 0.16)	0.08 (0.03, 0.18)	0.07 (0.02, 0.16)
bvNMA 1d		0.07 (0.04, 0.14)	
bvNMA 2a	0.1 (0.03, 0.29)	0.12 (0.03, 0.33)	0.09 (0.02, 0.26)
bvNMA 2b	0.09 (0.03, 0.23)	0.11 (0.04, 0.25)	0.09 (0.03, 0.23)
bvNMA 2c	0.07 (0.02, 0.16)	0.08 (0.03, 0.18)	0.07 (0.02, 0.16)
bvNMA 2d		0.07 (0.04, 0.13)	
intercepts λ_0			
BRMA		0.37 (-0.04, 0.79)	
bvNMA 1a	1.04 (0.27, 1.84)	2.73 (1.29, 4.22)	3.69 (2, 5.48)
bvNMA 1b	1.1 (0.37, 1.85)	2.67 (1.43, 3.98)	3.8 (2.13, 5.49)
bvNMA 1c	1.12 (0.47, 1.8)	2.7 (1.58, 3.9)	3.8 (2.35, 5.28)
bvNMA 1d	1.27 (0.84, 1.69)	2.58 (1.8, 3.36)	3.85 (2.66, 5.02)
bvNMA 2a	1.04 (0.28, 1.81)	2.75 (1.34, 4.23)	3.7 (1.96, 5.48)
bvNMA 2b	1.11 (0.38, 1.87)	2.67 (1.42, 3.98)	3.79 (2.11, 5.49)
bvNMA 2c	1.12 (0.47, 1.8)	2.7 (1.58, 3.88)	3.79 (2.35, 5.26)
bvNMA 2d	1.27 (0.84, 1.69)	2.57 (1.78, 3.35)	3.84 (2.66, 5.01)

TABLE 13 Heterogeneity parameters and intercepts for each model under simulation scenario 4. Where only one value is given (models BRMA, 1d and 2d), the parameters are common across the treatment contrasts.

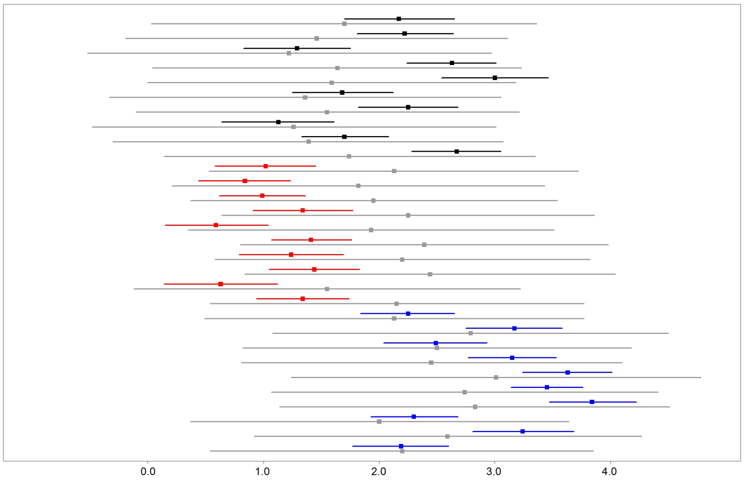

a)

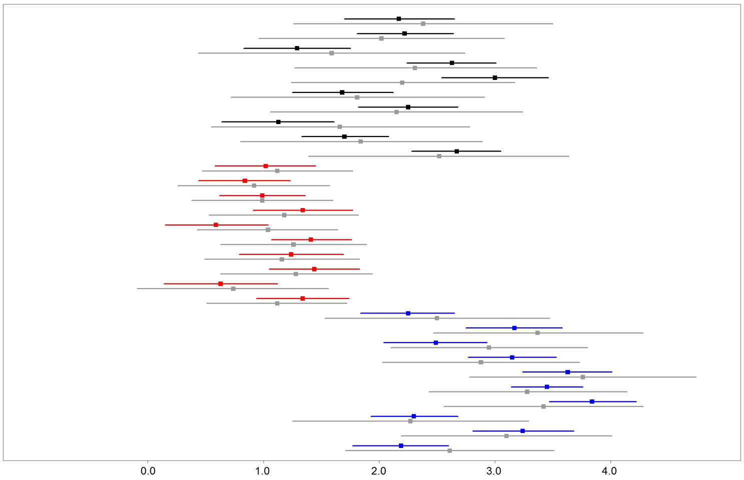

b)

FIGURE 6 Predicted effect (gray) obtained from the cross validation procedure are presented along the observed effects: black (B vs A), red (C vs B) and blue (C vs A), obtained from (a) BRMA and (b) model 1a for data simulated under scenario 1.

9 | ADDITIONAL SIMULATED SCENARIO: MIXED STRENGTH SURROGACY PATTERNS

Additional data scenario, illustrated in Figure 7, was simulated assuming a strong surrogacy relationship when ignoring treatment contrasts and a mixture of either weak or strong relationships within each treatment contrast, with the following parameters: $\mathbf{d}_{AB} = (1,1)$, $\mathbf{d}_{BC} = (2,2)$, $\mathbf{d}_{AC} = (3,3)$; $\sigma_{jAB(AC,BC)i} \sim Unif(0.05, 0.15)$, j=1,2; $\rho_{wABi} = \rho_{wBCi} = \rho_{wACi} = 0.98$; $\tau_{1AB} = 0.2$, $\tau_{1BC} = 0.25$, $\tau_{1AC} = 0.3$, $\tau_{2AB} = 0.3$, $\tau_{2BC} = 0.25$, $\tau_{2AC} = 0.2$; $\rho_{AB} = \rho_{AC} = 0.98$ and $\rho_{BC} = 0$ (strong surrogacy relationships for treatment contrasts AB and AC but no relationship for BC).

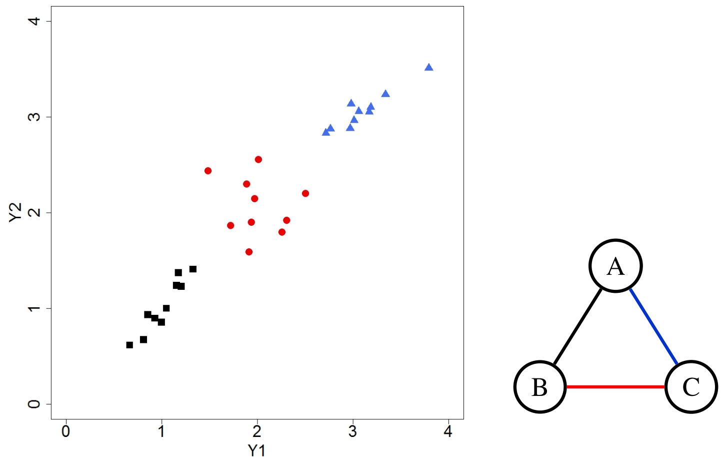

FIGURE 7 Scatter plots of the artificial data simulated under the additional mixed surrogacy scenario and corresponding network diagram.

Table 14 shows the between-studies correlations for the data simulated under this scenario. The overall correlation obtained from BRMA is high: 0.94 (95% CrI: 0.88, 0.98). Bivariate NMA with the variance-covariance matrix varying across treatment contrasts models the data in more detail and reveals no correlation between the treatment effects on the two outcomes within the BC treatment contrast. Table 15 lists the heterogeneity parameters. Figure 8 shows the predicted effects obtained from the cross-validation using (a) BRMA and (b) NMA model 1a. When using the NMA model, predictions are obtained with higher precision for contrasts AB and AC, but not BC where there was no association between the effects on the two outcomes and which is reflected by the wide predicted intervals. The across-treatment correlations ρ_t in the right-hand-side column of Table 14 are obtained with high uncertainty due to the small number of treatment contrasts to estimate the correlation. Table 16 shows the statistics for the model comparison in terms of their predictive value, obtained from the cross-validation procedure. The top panel of the table shows the statistics for all of the data. Similarly as in scenario 1, the large ratio, w_{pred}/w_{obs}, comparing the width of the predicted interval obtained from BRMA with the width of the CI of the observed treatment effect estimate is reduced when using the NMA models. Predicted intervals obtained from NMA models are between 19.6% and 29.3% narrower compared to those obtained from BRMA. The distance between the point estimate of the observed effect and the predicted effect is slightly reduced when using NMA models 1a-c and 2a-c compared to BRMA. These improvements, on average, are not as strong as in scenario 1, due to poor association for the treatment contrast BC. When investigating these statistics within the treatment contrasts, the improvement is largest for contrast AC where the correlation was highest. The three bottom panels of Table 16 show similar statistics for model comparison but separately for each treatment contrast revealing the reduced uncertainty around the predicted intervals up to 50% for contrast AC where the correlation was highest. In contrast, small reduction or even increase in uncertainty of predicted effects was noted for studies in contrast BC where the correlation was weak.

model	*within-treatment surrogate relationship* AB	BC	AC	ρ_t
BRMA		0.94 (0.88, 0.98)		NA
bvNMA 1a	0.81 (0.34, 0.99)	-0.19 (-0.73, 0.43)	0.87 (0.49, 0.99)	NA
bvNMA 1b	0.78 (0.2, 0.99)	-0.05 (-0.6, 0.53)	0.8 (0.26, 0.99)	NA
bvNMA 1c	0.77 (0.22, 0.98)	-0.11 (-0.62, 0.43)	0.79 (0.3, 0.99)	NA
bvNMA 1d		0.39 (0, 0.69)		NA
bvNMA 2a	0.81 (0.32, 0.99)	-0.2 (-0.75, 0.44)	0.87 (0.47, 0.99)	0.56 (-0.25, 0.99)
bvNMA 2b	0.77 (0.19, 0.99)	-0.04 (-0.59, 0.54)	0.79 (0.26, 0.99)	0.58 (-0.22, 0.99)
bvNMA 2c	0.78 (0.25, 0.99)	-0.11 (-0.62, 0.42)	0.79 (0.29, 0.98)	0.59 (-0.26, 0.99)
bvNMA 2d		0.39 (0, 0.69)		0.57 (-0.27, 0.99)

TABLE 14 Between-studies correlations for each model under the additional mixed surrogacy simulation scenario. Where only one value is given (models BRMA, 1d and 2d), the parameters are common across the treatment contrasts. ρ_t is the correlation corresponding to the across-treatment surrogate relationship.

model	AB	BC	AC
surrogate endpoint Y_1			
BRMA		0.85 (0.50, 1.42)	
bvNMA 1a	0.04 (0.01, 0.11)	0.12 (0.04, 0.34)	0.11 (0.04, 0.26)
bvNMA 1b	0.05 (0.01, 0.14)	0.08 (0.03, 0.17)	0.14 (0.05, 0.28)
bvNMA 1c	0.03 (0.01, 0.09)	0.06 (0.03, 0.12)	0.1 (0.05, 0.20)
bvNMA 1d		0.08 (0.04, 0.14)	
bvNMA 2a	0.04 (0.01, 0.11)	0.12 (0.04, 0.34)	0.11 (0.04, 0.28)
bvNMA 2b	0.05 (0.01, 0.13)	0.08 (0.03, 0.18)	0.14 (0.05, 0.28)
bvNMA 2c	0.03 (0.01, 0.08)	0.06 (0.03, 0.12)	0.10 (0.05, 0.20)
bvNMA 2d		0.08 (0.04, 0.14)	
final outcome Y_2			
BRMA		0.81 (0.48, 1.36)	
bvNMA 1a	0.08 (0.02, 0.21)	0.12 (0.04, 0.33)	0.04 (0.01, 0.12)
bvNMA 1b	0.12 (0.04, 0.27)	0.07 (0.03, 0.16)	0.05 (0.02, 0.12)
bvNMA 1c	0.09 (0.03, 0.19)	0.05 (0.02, 0.11)	0.04 (0.01, 0.08)
bvNMA 1d		0.07 (0.03, 0.12)	
bvNMA 2a	0.08 (0.02, 0.21)	0.12 (0.04, 0.35)	0.05 (0.01, 0.12)
bvNMA 2b	0.12 (0.04, 0.26)	0.07 (0.03, 0.16)	0.05 (0.02, 0.12)
bvNMA 2c	0.09 (0.03, 0.18)	0.05 (0.02, 0.11)	0.04 (0.01, 0.09)
bvNMA 2d		0.07 (0.03, 0.12)	

TABLE 15 Heterogeneity parameters for each model under the additional mixed surrogacy simulation scenario. Where only one value is given (models BRMA, 1d and 2d), the parameters are common across the treatment contrasts.

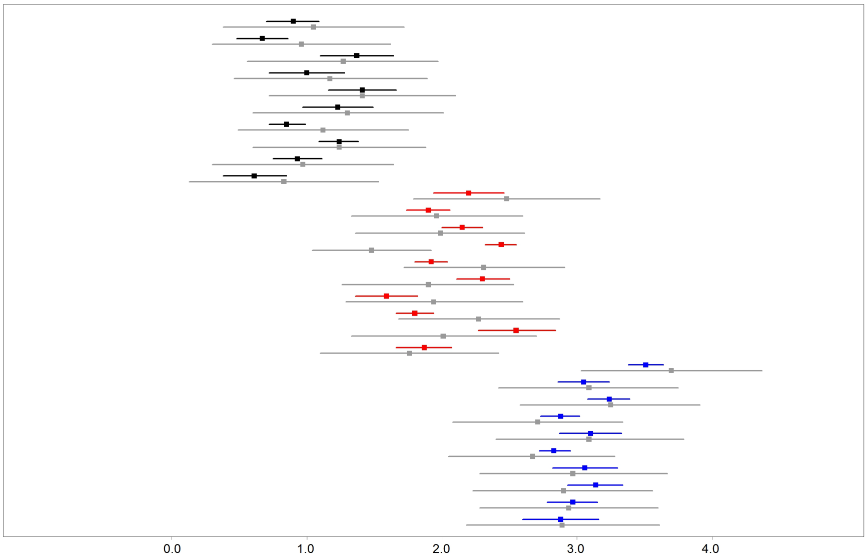

a)

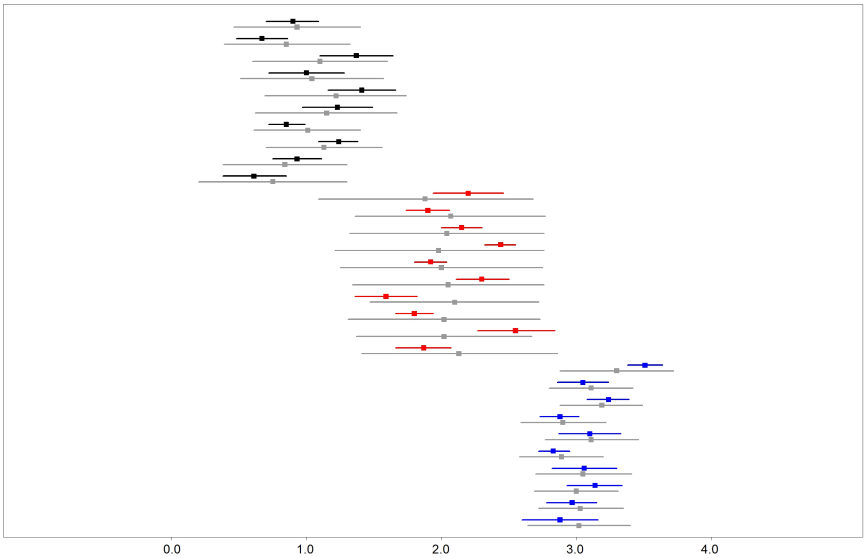

b)

FIGURE 8 Predicted effect (gray) obtained from the cross validation procedure are presented along the observed effects: black (B vs A), red (C vs B) and blue (C vs A), obtained from (a) BRMA and (b) model 1a for data simulated under scenario 2.

	$p_{overlap}$	$\|m_{obs} - m_{pred}\|$	w_{pred}/w_{obs}	π	$\%red.$
All					
BRMA	0.9	0.2	3.55	0.27	
bvNMA 1a	0.97	0.16	2.84	0.41	20.2
bvNMA 1b	0.95	0.17	2.76	0.38	21.86
bvNMA 1c	0.93	0.17	2.5	0.41	29.16
bvNMA 1d	0.9	0.2	2.87	0.33	19.5
bvNMA 2a	0.98	0.16	2.83	0.41	20.32
bvNMA 2b	0.95	0.17	2.77	0.38	21.86
bvNMA 2c	0.93	0.17	2.49	0.41	29.34
bvNMA 2d	0.9	0.2	2.87	0.33	19.61
AB					
BRMA	1	0.13	3.35	0.31	
bvNMA 1a	0.99	0.13	2.37	0.43	28.61
bvNMA 1b	1	0.11	2.81	0.37	15.44
bvNMA 1c	1	0.12	2.59	0.4	21.99
bvNMA 1d	0.98	0.19	2.63	0.39	21.53
bvNMA 2a	0.99	0.13	2.35	0.43	29.02
bvNMA 2b	1	0.11	2.82	0.37	15.23
bvNMA 2c	1	0.12	2.59	0.4	21.97
bvNMA 2d	0.98	0.19	2.63	0.39	21.63
BC					
BRMA	0.69	0.37	3.53	0.21	
bvNMA 1a	0.95	0.29	4.23	0.24	-17.64
bvNMA 1b	0.86	0.3	3.31	0.28	7.33
bvNMA 1c	0.83	0.3	2.94	0.3	17.79
bvNMA 1d	0.71	0.31	2.93	0.26	17.28
bvNMA 2a	0.95	0.29	4.22	0.24	-17.53
bvNMA 2b	0.86	0.3	3.31	0.28	7.46
bvNMA 2c	0.83	0.3	2.93	0.3	17.98
bvNMA 2d	0.71	0.31	2.92	0.26	17.47
AC					
BRMA	1	0.09	3.77	0.28	
bvNMA 1a	0.99	0.08	1.91	0.55	49.62
bvNMA 1b	1	0.09	2.17	0.49	42.81
bvNMA 1c	0.98	0.09	1.97	0.52	47.71
bvNMA 1d	1	0.11	3.05	0.35	19.67
bvNMA 2a	0.99	0.08	1.92	0.55	49.47
bvNMA 2b	1	0.09	2.17	0.49	42.88
bvNMA 2c	0.97	0.09	1.96	0.53	48.07
bvNMA 2d	1	0.1	3.04	0.35	19.74

TABLE 16 Comparison of models based on the additional mixed surrogacy simulation scenario overall and by treatment contrast.

10 | ADDITIONAL COMMENTS ON SIMULATION STUDY

In the simulation study presented in Section 6 of the main manuscript, the data were simulated from model 1a for simplicity, to allow for generating very clear and simple scenarios. Although by simulating from model 1a we do not explicitly assume second order consistency, this does not mean that the assumption is not satisfied for the simulated data and in fact the parameters we choose are such that the second order consistency is satisfied.

Scenario 1 used the following parameters: $\mathbf{d}_{AB} = (1, 2)$, $\mathbf{d}_{BC} = (2, 1)$, $\mathbf{d}_{AC} = (3, 3)$; $\sigma_{jAB(AC,BC)i} \sim Unif(0.15, 0.25)$, j=1,2; $\rho_{wABi} = \rho_{wBCi} = \rho_{wACi} = 0.6$; $\tau_{1AB} = 0.3$, $\tau_{1BC} = \tau_{1AC} = 0.6$, $\tau_{2BC} = 0.3$, $\tau_{2AB} = \tau_{2AC} = 0.6$; $\rho_{AB} = \rho_{AC} = \rho_{BC} = 0.9$.

If we denote the indexes b, k, l, for treatments as in models 1a and 1b, as $b = A$, $k = B$ and $l = C$, then the first order consistency (4)says

$$\begin{pmatrix} d_{1BC} \\ d_{2BC} \end{pmatrix} = \begin{pmatrix} d_{1AC} - d_{1AB} \\ d_{2AC} - d_{2AB} \end{pmatrix} \tag{17}$$

The parameters used give: $d_{1AC} - d_{1AB} = 3 - 1 = 2 = d_{1BC}$ and $d_{2AC} - d_{2AB} = 3 - 2 = 1 = d_{2BC}$.

For the second order consistency (8) we have:

$$|\tau_{jAC} - \tau_{jAB}| \leq \tau_{jBC} \leq \tau_{jAC} + \tau_{jAB}. \tag{18}$$

For the first outcome we have:

$$\begin{aligned} |0.6 - 0.3| &\leq 0.6 \leq 0.6 + 0.3 \\ 0.3 &\leq 0.6 \leq 0.9 \end{aligned}$$

and for the second outcome we have:

$$\begin{aligned} |0.6 - 0.6| &\leq 0.3 \leq 0.6 + 0.6 \\ 0.0 &\leq 0.6 \leq 1.2 \end{aligned}$$

In addition, the condition (9) applies to the covariances:

$$\tau_{1BC}\tau_{2BC}\rho_{1BC,2BC} = \tau_{1AC}\tau_{2AC}\rho_{1AC,2AC} + \tau_{1AB}\tau_{2AB}\rho_{1AB,2AB} - \tau_{1AC}\tau_{2AB}\rho_{1AC,2AB} - \tau_{1AB}\tau_{2AC}\rho_{1AB,2AC}, \tag{19}$$

We start from the right hand side and re-arrange terms using transitivity equation (3) (dropping study index i):

$$\begin{aligned} RHS &= cov(\mu_{1AC}, \mu_{2AC}) + cov(\mu_{1AB}, \mu_{2AB}) - cov(\mu_{1AC}, \mu_{2AB}) - cov(\mu_{1AB}, \mu_{2AC}) = \\ &= cov(\mu_{1AC}, \mu_{2AC}) + cov(\mu_{1AB}, \mu_{2AB}) - cov(\mu_{1AC}, \mu_{2AB}) - cov(\mu_{1AB}, (\mu_{2AB} + \mu_{2BC})) = \\ &= cov(\mu_{1AC}, \mu_{2AC}) + \underline{cov(\mu_{1AB}, \mu_{2AB})} - cov(\mu_{1AC}, \mu_{2AB}) - \underline{cov(\mu_{1AB}, \mu_{2AB})} - cov(\mu_{1AB}, \mu_{2BC}) = \\ &= cov(\mu_{1AC}, \mu_{2AC}) - cov(\mu_{1AC}, \mu_{2AB}) - cov((\mu_{1AC} - \mu_{1BC}), \mu_{2BC}) \qquad (20) \\ &= cov(\mu_{1AC}, \mu_{2AC}) - cov(\mu_{1AC}, \mu_{2AB}) - cov(\mu_{1AC}, \mu_{2BC}) + cov(\mu_{1BC}, \mu_{2BC}) \qquad (21) \\ &= cov(\mu_{1BC}, \mu_{2BC}) + cov(\mu_{1AC}, \mu_{2AC}) - cov(\mu_{1AC}, \mu_{2AB}) - cov(\mu_{1AC}, (\mu_{2AC} - \mu_{2AB}) \\ &= cov(\mu_{1BC}, \mu_{2BC}) + \underline{cov(\mu_{1AC}, \mu_{2AC})} - \underline{cov(\mu_{1AC}, \mu_{2AB})} - \underline{cov(\mu_{1AC}, \mu_{2AC})} + \underline{cov(\mu_{1AC}, \mu_{2AB})} \\ &= cov(\mu_{1BC}, \mu_{2BC}) \\ &= LHS \end{aligned}$$

Therefore, although we do not make any assumptions about the second-order consistency when simulating data, this condition is satisfied provided that the transitivity assumption is valid.

In a similar way, the consistency assumptions can be checked for other simulated scenarios.

References

1. Hurwitz Herbert, Fehrenbacher Louis, Novotny William, et al. Bevacizumab plus irinotecan, fluorouracil, and leucovorin for metastatic colorectal cancer. *New England journal of medicine.* 2004;350(23):2335–2342.

□
